# Cross-site and cross-platform variability of automated patch clamp assessments of drug effects on human cardiac currents in recombinant cells

**DOI:** 10.1038/s41598-020-62344-w

**Published:** 2020-03-27

**Authors:** James Kramer, Herbert M. Himmel, Anders Lindqvist, Sonja Stoelzle-Feix, Khuram W. Chaudhary, Dingzhou Li, Georg Andrees Bohme, Matthew Bridgland-Taylor, Simon Hebeisen, Jingsong Fan, Muthukrishnan Renganathan, John Imredy, Edward S. A. Humphries, Nina Brinkwirth, Tim Strassmaier, Atsushi Ohtsuki, Timm Danker, Carlos Vanoye, Liudmila Polonchuk, Bernard Fermini, Jennifer Beck Pierson, Gary Gintant

**Affiliations:** 1 Charles River Laboratories, Cleveland, OH USA; 2 Safety Pharmacology, Bayer AG, Wuppertal, Germany; 3 Sophion Bioscience A/S, Ballerup, Denmark; 4 Nanion Technologies GmbH, Munich, Germany; 5 Global Safety Pharmacology, GlaxoSmithKline PLC, Collegeville, PA USA; 6 Drug Safety Research & Development, Pfizer, Groton, CT USA; 7 Integrated Drug Discovery, High Content Biology Unit, Sanofi R&D, Vitry-Sur-Seine, France; 8 Clinical Pharmacology & Safety Sciences, BioPharmaceuticals R&D, Astra Zeneca, Cambridge, UK; 9 B’SYS GmbH, Witterswil, Switzerland; 10 Discovery Toxicology, Bristol-Myers Squibb Company, Princeton, NJ USA; 11 Eurofins Discovery, St. Charles, MO USA; 12 Merck & Co., Inc., Kenilworth, NJ USA; 13 Metrion Biosciences Ltd., Cambridge, UK; 14 Nanion Technologies, Livingston, NJ USA; 15 Nanion Technologies Japan K.K., Shinjyuku-ku, Tokyo Japan; 16 Natural and Medical Science Institute at the University of Tübingen, Reutlingen, Germany; 17 Northwestern Feinberg School of Medicine, Chicago, IL USA; 18 Roche Pharma Research & Early Development, Roche Innovation Center Basel, F. Hoffmann-La Roche Ltd., Basel, Switzerland; 19 Novoheart, Vancouver, BC Canada; 20 Health and Environmental Sciences Institute, Washington, DC USA; 21 AbbVie, Chicago, IL USA

**Keywords:** Drug safety, Drug screening, Pharmacology, Drug discovery, Cardiology

## Abstract

Automated patch clamp (APC) instruments enable efficient evaluation of electrophysiologic effects of drugs on human cardiac currents in heterologous expression systems. Differences in experimental protocols, instruments, and dissimilar site procedures affect the variability of IC_50_ values characterizing drug block potency. This impacts the utility of APC platforms for assessing a drug’s cardiac safety margin. We determined variability of APC data from multiple sites that measured blocking potency of 12 blinded drugs (with different levels of proarrhythmic risk) against four human cardiac currents (hERG [I_Kr_], hCav1.2 [L-Type I_Ca_], peak hNav1.5, [Peak I_Na_], late hNav1.5 [Late I_Na_]) with recommended protocols (to minimize variance) using five APC platforms across 17 sites. IC_50_ variability (25/75 percentiles) differed for drugs and currents (e.g., 10.4-fold for dofetilide block of hERG current and 4-fold for mexiletine block of hNav1.5 current). Within-platform variance predominated for 4 of 12 hERG blocking drugs and 4 of 6 hNav1.5 blocking drugs. hERG and hNav1.5 block. Bland-Altman plots depicted varying agreement across APC platforms. A follow-up survey suggested multiple sources of experimental variability that could be further minimized by stricter adherence to standard protocols. Adoption of best practices would ensure less variable APC datasets and improved safety margins and proarrhythmic risk assessments.

## Introduction

The characterization of drug effects on human currents using automated patch clamp (APC) platforms employing single- or multi-hole planar patch techniques^1–5^ has revolutionized the assessment of electrophysiologic (and potential proarrhythmic) effects of new drug candidates. This increasingly popular experimental approach represents the next generation of ionic current screening efforts utilizing commercially available platforms (some with higher throughput capabilities) applicable to drug studies related to cardiac (as well as neuronal) safety and efficacy.

There remains a need for *in vitro* predictive assays to characterize the proarrhythmic risk of novel drug candidates. Much effort has been focused on screening drug candidates against an important cardiac K^+^ repolarizing current (hERG, encoded by the hKv11.1 gene responsible for the I_Kr_ current) linked to delayed repolarization and Torsades-de-Pointes proarrhythmia (see the review by Roden^6^). The adoption of APC platforms replacing manual patch clamp experiments) continues to guide drug safety evaluations based on safety margin calculations relating potency of current block to clinical drug exposures and delayed cardiac repolarization (manifest as QT prolongation on the electrocardiogram). More recently, *in silico* modeling of integrated electrophysiologic responses to drugs (based on *in vitro* ionic current assay results) is being tested to assess proarrhythmic risk within the Comprehensive *In Vitro* Proarrhythmia Assay (CiPA) paradigm^7–10^. This initiative relies on APC platforms to efficiently provide reliable and reproducible estimates of the potency of drug block of multiple currents that modulate repolarization and affect proarrhythmic risk, including (a) hERG, (b) peak hNav1.5 and (c) late hNav1.5 (both encoded by the SCN5A gene), and (d) hCav1.2 (encoded by the human CACNA1C gene and co-expressed with the β2 subunit, encoded by the human CACNB2 gene and the α2δ1 subunit encoded by the human CACNA2D1 gene responsible for I_Ca,L_, high threshold calcium current). A drug’s effects on multiple human cardiac currents can be used to calculate the net effect on membrane current that defines human ventricular repolarization using integrating *in silico* models to predict torsadogenic risk^9,10^.

Studies have characterized the potency of current block by drugs using IC_50_ values across a few APC platforms^11^. However, a study evaluating the extent of variability of IC_50_ determinations within (and between) multiple APC platforms, across multiple currents, and across multiple drugs has not been reported. This information is essential when comparing drug effects across platforms, in translating *in vitro* findings to clinical effects, as well as in interpreting drug effects across platforms during lead identification and optimization campaigns and for regulatory assessments.

To evaluate the strengths and limitations of using APC platforms to characterize drug block of human cardiac currents, a pilot study was conducted to assess the variability of IC_50_ values characterizing block potency of 12 single-sourced blinded drugs (linked to high, intermediate, and low proarrhythmic risk) on seven human cardiac currents expressed in heterologous expression systems using five APC platforms. Consensus voltage clamp command waveforms and experimental protocols were suggested to construct and compare variability of concentration-response curves for 12 drugs from up to 17 experimental sites.

This manuscript presents results from studies on four prominent human cardiac currents that modulate cardiac repolarization (hERG, peak hNav1.5, and, to a lesser extent, hCav1.2 and late hNav1.5). Results are focused on two currents (hERG and peak hNav1.5) as the most data were collected on these currents. Our results provide an indication of “real-world” experiences using different APC platforms and identify controllable sources of experimental variability that should be considered in guiding future best practices with APC approaches. Adhering to consensus standardized experimental conditions and protocols (to reduce experimental variability), along with the use of positive controls (to calibrate results across various platforms and sites) should lead to improved *in vitro*-based estimates of safety margins for important clinical effects of drugs and better early predictions of proarrhythmic risks.

## Materials and methods

The following represent the consensus protocol recommendations. However, actual practices sometimes varied from site to site. A summary of survey results that captures these variations to the extent possible is found in the Results section and Supplementary Table S11.

### Participating sites and platforms

The following 17 sites provided data used in this analysis (in alphabetical order): AstraZeneca, Bayer AG, Bristol-Myers Squibb, B’SYS GmbH, Charles River Laboratories (n = 2), Eurofins, Metrion Biosciences, Nanion Germany (n = 3), Nanion Japan, Nanion USA, Natural and Medical Science Institute at the University of Tübingen, Northwestern University, Roche, and Sophion Bioscience. (To note, numerals in parentheses indicate different sites or protocols linked to facilities).

APC voltage clamp instruments used in this study were as follows: SyncroPatch384PE or 768 (Nanion, Munich, Germany), Patchliner (Nanion), QPatch16 or QPatch48HT (Sophion Bioscience, Ballerup, Denmark), IonWorks Barracuda (Molecular Devices, San Jose, CA, USA), and IonFlux (Fluxion, San Francisco, CA, USA). The different SyncroPatch and QPatch models were considered as being identical and grouped together in the analysis. Participating sites agreed to keep platforms anonymous to encourage vendor participation. Table 1 provides an anonymized list of sites along with platform numbers (defined as the APC instrument and ignoring possible specific nuances of use at any particular site). The use of the same APC platforms at different sites enabled comparisons of site-to-site variability for some platforms. Platform identifiers along with the number of datasets contributed from each platform are as follows: p1, 6; p2, 4; p3, 4; p4, 2; and p5, 1. Sites 8 and 9 intended to participate in this study but did not provide data used in this evaluation.

**Table 1:** Sites, platforms, experimental preparations, and currents analyzed.

Site number	Platform number	Temperature (°C)	hERG current	hNav1.5 peak	hNav1.5 late	hCav1.2
s01	p1	23	CHO	CHO	n.d.	CHO
s02	p2	22–26	HEK293	HEK293	n.d.	CHO
s03	p2	Room	HEK293	n.d.	n.d.	n.d.
s04	p3	Room	CHO	CHO	n.d.	CHO
s05	p4	22–24	HEK293	HEK293	HEK293	CHO
s06	p4	22–24	HEK293	n.d.	n.d.	n.d.
s07	p5	Room	HEK293	HEK293	HEK293	n.d.
s10	p3	Room	HEK293	CHO	n.d.	n.d.
s11	p1	35	HEK293	HEK293	n.d.	CHO
s12	p2	23–26	HEK293	CHO	n.d.	n.d.
s13	p2	35	CHO	CHO	CHO	n.d.
s14	p1	22–27	HEK293	HEK293	HEK293	CHO
s15	p1	22–23	HEK293	CHO	n.d.	n.d.
s16	p3	Room	HEK293	HEK293	n.d.	CHO
s17	p1	Room	n.d.	HEK293	n.d.	n.d.
s18	p1	35	CHO	CHO	n.d.	CHO
s19	p3	Room	CHO	CHO	n.d.	n.d.

p1–p5 represent different automated patch instrument lines. Site s06 used the Milnes protocol for hERG K^+^ current measurements. CHO, Chinese hamster ovary cells; HEK293, human embryonic kidney 293 cells; n.d., not determined.

### Drug and test article solutions

The effects of 12 drugs representing high, intermediate, and low proarrhythmic risk categories as categorized in the CiPA paradigm^8^ were evaluated (Table 2). Chlorpromazine was distributed to US sites only due to shipping restrictions outside the United States. All drugs (sourced from Millipore Sigma USA) were supplied blinded to each test site (with a unique identifier assigned to each vial), prepared in 100% dimethylsulfoxide (DMSO; #D4540, Sigma), protected from light, stored, and shipped frozen (−20 °C).

**Table 2:** Drugs tested.

Number	Compound Name	TdP Risk Category	Stock Sol’n. (mM)	Nominal Conc’s (µM)
1	Bepridil	High	0.3	0.01, 0.03, 0.1, 0.3
2	Chlorpromazine	Intermediate	3	0.1, 0.3, 1.0, 3.0
3	Cisapride	Intermediate	0.3	0.001, 0.01, 0.1, 0.3
4	Diltiazem	Low	100	3, 10, 30, 100
5	Dofetilide	High	0.1	0.001, 0.003, 0.01, 0.1
6	Mexiletine	Low	100	1, 10, 30, 100
7	Ondansetron	Intermediate	10	0.3, 1.0, 3.0, 10
8	Quinidine	High	10	0.1, 0.3, 1.0, 10
9	Ranolazine	Low	100	3, 10, 30, 100
10	Sotalol	High	300	10, 30, 100, 300
11	Terfenadine	Intermediate	0.3	0.01, 0.03, 0.1, 0.3
12	Verapamil	Low	1	0.03, 0.1, 0.3, 1.0

The 12 drugs tested, along with proarrhythmic risk levels, concentrations of stock solutions as distributed to each participating site, and nominal concentrations tested. Drugs were categorized into high, intermediate, and low torsadogenic (TdP) risk based on CiPA evaluations. CiPA, Comprehensive *In Vitro* Proarrhythmia Assay; TdP, Torsades-de-Pointes.

At each test site, test article solutions were prepared fresh daily by diluting stock solutions into DMSO at half-log increments in molarity of the provided solution to create a dilution series of four concentrations. These dilutions were prepared and stored in glass vials and ultimately formulated in assay buffer to attain a final concentration 1/1000th of this serial dilution for use. It was recommended that each test article formulation be sonicated at room temperature (approximately 22 °C) to ensure dissolution. If possible, test article solutions were prepared and stored in glass reservoirs and transferred with glass pipettes. When possible, low-binding plastic ware (e.g., pipette tips to transfer sub-milliliter volumes) was used. Deviations with regard to the recommendations as well as the use of glass were documented as far as possible and are included in the survey results (Supplementary Table S11). All test articles were protected from light. Previous results have shown that ≤ 0.1% DMSO does not affect currents; therefore, each test article formulation contained a maximum of 0.1% DMSO^12^. For a negative control, a vehicle buffer of a maximum of 0.1% DMSO was prepared.

### Solutions

The protocol recommended recording solutions per ionic current (see below) and recognized deviations that may have been necessary to ensure assay stability for the different platforms. The solutions used are listed in Supplementary Table S1.

### Recombinant cell lines

All sites expressed channels in either human embryonic kidney 293 (HEK293) cells or Chinese hamster ovary (CHO) cell lines (see Table 1).

### Voltage clamp protocols

#### hERG current

The onset and steady state block of hERG current was measured using a pulse pattern, repeated every 5 s, consisting of a depolarization to 40 mV amplitude for a 500-ms duration, followed by a ramp (−1.2 V/s) to −80 mV for 100 ms (holding potential −80 mV). Peak tail current was measured during the repolarizing ramp. Leak current was measured after applying a saturating concentration of a blocker such as E-4031 (0.5 µM) at the end of each experiment to completely block hERG current. The remaining current was subtracted from current records.

Di Veroli *et al*.^13^ suggest that the kinetics of hERG current block are as important as the potency of current block for defining the risk of delayed repolarization. The *in silico* modeling group supporting the CiPA initiative has suggested the utility of more detailed kinetic studies of current block for their simulation studies. Thus, a single test site (s06) elicited hERG current with a 10-s depolarizing voltage step from −80 mV to 0 mV every 25 s (Milnes protocol, see Milnes *et al*.^14^). The IC_50_ values derived from this single site using this challenging protocol are also reported.

#### Peak hNav1.5 current

Onset and steady state block of peak hNav1.5 current was measured using a pulse pattern, repeated every 5 s, consisting of a hyperpolarizing pulse to −120 mV for a 200-ms duration, depolarization to −15 mV amplitude for a 40-ms duration, followed by step to 40 mV for 200 ms and finally a 100-ms repolarizing ramp (−1.2 V/s) to a holding potential of −80 mV. Peak current was measured during the depolarizing step to −15 mV. Leak current was measured after applying a saturating concentration of a blocker such as lidocaine (2 mM) at the end of each experiment to completely block hNav1.5 current.

#### Late hNav1.5 current

Late hNav1.5 current was measured using the same voltage protocol as peak hNav1.5 current. However, all external solutions contained 30 nM ATX-II in order to activate late current. Late current was measured at its maximum during the repolarizing ramp.

#### hCav1.2 current

Onset and steady state block of hCav1.2 current was measured using a pulse pattern, repeated every 5–15 s, consisting of a depolarization to 0 mV amplitude for a 40-ms duration, followed by a step to 30 mV for 200 ms and finally a 100-ms ramp (−1.2 V/s) to the holding potential of −80 mV. Peak current was measured during the step to 0 mV. Leak current was measured after applying a saturating concentration of a blocker such as cadmium (200 µM) at the end of each experiment to completely block hCav1.2 current.

### Assay conditions and procedures

Cell harvesting for use on the assay platforms was performed according to optimized procedures established at each test site. General harvesting procedures are not well described or defined and generally are developed in-house by each laboratory.

While most experiments were performed at ambient temperature, two platforms (p1 and p2) conducted experiments at near physiologic temperature (35 °C) at a total of three sites (Table 1).

#### Treatment groups

Concentration-response relationships for each test article were independently investigated, i.e., each cell received only one test article, typically evaluating multiple concentrations in a cumulative fashion (lowest to highest concentrations). However, some platforms tested only one concentration per well. Each concentration response was based on a minimum of four concentrations, with a minimum of three wells used for each concentration. In cases of high variability, more cells per concentration were recorded. Prior to drug addition, cells received an addition of vehicle to measure non-test article-related effects such as addition artifacts. At the end of the experiment, a supramaximal concentration of a known inhibitory reference compound was applied to assess leak current and appropriate responsiveness of the test system. While it was recommended that a separate group of cells (n ≥ 3) be recorded for each experimental day to determine the baseline response to vehicle and to address day-to-day variability, this was not verified.

#### Quality control parameters

Valid whole-cell recordings had to meet the following criteria: (i) membrane resistance (Rm) ≥ 200 M-Ohm as estimated for a single-cell patch clamp experiment, (ii) leak current ≤ 25% peak current, and (iii) baseline current ≥ 0.2 nA. These criteria were applied to all APC platforms.

### Data analysis

The steady state current at each concentration was used to determine the concentration-response relationships. Mean concentration-response data were fitted to a standard sigmoidal four-parameter logistic equation (GraphPad Prism 7; GraphPad Inc., La Jolla, CA) of the form:

$${\rm{Y}}={\rm{B}}{\rm{o}}{\rm{t}}{\rm{t}}{\rm{o}}{\rm{m}}+({\rm{T}}{\rm{o}}{\rm{p}}-{\rm{B}}{\rm{o}}{\rm{t}}{\rm{t}}{\rm{o}}{\rm{m}})/(1+{10}^{\wedge }({{\rm{L}}{\rm{o}}{\rm{g}}{\rm{I}}{\rm{C}}}_{50}-{\rm{X}})\ast {\rm{H}}{\rm{i}}{\rm{l}}{\rm{l}}\,{\rm{S}}{\rm{l}}{\rm{o}}{\rm{p}}{\rm{e}})$$

where Y is the current inhibition (in % of pre-drug control), X is the logarithm of drug concentration, and IC_50_ is the drug concentration producing half-maximal current inhibition. Curve fitting was performed using the following constraints: Top = 100%, Bottom = 0%, Hill Slope <2. No curve fitting was performed in cases with an obvious lack of a concentration-dependent current inhibition and/or small effect size (approximately ≤20%).

### Statistics

IC_50_ values for current block were compared after logarithmic (base 10) transformation to conform data more closely to a normal distribution. Thus, the difference (sum) of two logged values corresponds to the ratio (product) on the original scale. An analysis of variance was conducted separately for each drug to quantify the contributions of between-platform (across sites using different platforms) and within platform (across sites using the same platform) sources to total variability, thus providing a variance component analysis. The factor in this one-way analysis of variance was platform. The residual error thus measures the within-platform variability (i.e., due to technical replicates using a given platform for a given compound). For this pilot study, variance component analysis was only feasible for hERG IC_50_. For the other three currents, data were too scarce to have variance component estimates (i.e., the number of platforms with more than one measurement was less than three). Repeatability coefficients were calculated based on the residual error. To be specific, the repeatability coefficient is defined to be 1.96 × √ 2 × residual error, which measures the typical (i.e., 95% confidence) difference between measurements using the same platform.

Agreement analysis for both within-platform and between-platform comparison was conducted using linear correlation as well as the method of Bland and Altman^15^. These plots compare different methods (in this case platforms) based on plots of averages of paired IC50 values generated by two different methods (plotted as x) vs. the difference between paired values (plotted as y) generated by two different methods. Bias is represented by the fit between the regression line to the points vs. the y = 0 line. Sufficient n sizes were available for a comparison of platforms for drug block of only hERG and hNav1.5 current.

### Data variability questionnaire

After submission of datasets, a questionnaire was designed and distributed to gain a better understanding of the sources of variability observed, with 10 volunteer sites responding. Questions addressed included protocol deviations, solution preparation, composition of materials in contact with the test articles, and electrophysiological procedures.

## Results

### Potency of hERG and peak hNav1.5 current block

Drugs effects were evaluated on five different APC platforms across 17 test sites (Table 1). Sixteen of 17 sites contributed hERG current data, 15 sites contributed peak hNav1.5 current data, 8 sites contributed hCav1.2 current data, and 4 contributed late hNav1.5 current data. Due to the smaller datasets with hCav1.2 and late hNav1.5 current, only results for hERG and peak hNav1.5 are shown in the main body of the paper, with results for the other currents presented in Supplementary Tables S8–S10 and in Fig. S2 (hCav1.2) and S6 and S7 (late hNav1.5). More complete data for all currents are provided in the supplementary materials.

**Figure 1:**
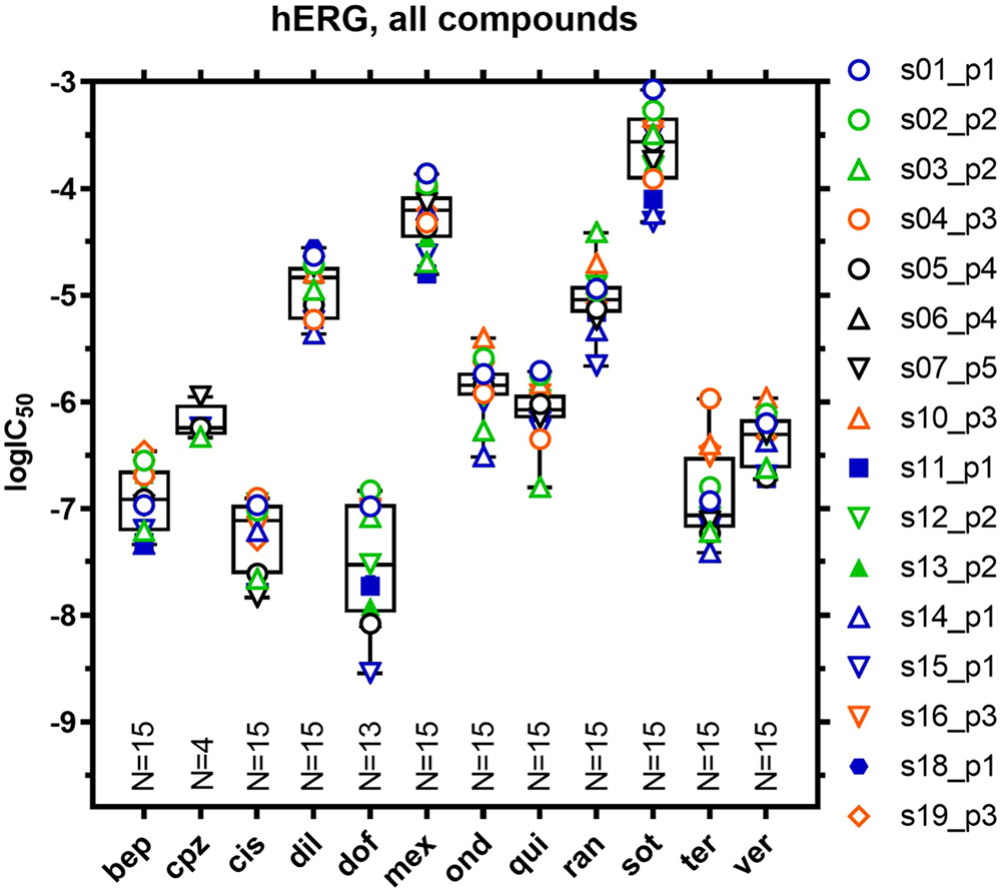
hERG K^+^ current IC_50_ concentrations. Graphical representation of hERG K^+^ current inhibition by the 12 test compounds: spread of individual log-IC_50_ concentrations per compound and per site (compare Supplementary Tables S2–S4) with superimposed box-and-whiskers plot (median, 25/75 percentile, min/max values); the number of data points from automated platforms is indicated at the bottom of each column. Platforms used are color-coded as follows: p1, blue; p2, green; p3, orange; p4, black; and p5, black. Assays (s01–s19) were conducted at room temperature (open symbols) and at 35 °C (filled symbols). bep, bepridil; cpz, chlorpromazine; cis, cisapride; dil, diltiazem; dof, dofetilide; mex, mexiletine; ond, ondansetron; qui, quinidine; ran, ranolazine; sot, sotalol; ter, terfenadine; ver, verapamil.

Figure 1 compares the potency of hERG block (based on IC_50_ values) for all 12 drugs across sites. Overlaid box plots (showing median, 25/75 percentiles, min/max values, and n-sizes) summarize the extent of variability across sites and platforms for each drug. A wide range of values is discernable across compounds from larger datasets (n = 13–15), with 25/75 percentiles ranging from 1.02 log units (10.4-fold) for dofetilide to 0.22 log units (1.7-fold) for quinidine. The concentration-response relationships for all 12 test compounds at respective sites are shown in Supplementary Fig. S1, with tabulated data (including Hill slopes and percentage block at minimum and maximum concentrations) listed in Supplementary Tables S2–S4.

**Figure 2:**
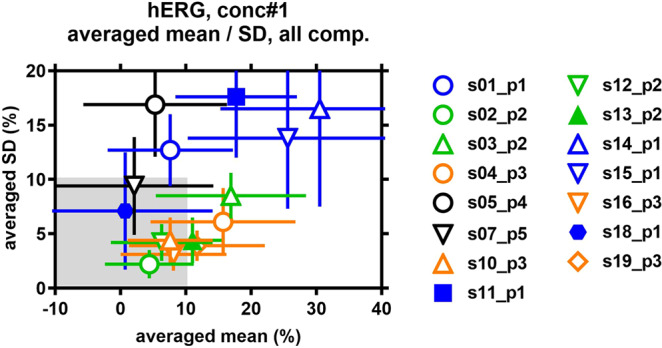
Variability of hERG K^+^ current inhibition at NOEL/LOEL concentrations per site. Test compound concentrations were chosen in such a manner that the lowest concentration for each compound was known/expected to cause no (non-inhibitory, no observable effect level [NOEL]) or only a minor inhibition (lowest observable effect level [LOEL]) of the hERG K^+^ current. Therefore, at these NOEL/LOEL concentrations, current inhibition values are expected to cluster around zero percent. Inhibition mean values (and SD) at the lowest test compound concentration were averaged per site over all 12 test compounds. Platforms used are color-coded as follows: p1, blue; p2, green; p3, orange; p4, black; and p5, black. Automated assays (s01–s19) were conducted at room temperature (open symbols) and at 35 °C (filled symbols).

Each drug was tested at four concentrations chosen such that the lowest concentration should elicit no or minimal block and the highest concentration should elicit nearly full block (unless limited by compound solubility). Figure 2 shows the mean effect size and mean standard deviation for hERG block, averaged at the lowest (i.e. non-inhibitory, no observed effect level [NOEL]) or minimal blocking (lowest observed effect level [LOEL]) concentrations of all 12 test articles per site across the 15 sites. The mean inhibitory effect per site at the lowest concentration ranged from near zero to 31% and the standard deviation ranged from a few percent to slightly less than 20%. Sites using platforms p2 and p3 cluster fairly close together, with mean inhibitory effects between 4 and 17% and mean SD between 2 and 8%, whereas sites using the platform p1 display a greater spread, with mean inhibitory effects between 1 and 31% and mean SD between 7 and 18%. These results suggest inherently greater variability associated with platform p1 (across five sites) compared to p2 and p3 (each across four sites).

**Figure 3:**
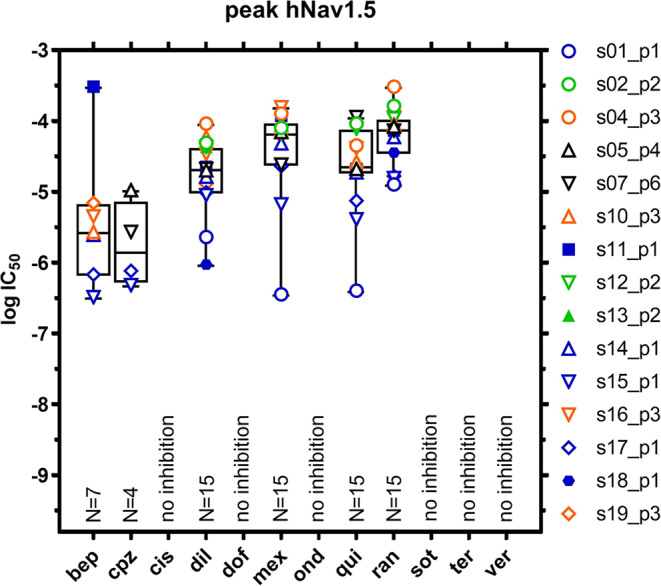
Peak hNav1.5 Na^+^ current IC_50_ concentrations per compound. Graphical representation of peak hNav1.5 Na^+^ current inhibition by the 12 test compounds: spread of individual log-IC_50_ concentrations per compound and per site (compare Supplementary Tables S5–S7) with superimposed box-and-whiskers plot (median, 25/75 percentile, min/max values); the number of data points from automated platforms is indicated at the bottom of each column. Platforms used are color-coded as follows: p1, blue; p2, green; p3, orange; p4, black; and p5, black. Automated assays (s01–s19) were conducted at room temperature (open symbols) and at 35 °C (filled symbols). No inhibition defined as no obvious concentration-dependent current block and/or no curve fitting performed. bep, bepridil; cpz, chlorpromazine; cis, cisapride; dil, diltiazem; dof, dofetilide; mex, mexiletine; ond, ondansetron; qui, quinidine; ran, ranolazine; sot, sotalol; ter, terfenadine; ver, verapamil.

Figure 3 shows IC_50_ values for block of peak hNav1.5 obtained for all 6 of 12 drugs eliciting current block. Overlaid summaries describing the variability of each dataset are provided using box plots (median, 25/75 percentile, min/max values, and n-sizes [as described for Fig. 1]). As was shown for hERG current, a wide range of values is discernable across sites and platforms. For example, the range of percentile values (25/75) for diltiazem, mexiletine, quinidine, and ranolazine (for which 15 values were available) were 32.9, 71.5, 59, and 48.3 μM, respectively.

**Figure 4:**
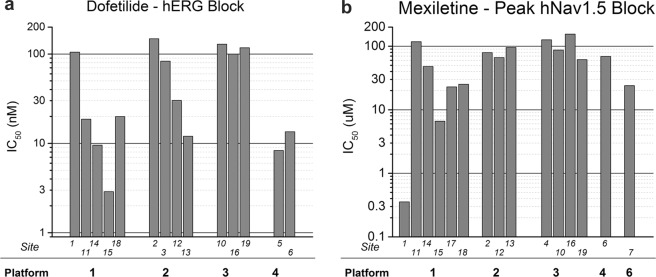
Platform variability for hERG and peak Nav1.5 current block. A comparison of platform variability for dofetilide block of hERG (**a**) and mexiletine block of hNav1.5 current (**b**). Despite limited data, minimal variability is evident for block of both currents with platform 3, while prominent differences in variability for block of hERG vs. peak hNav1.5 current for platform 2 are observed. Note the logarithmic scale used to illustrate the wide range of values obtained within and between platforms.

Figure 4 compares IC_50_ values for block of hERG and peak hNav1.5 current obtained with the same automated patch platforms used across different sites. Figure 4a compares IC_50_ values for dofetilide block of hERG current with four automated platforms across a total of 14 sites (with the number of replicate machines ranging from 5 [platform 1] to 2 [platform 4]). Some platform/site combinations show greater variability than others for the same drug and current (e.g., dofetilide responses for platform 1 [5 sites] vs. platform 3 [3 sites]). Figure 4b compares IC_50_ values for mexiletine block of peak hNav1.5 current with five automated platforms across a total of 15 sites (number of replicate machines ranging from 1 to 6). Platform 1 again appears to show greater variability (compare results from six sites using platform 1 vs. 5 sites using platform 3). The data (while limited) also suggest that differences in the IC_50_ variability may not be intrinsic to the platform: for example, platform 2 shows more variability for hERG current block with dofetilide than for mexiletine block of peak hNav1.5 current (compare second set of columns).

### Variance component analysis for hERG and peak hNav1.5 current block

**Figure 5:**
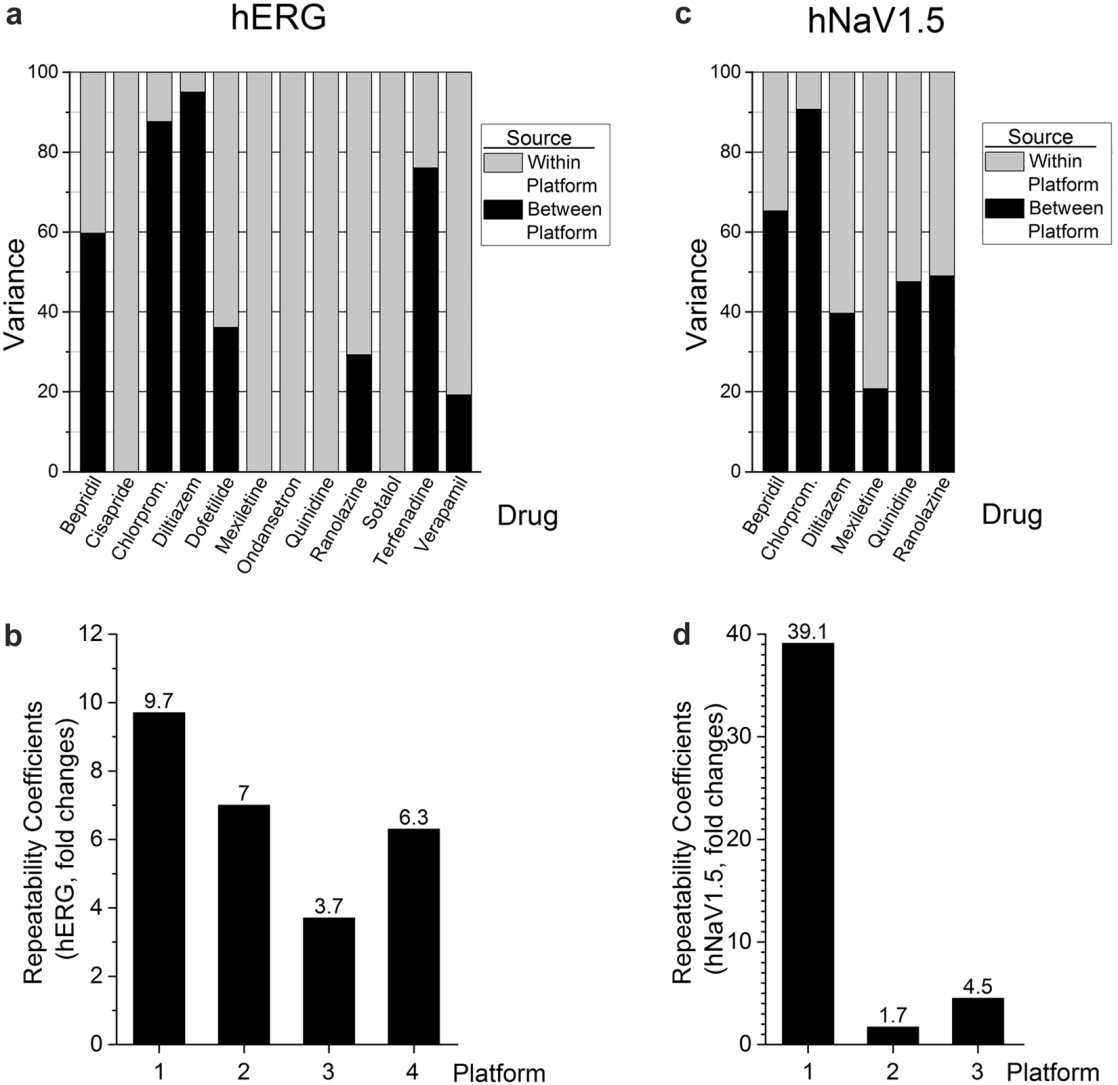
A comparison of between- and within-platform variance and repeatability coefficients for hERG and hNav1.5 currents. (**a**) For hERG, within-platform variance predominated for 6 of 12 drugs (cisapride, mexiletine, ondansetron, quinidine, sotalol, and verapamil). (**c**) For hNav1.5, within-platform variance predominated for 2 of 6 drugs (diltiazem and mexiletine). (**b**,**d**) Repeatability coefficients for hERG and hNav1.5 current block differed widely across automated patch platforms from which sufficient data were available.

Variance component analysis was conducted to quantify the contributions attributed to automated patch platform and site to the total variability for hERG current block. For this analysis, between-platform variability was taken into account and factored into the statistical model. The residual error thus measures the within-platform variability (i.e., due to replicates on a given platform for a given compound). Figure 5a compares between- and within-platform variance for block of hERG current. Within-platform variance predominated for 6 of 12 drugs (cisapride, mexiletine, ondansetron, quinidine, sotalol, and verapamil), while between-platform variance dominated for chlorpromazine, diltiazem, and terfenadine. These results demonstrate that no one platform contributed overall greater intrinsic variability for hERG current block. Figure 5c also illustrates large differences in between- vs. within-platform variance for the six drugs blocking hNav1.5.

Repeatability coefficients are useful for assessing overall within-platform agreement across sites (thus informing about precision of the measures). Repeatability coefficients for hERG IC_50_ values across four automated platforms for all drugs with sufficient data are shown in Fig. 5b. Lower coefficients indicate better agreement across sites using the same platform. For example, a repeatability coefficient of 3.7 for platform p3 means that typical measurements of hERG IC_50_ done with p3 were within 3.7-fold. Greater repeatability coefficients are evident for platforms 1, 2, and 4, with platform 1 providing a coefficient of 9.7 divergent IC_50_ measures within nearly 10-fold of each other.

Repeatability coefficients for block of peak hNav1.5 were calculated only for platforms p1, p2, and p3, as multiple measurements for only some compounds were performed (Fig. 5d). The repeatability coefficient for platform 1 was very high (39.1, representing inconsistent IC_50_ determinations compared to platforms 2 and 3 (values of 1.7 and 4.5, signifying values within 1.7- and 4.5-fold of each other).

### Agreement analysis

**Figure 6:**
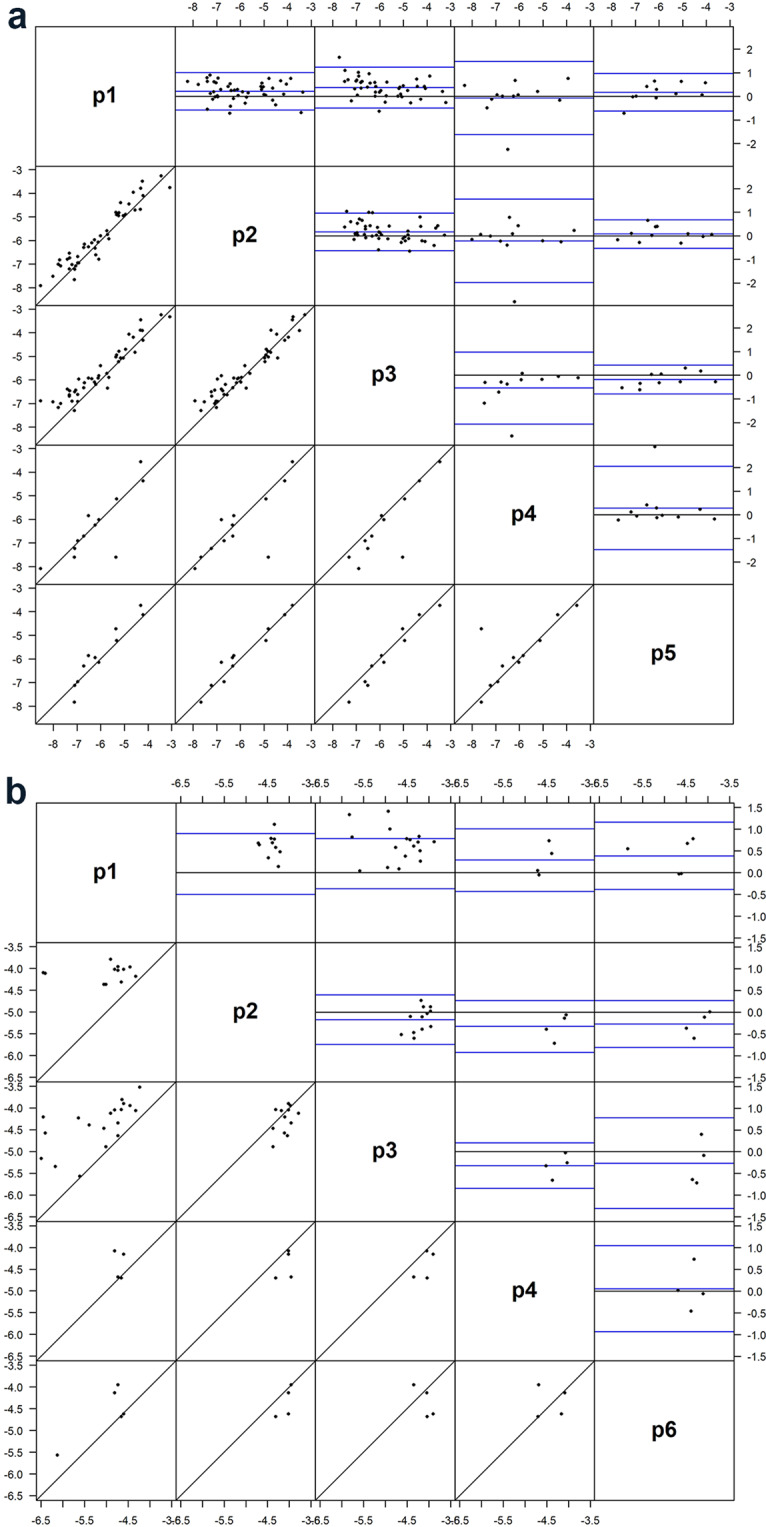
Between-platform agreement analysis for hERG IC_50_ (**a**) and hNav1.5 IC_50_ values (**b**). The upper right graphs display Bland-Altman plots describing between-platform agreement, with the x-axis representing mean of two determinations for each drug, and y-axis representing the difference between the two determinations (both in molar units). The lower left graphs display correlations for drugs tested across the two platforms being compared. The axes represent nominal drug test concentrations (in log units). Each dot represents one drug in the comparison datasets. p1–p6 represent five different platform with sufficient data across sites to enable comparisons.

Agreement analysis (typically used to compare measures obtained from two different methods or approaches) for hERG and peak hNav1.5 current is shown in Fig. 6a,b, respectively. Within each panel, the subplots in the lower left of the matrix are scatter plots to measure the correlation between two platforms. If two platforms have perfect agreement in their measures, then points on the scatter plots would be close to and symmetric about the diagonal line. There appears to be more agreement for hERG current (Fig. 6a) than for peak hNav1.5 block (Fig. 6b), although the number of observations for peak hNav1.5 is less.

While useful in exploring data, scatter plots and correlations are deficient for defining agreement between methods compared to Bland-Altman plots^16^. Bland-Altman plots display differences between any two platforms (A-B) on the ordinate vs. their mean value [(A + B)/2] on the abscissa. If two platforms have perfect agreement in their measurements, then Bland-Altman plots display the following: (1) narrow limits of agreement (LsA; defined as mean difference ± 2 × standard deviation corresponding to 95% coverage) and (2) mean differences approximating zero. Within each subplot are three blue lines; the highest and lowest blue lines delineate LsA values. The wider this range, the less agreement the two platforms share in the measurement. The middle blue line represents the mean difference between the two platforms. The distance from the middle blue line to the zero (gray) line measures the average relationship between the two 2 platforms (i.e. whether platform A overall has higher or lower measurements than platform B). The acceptance criterion of LsA within (−0.48, 0.48) has been recommended^17^ for high-throughput screening (HTS) assays, corresponding to 3-fold differences on the raw scale [i.e., (0.33, 3)].

By the above criterion, no two platforms in this dataset passed the agreement assessment using hERG IC_50_ values obtained from five platforms. When both the mean ratio (reflecting bias) and standard deviation (reflecting variability) are taken into consideration, the best agreement in hERG IC_50_ (Fig. 6a) was observed between p2 and p5, with a mean ratio of 0.78 and LsA of (0.14, 4.44) on the original scale (Fig. 6a). In other words, the average IC_50_ with p5 was 78% of that with p2, and the typical differences are between 0.14- and 4.1-fold.

Figure 6b shows the agreement between four platforms for hNav1.5 IC_50_ values. Because of the scarcity of data across platforms, only comparisons between p1 and p3 were meaningful for this dataset. The agreement between p1 and p3 had a mean ratio of 2.05 and LsA of (0.05, 90.4) on the original scale. In other words, the average IC_50_ with p1 was almost twice as high as that with p3, and the typical differences are between 0.05- and 90.4-fold. Clearly this agreement was weaker than for hERG IC_50_ and did not pass the 3-fold criterion.

Overall the between-platform agreement was low and did not meet the typical 3-fold acceptance criterion for HTS assays. It is worth noting that variability seemed to vary from compound to compound. Further, the compound and/or replicate distribution lacks consistency across platforms and hence the applicability of the agreement analysis could be limited. Such variability may be related to differences in the characteristics of different platforms (384- vs. 16-well plates) that affect drug concentrations via well size, and the use of multiple vs. single holes that may affect data quality, and are consistent with different expectations of lower vs. higher throughput screening instruments (e.g., QPatch vs. Patchliner vs. Nanion and Barracuda devices [the latter designed for higher throughput screening applications]).

### Study protocol deviations and data variability questionnaire

Despite efforts to standardize experimental protocols and assay conditions, wide differences in block potency were noted for some drugs and platforms during initial reviews of submitted datasets. To qualitatively explore possible sources of variability arising from unexpected differences in experimental conduct, a survey inquiring about specific assay conditions was subsequently distributed to participating sites. Details provided from the 10 responding sites are summarized in Supplementary Table S11.

All sites used the consensus voltage clamp protocols, although one site applied pulses at a faster frequency (0.2 Hz vs. 0.1 Hz). Eight of nine sites stored test articles in glass (one using plastic). Four of the sites added only one addition of each concentration to the assay well. Five sites used buffers slightly different from those recommended in the standardized protocol and also applied ascending multiple concentrations to each cell, which may have resulted in exposure times that were too short. Three sites measured responses to each concentration after 3 minutes or less exposure time. One site used an alternate voltage protocol and one site did not vortex, sonicate, or heat the solutions to promote drug dissolution. One site stored formulations in plastic and used plastic compound plates that may have bound up lipophilic drugs. Such different protocol nuances likely contributed to the data variability. Finally, no site was able to successfully use the Milnes dynamic block voltage clamp protocol to assess the kinetics of hERG block during the study, likely due to technical difficulties in achieving longer stable recordings (typically ≥40 minutes) required to generate each one-cell/one concentration datapoint.

## Discussion

### Summary of findings

To our knowledge, this multi-platform/multi-site study provides the largest comparison of results from 5 APC platforms across 17 sites evaluating drug effects on 4 human cardiac currents in heterologous expression systems. We will focus on practical lessons learned from assessing blocking potency of 12 blinded drugs (representing high, intermediate, and low proarrhythmic risk categories) on block of hERG and peak hNav1.5 current as these currents were evaluated by a majority of sites. Prominent differences in IC_50_ values for block of both currents across multiple platforms and sites are apparent. This variability is not unique to any particular platform or site and varied with the drug and current studied. For example, for automated platforms the IC_50_ values (25/75 percentiles) for hERG block with dofetilide ranged from 12 to 103 nM (10.4-fold range, 14 sites, 4 platforms) and from 0.77 to 1.08 μM (1.4-fold range, 16 sites, 5 platforms) with quinidine (Fig. 1).

The contributions of between- and within-platform variance for block of hERG current varied greatly across the 12 drugs tested, ranging from >90% between-platform variance for chlorpromazine and diltiazem to 100% within-platform variance for cisapride, mexiletine, ondansetron, quinidine, and sotalol (Fig. 5a). Between-platform variance for all 12 drugs for hERG and peak hNav1.5 is also shown in Bland-Altman plots (Fig. 6, upper right matrices). Perfect agreement would require all points to lie on the zero line but all comparisons displayed considerable differences. Platforms p2 and p5 had the best agreement with a mean ratio of 0.76, indicating that on average IC_50_ values from p5 are 76% of those of p2.

### A comment on the Milnes dynamic block voltage clamp protocol for assessing kinetics of hERG current block

The Milnes clamp protocol^14^ provides detailed descriptions of the kinetics of block of I_Kr_ that is needed for the computational assessment of proarrhythmic risk in the CiPA paradigm. This protocol consists of a set of two 25-s-long pulse protocols, with one protocol (“Pulse”) containing a prolonged 10-s-long depolarizing test pulse (0 mV) followed by a 14-s-long return pulse to −80 mV, and a second protocol “No-Pulse” consisting of the same pulse structure without the depolarizing test pulse^18^. Stability of hERG current with this protocol (defined as <10% difference in current amplitude across 20 clamp protocols) is demonstrated by applying 10 repeats of the “Pulse” protocol followed by 10 repeats of the “No-pulse” protocol and comparing peak and steady state hERG current. This protocol is monitored during drug administration (to confirm equilibration of drug effects) with a cell being exposed to only one drug concentration (at 37 °C). While this protocol has been successfully implemented on an HTS platform^19^, this rigorous experimental approach presents formidable challenges for even the most robust cellular recordings using automated patch platforms. One site performed a modified version of Milnes that consisted of a shortened stability period, but the submitted data were not acceptable for kinetic data. The amount of time a patch needed to be maintained to achieve an acceptable level of stability was very difficult to achieve. The Milnes data was only useful to calculate the IC_50_ value for hERG block, no other participating site was successful in implementing the Milnes dynamic block voltage clamp protocol for kinetic data. The lack of success in applying this challenging voltage clamp protocol makes it clear that some protocols do not transfer easily from manual to APC platforms. The implementation of the Milnes protocol might be best suited for manual patch techniques later in the drug discovery process conducted for risk assessments requiring more extensive characterization on a few candidate compounds (or for regulatory submissions). Beattie *et al*.^20^ and Lei *et al*.^21,22^ recently published voltage protocols more amenable to APC approaches to characterizing the kinetics of hERG block that might prove as suitable alternatives to the Milnes voltage clamp protocol.

### Importance of reducing variability in setting safety margins and application to the CiPA paradigm

The widely recognized use of the potency of hERG current block as a surrogate marker of drug-induced QT prolongation provides the basis for its inclusion in the ICH S7B Guidance, “Nonclinical Evaluation of the Potential for Delayed Ventricular Repolarization (QT Interval Prolongation) by Human Pharmaceuticals,”^23^ and its routine use in setting safety margins for QT prolongation (with a threshold typically 30- to 45-fold based on maximal clinical protein-unbound drug exposures^24–27^). Further, the accurate assessment of drug-induced potency of block of the four human cardiac currents evaluated in this study (hERG, hCav1.2, peak hNav1.5, late hNav1.5) is essential to inform *in silico* reconstructions used to define proarrhythmic risk in the evolving CiPA paradigm^9,10^. Thus, understanding (and minimizing) the variability inherent in IC_50_ estimations is essential to predicting and understanding the cardiac safety evaluations of evolving drug candidates.

Few studies have described variability regarding potency of block of cardiac currents with APC platforms, although not across the wide number of sites, platforms, and currents as in the present study. Perhaps the most detailed study of variability with an automated patch platform was published by Elkins *et al*.^11^ who quantified the variability of Molecular Devices IonWorks Quattro platform by evaluating potency of hERG block from hundreds of experiments for multiple drugs across two pharmaceutical companies over a few years period. We compared the variability of hERG block for three drugs tested in both the present study and the Elkins study (quinidine, verapamil, and cisapride) based on differences in the 10–90 percentiles (recalculated from the distribution of pIC50 values from present study) and the 10–90% probability values from the pIC50 logistic distribution probability plots described by Elkins [their Fig. S2]). The dispersion of pIC50 values for quinidine, verapamil, and cisapride were somewhat comparable across both studies and ranged between ½ to near 1 log unit (0.52 [present study] vs 0.40 [Elkins study] for quinidine, 0.60 vs 0.73 for verapamil, and 0.80 vs. 0.45 for cisapride). For these drugs the potency of hERG block in the present study was somewhat greater compared with the Elkins study but with less than a 10-fold difference (IC50 values of 0.85 vs. 2.37 for quinidine, 0.49 vs. 0.68 for verapamil, and 0.08 vs. 0.39 for cisapride [in µM, present study vs. Elkins study]).

It is also informative to compare variability of hERG block we describe with APC systems with previous published results from manual patch clamp experiments. Some limited estimates can be gleaned from literature sources. IC_50_ values for hERG block of nine 9 blinded compounds recorded at physiologic temperatures from two different manual patch clamp laboratories were up to 3-fold different (with one compound [pimozide] being 44-fold different^28^). Kirsch *et al*.^29^ quantified the variability of the manual patch clamp results from one site/platform by comparing inhibition of hERG current block (using HEK293 cells at 22 °C) with either 60 nM terfenadine or 90 nM cisapride multiple times over periods of 20 and 18 months, respectively. Terfenadine (n = 459 cells) and cisapride (n = 83 cells) elicited 81.7 ± 7.0% and 83.4 ± 7.7% (mean ± SD) hERG current inhibition, with 96–97% of data within 2 standard deviations of the mean. Using the 2 standard deviation limit, it is possible to calculate a range of IC_50_ values of 3–29 nM for terfenadine and 42–1093 nM for cisapride. Crumb *et al*.^30^ also evaluated the hERG blocking potency of nine drugs also tested in the present study using a manual patch clamp; and an action potential waveform (instead of the step-ramp voltage protocol used in this study) with recordings obtained at 36 ± 1 °C. Our APC IC_50_ values for chlorpromazine, verapamil, bepridil diltiazem, quinidine, sotalol, and cisapride were 0.4- to 1-, 0.4- to 2.2-, 0.3- to 2.3-, 0.7- to 4.3-, 0.5- to 5.7-, 0.6- to 9.7-, and 1.3- to 10.6-fold different (less) than the IC_50_ values obtained by Crumb *et al*.^30^, respectively. The largest differences were seen for terfenadine and dofetilide; APC derived IC_50_ values were 3.1- to 58-fold and 1.8- to 90.2-fold different from the Crumb *et al*.^30^ values for terfenadine and dofetilide, respectively.

### Best practices to reduce experimental sources of variability

Experimental variability can be divided into three components: namely, systematic variance due to true differences in the signals being compared, systematic error arising from confounding factors, and random error. The variability in IC_50_ values reported in this large study (as well as prior studies) leads one to question the minimum amount of variability that can be achieved using either manual patch or APC approaches. In this study, a standardized protocol was provided to all sites along with centrally supplied blinded drugs to reduce one source of systematic errors. However, deviations in experimental conditions and the execution of study protocols were noted. This was not entirely unexpected as volunteer sites had prior experience in developing and optimizing similar assays for the different ionic currents with their instruments. It is informative to discuss experimental sources of systematic errors that likely contributed to the observed variability, with an eye toward defining “best practices” for defining potency of drug block of human cardiac currents.

#### Ensuring adequate equilibration time with cells

Equilibration times with cells were variable across sites, with three sites recording responses after 3 minutes or less. Such exposure times are likely too short to allow for equilibration of drug effects for some compounds. Evidence for this is provided from results for hERG block with terfenadine and dofetilide: IC_50_ values for block obtained from platform 1 (7-min exposure) were 7× and 4× more potent than for platform 3 (4-min exposure). Potency of block for low-permeability drugs (vs. higher permeability drugs) would likely be underestimated to a greater extent with inadequate equilibration times.

#### Variability in drug forms and concentrations in stock solutions

Another potential source of variability resides in the physicochemical properties of the drugs themselves. Shoitchet and colleagues^31,32^ have shown that some drugs form aggregates influencing drug efficacy (and apparent potency) in high throughput experimental conditions. They showed that certain colloidal drug aggregates cause false positive inhibition of membrane bound effectors such as G protein-coupled receptors. To our knowledge, the effects of drug aggregation on ion current block has not been studied, but at least one of the CiPA compounds (vandetanib) closely resembles another kinase inhibitor that aggregates (B. Shoitchet, personal communication). A prediction routine available free online (http://advisor.bkslab.org/) can be used to estimate the aggregation susceptibility of compounds. Kolliphor EL (a nonionic surfactant typically used to improve bioavailability of poorly water-soluble drugs and used by two sites in this study) could lead to an improved solubility of problematic compounds and more potent IC_50_ values.

Significantly reduced drug exposures may result from improper preparation of stock solutions, instability, or degradation of drug in concentrated stock or media solutions. Neat DMSO is highly hygroscopic and can absorb up to 25% atmospheric humidity within a couple of days^33^. Repeated freeze-thaw cycles of stock solutions can alter the solubilized fraction of the most lipophilic compounds or ionizes a fraction of the most acidic or alkaline compounds. This can influence replicates collected over several days when computing IC_50_ values. Physical methods such as in-well sonication have been proposed to maintain concentrated stocks in solution^34^, although this may not be possible with higher capacity plates. Finally, the availability of resuspension routines in the drug application protocols should improve homogeneity of the applied drug solution.

Compound dilutions prepared in micro-titer plates were likely applied to the cells after variable times. The variable delays between compound preparation and cell application provide greater chance for micro-precipitation of poorly soluble drugs in aqueous dilution. One site stored stock solutions in plastic plates and used plastic compound plates when delivering drugs to test chambers, which may have resulted in non-specific binding of more lipophilic drugs prior to delivery. Strategies to improve kinetic solubility in buffers supplemented with excipients include addition of cyclodextrins, proteins (e.g., BSA) or emulsifiers (e.g., castor oil or sorbitan derivatives). These compounds display variable effects on hERG currents^12,35^ and should be characterized in the assay systems used. Surfactants such as the poly(ethylene oxide)–poly(propylene oxide) block copolymer Pluronic F-68 can be used up to 1% v/v in APC experiments on QPatch platforms without affecting hERG current amplitude^36^.

#### Low drug exposures in recording chambers

Another source of variability may arise from loss of drug during delivery and adhesion to chamber surfaces (an effect expected to be drug- and plate-dependent). Some platforms used disposable plastic tips while others used absorption-resistant coated metal tips to deliver drug formulations. Non-specific binding could be a major concern in well-based systems due to higher surface-to-volume ratios and the inability to replenish drugs lost to fluidics and well surfaces (an issue less relevant in larger scale flow-through systems). It is possible that substantial drug adsorption to the fluidics and recording chamber may occur, especially with highly hydrophobic drugs such as terfenadine (with a high cLogP value of 7.1). In such circumstances, the use of single additions of drugs to assay wells may lead to lower exposures and potency values due to non-specific binding. This effect may be minimized by employing multiple addition protocols which may allow for saturation of non-specific binding and adsorption. Seven of ten questionnaire respondents did not perform multiple additions. Using the IonWorks Quattro HT platform, Bridgland-Taylor *et al*.^37^ reported that the potency of drug block decreased with increased cell density in recording wells. Although this platform was not used in this study, one could assume similar problems could exist due to cellular uptake of highly lipophilic compounds.

While likely useful, it is difficult (and expensive) to measure drug concentrations in recording chambers to control this source of experimental variability. Regarding this recommendation, a dose-solution analysis for a hERG current block study using manual patch techniques and flow-through test chambers concluded that drug concentration measurements did not alter hERG potency estimation for the majority of compounds tested (confirming nominal concentrations within 3-fold), and did not clarify or improve hERG potency estimated for poorly soluble agents or agents with high cLogP and pKa values^38^. To our knowledge, similar studies using solutions sampled from static APC wells have not been reported.

#### Effect of variability of expressed vs. background currents

Differences in the ratio of overexpressed current of interest (e.g., hERG) vs. endogenous background currents may lead to underestimates of blocking potency if background currents are not considered. For example, some authors prefer CHO cells over HEK cells as a hERG expression system based on generally lower background endogenous currents^20^. These differences may be corrected based on background currents measured at the end of each experiment in the presence of complete hERG block (e.g., with a maximal blocking concentration of a specific hERG blocking agent such as E-4031).

Also, the use of a leak subtraction protocol may be useful for removing background currents. This would reduce the risk of drug contamination and spurious inhibitory effects on other cells during the same experiment or in following plates due to the addition of a maximal concentration of blocking drugs applied at the end of experiments (“end blockers”). If maximal end blockers are used, they should ideally be used in conjunction with subsequent aggressive “extra” pipette washes but that may reduce assay efficiency. Pipette tip-based systems may be less prone to this artifact, but they also require the use of plastic in the final drug delivery workflow, while metal- and glass-coated pipette-based systems do not.

#### Differences in intracellular buffer composition

The components of the recording solutions sometimes differed between sites (Supplementary Table S1). For example, the usage of perforating agents to achieve perforated whole-cell recordings^39^ was used in a subset of experiments, and this could prevent current rundown due to intracellular dialysis. In contrast, potentially higher series resistances (R_series_) in perforated whole-cell (compared to standard whole-cell configurations) can limit bandwidth and confound accurate measurement of rapid ionic currents such as hNav1.5 peak currents in the absence of adequate series resistance compensation^40^. Finally, although the mechanism is uncertain, inclusion of fluoride has long been known to improve patch clamp sealing and stabilizes the cell membrane resulting in longer, more stable patch clamp recordings^41,42^. Fluoride was used in about 65% of the listed solutions (Supplementary Table S1) which may affect recordings compared to those obtained in the absence of fluoride ions.

#### Recording temperature and correcting for current stability/rundown

Only two platforms (used in 3 of 17 test assay results) obtained recordings at near physiologic temperatures (35 °C), with experiments on other platforms conducted at either unspecified (room) temperature or a range of temperatures (Table 1). It is well known that block of hERG with some drugs is temperature dependent^29,43,44^, and this may differentially affect IC_50_ determinations. Little is known regarding the temperature sensitivity of drug block of other cardiac currents. It should also be noted that it is very difficult to adequately control hNav1.5 current at physiologic temperatures due to the rapid kinetics and larger current densities. Wang *et al*.^45^ showed that stabilizing instrument temperature using a portable A/C unit improved current stability and reduced current rundown for hERG, hNav1.5, and hIKs, improving data quality. Correction for rundown (which is likely temperature dependent) was variable across sites, could have contributed to within- or between-platform variability (see Supplementary Table S11), and may be evaluated based on subtraction of vehicle-control results on the same plate.

#### Ability of best practices to reduce variability of blocking potency using APC approaches

Unfortunately, we cannot quantify either the contribution of the different experimental sources of systematic errors to overall variability or the extent to which best practices can reduce variability. Some sources may be platform and drug specific (e.g., temperature stability, differences in the loss of poorly soluble compounds through different solution handling and delivery systems, as well as maintaining the stability of biological expression systems used). Other likely sources of experimental variability “upstream” from the recording sessions are related to the physicochemical properties (poorly soluble compounds may precipitate out of solution) and stability of test compounds (was fresh compound used each time) as well as the procedures used to dispense and prepare the solvated compound plates from which drug is drawn. These are more generalized best practices that need to be considered in addition to the patch-clamp-specific best practices. Some sources of variability will favor underestimates of block potency (e.g., sub-nominal exposures in test chambers and less than adequate equilibration periods). Finally, it should be recognized that the expectation of HTS screening studies with APC’s may be to identify “hits” or to rank-order related compounds based on potency. In contrast, for safety studies later in drug discovery and development (including *in silico* experiments for regulatory safety assessments as in the CiPA paradigm) great care needs to be taken to provide reliable and consistent findings using defined protocols to enable confident translation of non-clinical findings to exposure-based *in vivo* studies and (ultimately) clinical findings.

## Conclusions

Results from this large multi-site study provide estimates of the variability associated with IC_50_ values characterizing the blocking potency of 12 drugs on 4 prominent human cardiac currents using suggested experimental protocols across 5 automated patch platforms and 17 sites. The amounts (and sources) of variability differed prominently with drugs, ionic currents, and platforms. Various suggestions for optimizing experimental protocols and reducing experimental variability and bias were discussed, with the goal of guiding the development of best practices using APC technologies for early screening (to avoid cardiac safety liabilities) and subsequent characterization of the risk of delayed repolarization and proarrhythmia (to guide clinical studies and regulatory submissions). Better specification and execution of consensus experimental protocols is necessary to reduce systematic error contributing unnecessary variability.

The use of consensus positive controls and analysis of drug concentrations tested is suggested to enable robust translation across platforms and for translation to *in vivo* animal-based studies and clinical trials. The variability associated with APC datasets must be considered when (a) defining safety margins related to delayed repolarization for clinical drug candidates (ensuring that the best compounds move into clinical development) and (b) when informing on proarrhythmic risks of drugs using human based *in silico* reconstructions of electrophysiological activity to mechanistically define proarrhythmic risk within the CiPA initiative^9,10,46^.

## Data Availability

Data used in the analyses in this paper are available in the public repository at this link: https://osf.io/muhck/.

## Supporting Information

Supporting Information.
Supporting Information2.
Supporting Information3.
Supporting Information4.
Supporting Information5.
Supporting Information6.
Supporting Information7.
Supporting Information8.
Supporting Information9.
Supporting Information10.

## References

[1] Farre C, Fertig N (2012). HTS techniques for patch clamp-based ion channel screening - advances and economy. Expert Opin Drug Discov.

[2] Danker T, Moller C (2014). Early identification of hERG liability in drug discovery programs by automated patch clamp. Front Pharmacol.

[3] Obergrussberger A (2015). Novel screening techniques for ion channel targeting drugs. Channels (Austin).

[4] Obergrussberger A (2018). An update on the advancing high-throughput screening techniques for patch clamp-based ion channel screens: implications for drug discovery. Expert Opin Drug Discov.

[5] Bell DC, Dallas ML (2018). Using automated patch clamp electrophysiology platforms in pain-related ion channel research: insights from industry and academia. Br J Pharmacol.

[6] Roden DM (2016). Predicting drug-induced QT prolongation and torsades de pointes. J Physiol.

[7] Sager PT, Gintant G, Turner JR, Pettit S, Stockbridge N (2014). Rechanneling the cardiac proarrhythmia safety paradigm: a meeting report from the Cardiac Safety Research Consortium. Am Heart J.

[8] Gintant G, Sager PT, Stockbridge N (2016). Evolution of strategies to improve preclinical cardiac safety testing. Nat Rev Drug Discov.

[9] Dutta S (2017). Optimization of an in silico cardiac cell model for proarrhythmia risk assessment. Front Physiol.

[10] Li Z (2017). Improving the in silico assessment of proarrhythmia risk by combining hERG (human ether-a-go-go-related gene) channel-drug binding kinetics and multichannel pharmacology. Circ Arrhythm Electrophysiol.

[11] Elkins RC (2013). Variability in high-throughput ion-channel screening data and consequences for cardiac safety assessment. J Pharmacol Toxicol Methods.

[12] Himmel HM (2007). Suitability of commonly used excipients for electrophysiological *in-vitro* safety pharmacology assessment of effects on hERG potassium current and on rabbit Purkinje fiber action potential. J Pharmacol Toxicol Methods.

[13] Di Veroli GY, Davies MR, Zhang H, Abi-Gerges N, Boyett MR (2014). hERG inhibitors with similar potency but different binding kinetics do not pose the same proarrhythmic risk: implications for drug safety assessment. J Cardiovasc Electrophysiol.

[14] Milnes JT, Witchel HJ, Leaney JL, Leishman DJ, Hancox JC (2010). Investigating dynamic protocol-dependence of hERG potassium channel inhibition at 37 degrees C: Cisapride versus dofetilide. J Pharmacol Toxicol Methods.

[15] Altman DG, Bland JM (1983). Measurement in medicine: the analysis of method comparison studies. J Royal Stat Soc Ser D.

[16] Bland JM, Altman DG (1986). Statistical methods for assessing agreement between two methods of clinical measurement. Lancet.

[17] Iversen, P. W. *et al*. In Assay Guidance Manual (eds G.S. Sittampalam *et al*.) (Eli Lilly & Company and the National Center for Advancing Translational *Sciences*, 2012). 22553861

[18] US Food and Drug Administration. *FDA CiPA protocol: recommended voltage protocols to study drug-cardiac ion channel interactions using recombinant cell lines*, http://cipaproject.org/wp-content/uploads/sites/24/2018/06/CiPA-protocol-100918.pdf (2018).

[19] Bot C (2017). An “all inclusive“ package for cardiac safety: the six big on one automated patch clamp chip. J Pharmacol Toxicol Methods.

[20] Beattie KA (2018). Sinusoidal voltage protocols for rapid characterisation of ion channel kinetics. J Physiol.

[21] Lei, C. L. *et al*. Rapid characterization of hERG channel kinetics I: using an automated high-throughput system. *Biophys J*, 10.1016/j.bpj.2019.07.029 (2019). 10.1016/j.bpj.2019.07.029 PMC6990155 31447109

[22] Lei, C. L. *et al*. Rapid characterization of hERG channel kinetics II: temperature dependence. *Biophys J*, 10.1016/j.bpj.2019.07.030 (2019). 10.1016/j.bpj.2019.07.030 PMC6990152 31451180

[23] International Conference on Harmonisation of Technical Requirements for Registration of Pharmaceuticals for Human Use. ICH S7B. Note for Guidance on the Nonclinical Evaluation of the Potential for Delayed Ventricular Repolarization (QT Interval Prolongation) by Human *Pharmaceuticals (CHMP/ICH/423/02)*. (ICH, 2005). 16237859

[24] Redfern WS (2003). Relationships between preclinical cardiac electrophysiology, clinical QT interval prolongation and torsade de pointes for a broad range of drugs: evidence for a provisional safety margin in drug development. Cardiovasc Res.

[25] Wallis RM (2010). Integrated risk assessment and predictive value to humans of non-clinical repolarization assays. Br J Pharmacol.

[26] Gintant G (2011). An evaluation of hERG current assay performance: translating preclinical safety studies to clinical QT prolongation. Pharmacol Ther.

[27] Pollard CE (2017). An analysis of the relationship between preclinical and clinical QT interval-related data. Toxicol Sci.

[28] Hanson LA (2006). ILSI-HESI cardiovascular safety subcommittee initiative: evaluation of three non-clinical models of QT prolongation. J Pharmacol Toxicol Methods.

[29] Kirsch GE (2004). Variability in the measurement of hERG potassium channel inhibition: effects of temperature and stimulus pattern. J Pharmacol Toxicol Methods.

[30] Crumb WJ, Vicente J, Johannesen L, Strauss DG (2016). An evaluation of 30 clinical drugs against the comprehensive *in vitro* proarrhythmia assay (CiPA) proposed ion channel panel. J Pharmacol Toxicol Methods.

[31] Sassano MF, Doak AK, Roth BL, Shoichet BK (2013). Colloidal aggregation causes inhibition of G protein-coupled receptors. J Med Chem.

[32] Owen SC (2014). Colloidal drug formulations can explain “bell-shaped” concentration-response curves. ACS Chem Biol.

[33] Waybright TJ, Britt JR, McCloud TG (2009). Overcoming problems of compound storage in DMSO: solvent and process alternatives. J Biomol Screen.

[34] Oldenburg K, Pooler D, Scudder K, Lipinski C, Kelly M (2005). High throughput sonication: evaluation for compound solubilization. Comb Chem High Throughput Screen.

[35] Mikhail A (2007). Hydroxypropyl beta-cyclodextrins: a misleading vehicle for the *in vitro* hERG current assay. J Cardiovasc Pharmacol.

[36] Houtmann S, Schombert B, Sanson C, Partiseti M, Bohme GA (2017). Automated patch-clamp methods for the hERG cardiac potassium channel. Methods Mol Biol.

[37] Bridgland-Taylor MH (2006). Optimisation and validation of a medium-throughput electrophysiology-based hERG assay using IonWorks HT. J Pharmacol Toxicol Methods.

[38] Qu Y, Schnier P, Zanon R, Vargas HM (2011). hERG potency estimates based upon dose solution analysis: what have we learned?. J Pharmacol Toxicol Methods.

[39] Rae J, Cooper K, Gates P, Watsky M (1991). Low access resistance perforated patch recordings using amphotericin B. J Neurosci Methods.

[40] Sherman AJ, Shrier A, Cooper E (1999). Series resistance compensation for whole-cell patch-clamp studies using a membrane state estimator. Biophys J.

[41] Kostyuk PG, Krishtal OA, Pidoplichko VI (1975). Effect of internal fluoride and phosphate on membrane currents during intracellular dialysis of nerve cells. Nature.

[42] Tasaki I, Takenaka T (1964). Effects of various potassium salts and proteases upon excitability of intracellularly perfused squid giant axons. Proc Natl Acad Sci USA.

[43] Orvos P (2019). Evaluation of possible proarrhythmic potency: comparison of the effect of dofetilide, cisapride, sotalol, terfenadine, and verapamil on hERG and native IKr currents and on cardiac action potential. Toxicol Sci.

[44] Windley MJ, Lee W, Vandenberg JI, Hill AP (2018). The temperature dependence of kinetics associated with drug block of hERG channels is compound-specific and an important factor for proarrhythmic risk prediction. Mol Pharmacol.

[45] Wang J (2018). Systematic performance comparison between QPatch and PatchXpresss for hERG, hINav1.5, and hIKs assays. Biophys J.

[46] Chang KC (2017). Uncertainty quantification reveals the importance of data variability and experimental design considerations for in silico proarrhythmia risk assessment. Front Physiol.

