# Publisher Correction: Cross-site and cross-platform variability of automated patch clamp assessments of drug effects on human cardiac currents in recombinant cells

**DOI:** 10.1038/s41598-020-68819-0

**Published:** 2020-07-14

**Authors:** James Kramer, Herbert M. Himmel, Anders Lindqvist, Sonja Stoelzle-Feix, Khuram W. Chaudhary, Dingzhou Li, Georg Andrees Bohme, Matthew Bridgland-Taylor, Simon Hebeisen, Jingsong Fan, Muthukrishnan Renganathan, John Imredy, Edward S. A. Humphries, Nina Brinkwirth, Tim Strassmaier, Atsushi Ohtsuki, Timm Danker, Carlos Vanoye, Liudmila Polonchuk, Bernard Fermini, Jennifer Beck Pierson, Gary Gintant

**Affiliations:** 1 Charles River Laboratories, Cleveland, OH USA; 2 Safety Pharmacology, Bayer AG, Wuppertal, Germany; 3 Sophion Bioscience A/S, Ballerup, Denmark; 4 Nanion Technologies GmbH, Munich, Germany; 5 Global Safety Pharmacology, GlaxoSmithKline PLC, Collegeville, PA USA; 6 Drug Safety Research & Development, Pfizer, Groton, CT USA; 7 Integrated Drug Discovery, High Content Biology Unit, Sanofi R&D, Vitry-Sur-Seine, France; 8 Clinical Pharmacology & Safety Sciences, BioPharmaceuticals R&D, Astra Zeneca, Cambridge, UK; 9 B’SYS GmbH, Witterswil, Switzerland; 10 Discovery Toxicology, Bristol-Myers Squibb Company, Princeton, NJ USA; 11 Eurofins Discovery, St. Charles, MO USA; 12 Merck & Co., Inc., Kenilworth, NJ USA; 13 Metrion Biosciences Ltd., Cambridge, UK; 14 Nanion Technologies, Livingston, NJ USA; 15 Nanion Technologies Japan K.K., Shinjyuku-ku, Tokyo, Japan; 16 Natural and Medical Science Institute at the University of Tübingen, Reutlingen, Germany; 17 Northwestern Feinberg School of Medicine, Chicago, IL USA; 18 Roche Pharma Research & Early Development, Roche Innovation Center Basel, F. Hoffmann-La Roche Ltd., Basel, Switzerland; 19 Novoheart, Vancouver, BC Canada; 20 Health and Environmental Sciences Institute, Washington, DC USA; 21 AbbVie, Chicago, IL USA

Correction to: *Scientific Reports* https://doi.org/10.1038/s41598-020-62344-w, published online 27 March 2020

The original version of this Article contained an error in Affiliation 12, which was incorrectly given as ‘Merck, West Point, PA, USA’. The correct affiliation is listed below:

Merck & Co., Inc., Kenilworth, NJ, USA

This error has now been corrected in the HTML and PDF versions of the Article.

