# Supplementary material for: Cross-site and cross-platform variability of automated patch clamp assessments of drug effects on human cardiac currents in recombinant cells
Source: Sci Rep. 2020 Mar 27;10:5627. doi: 10.1038/s41598-020-62344-w (PMC7101356; doi:10.1038/s41598-020-62344-w)
Supplement: Supplementary file 1 — Supporting Information. [file 41598_2020_62344_MOESM1_ESM.docx]

Kramer *et al.,* Supplementary Materials

Table of Contents

[Supplementary tables 2](#_Toc31891823)

[Table S1. Recording solutions by site, channel, and platform 2](#_Toc31891824)

[Table S2. hERG K^+^ current inhibition 5](#_Toc31891825)

[Table S3. hERG K^+^ current inhibition (continued) 7](#_Toc31891826)

[Table S4. hERG K^+^ current inhibition (continued) 9](#_Toc31891827)

[Table S5. Peak hNav1.5 Na^+^ current inhibition 11](#_Toc31891828)

[Table S6. Peak hNav1.5 Na^+^ current inhibition (continued) 13](#_Toc31891829)

[Table S7. Peak hNav1.5 Na^+^ current inhibition (continued) 15](#_Toc31891830)

[Table S8. Late hNav1.5 Na^+^ current inhibition 17](#_Toc31891831)

[Table S9. Peak hCav1.2 Ca^2+^ current inhibition 19](#_Toc31891832)

[Table S10. Peak hCav1.2 Ca^2+^ current inhibition (continued) 21](#_Toc31891833)

[Table S11. Survey results comparing experimental conditions from 10 responding sites 23](#_Toc31891834)

[Supplementary Figure Legends 24](#_Toc31891835)

[Figure S1. 24](#_Toc31891836)

[Figure S2. 24](#_Toc31891837)

[Figure S3. 24](#_Toc31891838)

[Figure S4. 25](#_Toc31891839)

[Figure S5. 25](#_Toc31891840)

[Figure S6. 25](#_Toc31891841)

[Figure S7. 26](#_Toc31891842)

[Figure S8. 26](#_Toc31891843)

[Figure S9. 26](#_Toc31891844)

Supplementary tables

Table S1. Recording solutions by site, channel, and platform

| Site number | Platform number | Channel | Internal recording solution [mmol/L] | External recording solution [mmol/L] |
| --- | --- | --- | --- | --- |
| s01 | p1 | hERG | KF120, KCl 20, HEPES 10, EGTA 10, 25 µM Escin | NaCl 80, KCl 4, HEPES 10, CaCl2 2, MgCl2 1, glucose 5, NMDG 60 |
|  |  | hNav1.5 | KF120, KCl 20, HEPES 10, EGTA 10, 25 µM Escin | NaCl 80, KCl 4, HEPES 10, CaCl2 2, MgCl2 1, glucose 5, NMDG 60 |
|  |  | hCav1.2 | KF120, KCl 20, HEPES 10, EGTA 10, 25 µM Escin | NaCl 140, KCl 4, HEPES 10, CaCl2 2, MgCl2 1, glucose 5 |
| s02 | p2 | hERG | KF120, KCl 10, NaCl 7, HEPES 10, EGTA 5 | NaCl 80, NMDG 60, KCl 4, CaCl2 2, MgCl2 1, HEPES 10, glucose 5, DMSO 0.3% |
|  |  | hNav1.5 | CsF 110, CsCl 10, HEPES 10, EGTA 20 | NaCl 80, NMDG 60, KCl 4, CaCl2 2, MgCl2 1, HEPES 10, glucose 5, DMSO 0.3% |
|  |  | hCav1.2 | CsF 110, CsCl 10, MgCl2 2, Na2ATP 5, HEPES 10, EGTA 10 | NaCl 80, NMDG 60, KCl 4, CaCl2 2, MgCl2 1, HEPES 10, glucose 5, DMSO 0.1% |
| s03 | p2 | hERG | 50 KCl, 10 NaCl, 60 KF, 20 EGTA, 10 HEPES/KOH | 140 NaCl, 4 KCl, 1.8 CaCl2, 1 MgCl2, 10 glucose, 10 HEPES |
| s04 | p3 | hERG | 130 KCl, 1 MgCl2, 5 HEPES, 5 EGTA, 7 NaCl | 137 NaCl, 4 KCl, 1 MgCl2, 1.8 CaCl2, 10 HEPES, 10 glucose |
|  |  | hNav1.5 | 130 CsCl, 1 MgCl2, 5 HEPES, 5 EGTA, 7 NaCl | 137 NaCl, 4 CsCl, 1 MgCl2, 1.8 CaCl2, 10 HEPES, 10 glucose |
|  |  | hCav1.2 | 33.75 CsF, 0.05 CaCl2, 2.5 NaCl, 81 CsMeSO3, 3.375 MgCl2, 20.5 HEPES, 8.75 EGTA, 3 ATP-Na2, 3.75 creatine, 3.75 phosphocreatine (Na2), 3.75 pyruvate (free acid), 3.75 oxalacetat | 100 NaCl, 4 KCl, 40 NMDG, 5 CaCl2 × 2H2O, 1 MgCl2 × 6H2O, 10 HEPES, 5 D-glucose, 2.5 sorbitol |
| s05 | p4 | hERG | KCl, 70, KF, 70, MgCl2, 5.0, EGTA, 2.5, HEPES, 10, escin, 14 µg/mL | NaCl, 138, KCl, 4.0, CaCl2, 3.8, HEPES, 10, glucose, 10, Kolliphor EL, 0.01% |
|  |  | hNav1.5 | CsCl, 50, CsF, 90, MgCl2, 5, EGTA, 2.5, HEPES, 10, escin, 14 µg/mL | NaCl, 138, KCl, 4.0, CaCl 3.8, HEPES, 10, glucose, 10, Kolliphor EL, 0.01% |
|  |  | hCav1.2 | CsCl, 50, CsF, 90, MgCl2, 5, EGTA, 2.5, HEPES, 10, escin, 14 µg/mL | NaCl, 138, KCl, 4.0, CaCl2, 7.0, HEPES, 10, glucose, 10, Kolliphor EL, 0.01% |
| s06 | p4 | hERG | KCl, 70, KF, 70, MgCl2, 5.0, EGTA, 2.5, HEPES, 10, escin, 14 µg/mL | NaCl, 138, KCl, 4.0, CaCl 3.8, HEPES, 10, glucose, 10, Kolliphor EL, 0.01% |
| s07 | p5 | hERG | Proprietary | 137 NaCl, 4 KCl, 1 MgCl2, 1.8 CaCl2, 10 HEPES, 11 glucose |
|  |  | hNav1.5 | Proprietary | 137 NaCl, 4 CsCl, 1 MgCl2, 1.8 CaCl2, 10, HEPES, 10 glucose; late hNav1.5: ATX-II, 10 nmol/L |
| s10 | p3 | hERG | 130 KCl, 1 MgCl2, 5 HEPES, 5 EGTA, 7 NaCl | 137 NaCl, 4 KCl, 1 MgCl2, 1.8 CaCl2, 10 HEPES, 11 glucose |
|  |  | hNav1.5 | 130 CsCl, 1 MgCl2, 5 HEPES, 5 EGTA, 7 NaCl | 137 NaCl, 4 CsCl, 1 MgCl2, 1.8 CaCl2, 10 HEPES, 11 glucose |
| s11 | p1 | hERG | 120 KF, 20 KCI, 10 HEPES, 10 EGTA | 80 NaCl, 60 NMDG, 10 HEPES, 4 KCl, 2 CaCl, 1 MgCl |
|  |  | hNav1.5 | 140 CsF, 10 NaCl, 10 HEPES, 5 EGTA, 10 sucrose | 80 NaCl, 60 NMDG, 10 HEPES, 4 KCl, 2 CaCl, 1 MgCl |
|  |  | hCav1.2 | 140 CsF, 10 NaCl, 10 HEPES, 5 EGTA, 10 sucrose, 0.3 µmol/L CaM, 3 ATP-Na | 140 NaCl, 4 KCl, 2 CaCl, 1 MgCl, 5 D-glucose, 10 HEPES |
| s12 | p2 | hERG | 120 KF, 10 KCl, 7 NaCl, 5 EGTA, 10 HEPES | 80 NaCl, 60 NMDG, 10 HEPES, 4 KCl, 2 CaCl2, 1 MgCl2 |
|  |  | hNav1.5 | 120 CsF, 10 CsCl, 10 NaCl, 10 EGTA, 10 HEPES | 80 NaCl, 60 NMDG, 10 HEPES, 4 KCl, 2 CaCl2, 1 MgCl2 |
| s13 | p2 | hERG | 120 KF, 10 KCl, 7 NaCl, 5 EGTA, 10 HEPES | 80 NaCl, 60 NMDG, 10 HEPES, 4 KCl, 2 CaCl2, 1 MgCl2 |
|  |  | hNav1.5 | 120 CsF, 10 CsCl, 10 NaCl, 10 EGTA, 10 HEPES | 80 NaCl, 60 NMDG, 10 HEPES, 4 KCl, 2 CaCl2, 1 MgCl2 |
| s14 | p1 | hERG | 120 KF, 20 KCl, 10 HEPES, 5 EGTA, 7 NaCl | 80 NaCl, 60 NMDG, 10 HEPES, 4 KCl, 2 CaCl, 1 MgCl |
|  |  | hNav1.5 | 120 CsF, 10 EGTA, 10 HEPES, 10 NaCl | 80 NaCl, 10 HEPES, 4 KCl, 2 CaCl2, 1 MgCl2, 60 NMDG, 5 glucose |
|  |  | hCav1.2 | 120 CsF, 10 NaCl, 10 EGTA, 10 HEPES, 25 µmol/L escin, 1 Na-ATP | 80 NaCl, 60 NMDG, 10 HEPES, 4 KCl, 2 CaCl, 1 MgCl, 0.3% DMSO |
| s15 | p1 | hERG | 120 KF, 20 KCl, 10 HEPES, 10 EGTA | 80 NaCl, 60 NMDG, 10 HEPES, 4 KCl, 2 CaCl, 1 MgCl |
|  |  | hNav1.5 | 120 CsF, 10 CsCl, 10 NaCl, 10 EGTA, 10 HEPES | 80 NaCl, 60 NMDG, 10 HEPES, 4 KCl, 2 CaCl, 1 MgCl |
| s16 | p3 | hERG | KCl 120, CaCl2 5.3, MgCl2 1.75, HEPES 10 | NaCl 145, KCl 4, D-glucose 10, MgCl2 1, HEPES 10, CaCl2 2 |
|  |  | hNav1.5 | CsF 140, NaCl 10, EGTA 1, HEPES 10 | NaCl 140 , KCl 3, CaCl2 1, MgCl2 1, HEPES 20 |
|  |  | hCav1.2 | CsCl 112, CsF 27, EGTA 8.2, HEPES 10, NaCl 2 | NaCl 145, KCl 4, HEPES 10, CaCl2 10 |
| s17 | p1 | hNav1.5 | CsF, 100, CsCl, 10, NaCl, 10, HEPES, 10, EGTA, 20 | NaCl, 140, KCl, 4, CaCl2, 2, MgCl2, 1, HEPES, 10, glucose, 5 |
| s18 | p1 | hERG | 10 KCl, 100 KF, 10 NaCl, 10 HEPES, 20 EGTA | 80 NaCl, 4 KCl, 1 MgCl2, 1 CaCl2, 10 HEPES, 5 glucose, 40 NMDG, 0.2% DMSO |
|  |  | hNav1.5 | 120 CsF, 10 EGTA, 10 HEPES, 10 NaCl | 80 NaCl, 10 HEPES, 4 KCl, 2 CaCl2, 1 MgCl2, 60 NMDG, 5 glucose, 0.2% DMSO |
|  |  | hCav1.2 | 120 CsF, 10 EGTA, 10 HEPES, 10 NaCl, 1 Na-ATP | 80 NaCl, 10 HEPES, 4 KCl, 2 CaCl2, 1 MgCl2, 60 NMDG, 5 glucose, 0.2% DMSO |
| s19 | p3 | hERG | 130 KCl, 1 MgCl2, 5 HEPES, 5 EGTA, 7 NaCl | 137 NaCl, 4 KCl, 1 MgCl2, 1.8 CaCl2, 10 HEPES, 11 glucose |
|  |  | hNav1.5 | 130 CsCl, 1 MgCl2, 5 HEPES, 5 EGTA, 7 NaCl | 137 NaCl, 4 CsCl, 1 MgCl2, 1.8 CaCl2, 10 HEPES, 11 glucose |

Table S2. hERG K^+^ current inhibition

| Drug^a^ | Min/max concentration^b^ log IC_50_, n_H_ | Site number_platform number | | | | | | | | | | | | | | | | | |
| --- | --- | --- | --- | --- | --- | --- | --- | --- | --- | --- | --- | --- | --- | --- | --- | --- | --- | --- | --- |
|  |  | s01_p1 | | | s02_p2 | | | s03_p2 | | | s04_p3 | | | s05_p4 | | | s06_p4 | | |
|  |  | Mean [%] | SD [%] | n | Mean [%] | SD [%] | n | Mean [%] | SD [%] | n | Mean [%] | SD [%] | n | Mean [%] | SD [%] | n | Mean [%] | SD [%] | n |
| bep | 1.00E-08 | 5.2 | 15.2 | 10 | −1.1 | 2.0 | 5 | 10.9 | 8.1 | 4 | 16.3 | 6.6 | 3 | 2.2 | 23.1 | 6 | 3.6 | 14.8 | 6 |
|  | 3.00E-07 | 78.8 | 8.7 | 10 | 53.8 | 9.4 | 5 | 98.9 | 2.6 | 4 | 65.3 | 4.7 | 3 | 76.5 | 6.6 | 6 | 56.3 | 8.6 | 5 |
|  | IC_50_ | −6.97 |  |  | −6.55 |  |  | −7.21 |  |  | −6.69 |  |  | −6.91 |  |  | −6.56 |  |  |
|  | n_H_ | 1.25 |  |  | 1.41 |  |  | 1.50 |  |  | 0.99 |  |  | 1.21 |  |  | 1.48 |  |  |
| cpz | 1.00E-07 |  |  |  |  |  |  | 13.5 | 8.0 | 4 |  |  |  | −0.4 | 14.4 | 5 | 1.4 | 14.5 | 6 |
|  | 3.00E-06 |  |  |  |  |  |  | 96.9 | 5.9 | 4 |  |  |  | 94.8 | 6.7 | 6 | 84.8 | 4.1 | 5 |
|  | IC_50_ |  |  |  |  |  |  | −6.33 |  |  |  |  |  | −6.24 |  |  | −6.03 |  |  |
|  | n_H_ |  |  |  |  |  |  | 1.30 |  |  |  |  |  | 1.62 |  |  | 1.59 |  |  |
| cis | 1.00E-09 | 4.1 | 12.5 | 11 | 1.6 | 4.2 | 5 | 17.3 | 10.0 | 3 | 6.8 | 5.8 | 3 | 2.4 | 17.4 | 6 | −9.9 | 9.5 | 6 |
|  | 3.00E-07 | 69.4 | 14.8 | 10 | 80.5 | 5.4 | 5 | 92.8 | 4.0 | 3 | 80.3 | 0.9 | 3 | 97.8 | 2.8 | 6 | 95.3 | 12.1 | 5 |
|  | IC_50_ | −6.97 |  |  | −7.02 |  |  | −7.66 |  |  | −6.90 |  |  | −7.61 |  |  | −7.19 |  |  |
|  | n_H_ | 0.85 |  |  | 1.10 |  |  | 0.63 |  |  | 1.35 |  |  | 1.50 |  |  | 1.58 |  |  |
| dil | 3.00E-06 | 18.7 | 17.4 | 9 | 13.7 | 2.2 | 6 | 25.9 | 8.5 | 4 | 37.9 | 10.9 | 3 | 21.3 | 12.4 | 6 | 44.6 | 14.0 | 5 |
|  | 1.00E-04 | 72.2 | 9.0 | 11 | 82.6 | 4.3 | 6 | 94.8 | 3.5 | 4 | 96.6 | 0.3 | 3 | 95.7 | 1.9 | 6 | 99.5 | 4.2 | 6 |
|  | IC_50_ | −4.63 |  |  | −4.71 |  |  | −4.95 |  |  | −5.23 |  |  | −7.61 |  |  | −7.19 |  |  |
|  | n_H_ | 0.75 |  |  | 0.93 |  |  | 0.98 |  |  | 0.89 |  |  | 1.50 |  |  | 1.58 |  |  |
| dof | 1.00E-09 | −6.9 | 16.0 | 13 | 1.3 | 1.7 | 6 | 11.8 | 6.9 | 3 |  |  |  | −5.6 | 15.8 | 5 | 2.9 | 17.9 | 6 |
|  | 1.00E-07 | 47.6 | 20.5 | 10 | 39.5 | 11.0 | 12 | 51.2 | 0.8 | 3 |  |  |  | 98.6 | 1.4 | 6 | 102.1 | 4.3 | 5 |
|  | IC_50_ | −6.98 |  |  | −6.83 |  |  | −7.08 |  |  |  |  |  | −8.08 |  |  | −7.87 |  |  |
|  | n_H_ | 2.00 |  |  | 1.07 |  |  | 0.41 |  |  |  |  |  | 2.00 |  |  | 1.30 |  |  |
| mex | 1.00E-06 | 0.7 | 9.2 | 14 | 1.0 | 0.8 | 3 | 14.6 | 11.1 | 3 | 9.7 | 7.0 | 3 | 2.2 | 23.1 | 6 | −3.4 | 7.3 | 6 |
|  | 1.00E-04 | 46.1 | 15.7 | 12 | 48.2 | 3.2 | 3 | 86.8 | 2.9 | 3 | 67.4 | 4.2 | 3 | 75.7 | 8.3 | 6 | 71.6 | 5.3 | 5 |
|  | IC_50_ | −3.86 |  |  | −3.96 |  |  | −4.69 |  |  | −4.32 |  |  | −4.37 |  |  | −4.43 |  |  |
|  | n_H_ | 0.78 |  |  | 0.95 |  |  | 0.95 |  |  | 0.81 |  |  | 1.45 |  |  | 1.02 |  |  |
| ond | 3.00E-07 | 8.8 | 10.2 | 5 | 6.1 | 4.5 | 5 | 37.2 | 9.3 | 3 | 22.1 | 6.3 | 4 | 9.6 | 25.7 | 6 | 25.3 | 11.9 | 6 |
|  | 1.00E-05 | 83.1 | 6.8 | 8 | 79.8 | 3.1 | 5 | 99.9 | 0.2 | 3 | 90.6 | 3.3 | 4 | 93.2 | 4.5 | 6 | 95.5 | 6.7 | 6 |
|  | IC_50_ | −5.74 |  |  | −5.59 |  |  | −6.27 |  |  | −5.92 |  |  | −5.84 |  |  | −6.05 |  |  |
|  | n_H_ | 1.06 |  |  | 1.07 |  |  | 1.08 |  |  | 0.97 |  |  | 1.32 |  |  | 1.05 |  |  |
| qui | 1.00E-07 | 8.9 | 10.1 | 10 | 4.1 | 1.6 | 4 | 39.8 | 5.6 | 3 | 25.6 | 11.3 | 3 | −6.7 | 9.1 | 4 | 5.3 | 10.3 | 5 |
|  | 1.00E-05 | 81.2 | 14.2 | 11 | 85.2 | 2.0 | 4 | 97.4 | 2.3 | 3 | 99.6 | 5.4 | 3 | 94.2 | 2.9 | 6 | 92.8 | 4.1 | 6 |
|  | IC_50_ | −5.71 |  |  | −5.75 |  |  | −6.80 |  |  | −6.35 |  |  | −6.02 |  |  | −6.10 |  |  |
|  | n_H_ | 0.87 |  |  | 1.03 |  |  | 0.98 |  |  | 0.85 |  |  | 1.37 |  |  | 0.99 |  |  |
| ran | 3.00E-06 | 25.5 | 16.3 | 11 | 20.7 | 1.3 | 4 | 10.6 | 5.5 | 3 | 20.8 | 3.0 | 5 | 26.9 | 15.3 | 6 | 46.6 | 5.7 | 5 |
|  | 1.00E-04 | 81.3 | 4.2 | 10 | 87.3 | 1.7 | 4 | 74.7 | 13.3 | 3 | 94.2 | 2.6 | 5 | 97.9 | 7.2 | 6 | 100.2 | 2.1 | 6 |
|  | IC_50_ | −4.94 |  |  | −4.96 |  |  | −4.41 |  |  | −4.95 |  |  | −5.13 |  |  | −5.45 |  |  |
|  | n_H_ | 0.83 |  |  | 0.98 |  |  | 1.13 |  |  | 1.10 |  |  | 1.15 |  |  | 1.14 |  |  |
| sot | 1.00E-05 | −1.0 | 10.0 | 6 | 0.9 | 3.2 | 6 | 3.3 | 9.9 | 4 | 12.2 | 2.3 | 3 | −9.2 | 15.7 | 5 | 0.9 | 17.6 | 5 |
|  | 3.00E-04 | 35.9 | 11.2 | 9 | 34.4 | 15.5 | 6 | 49.2 | 5.8 | 4 | 70.5 | 8.8 | 3 | 53.8 | 10.3 | 6 | 52.0 | 8.7 | 6 |
|  | IC_50_ | −3.07 |  |  | −3.27 |  |  | −3.49 |  |  | −3.91 |  |  | −3.56 |  |  | −3.53 |  |  |
|  | n_H_ | 0.52 |  |  | 1.20 |  |  | 1.08 |  |  | 0.86 |  |  | 1.38 |  |  | 1.18 |  |  |
| ter | 1.00E-08 | 17.1 | 14.0 | 10 | −1.6 | 0.9 | 3 | 6.9 | 7.0 | 3 | 3.5 | 2.4 | 3 | 11.2 | 16.2 | 5 | 8.1 | 9.4 | 4 |
|  | 3.00E-07 | 87.4 | 6.6 | 10 | 69.9 | 2.7 | 3 | 99.1 | 1.5 | 3 | 22.8 | 4.1 | 3 | 92.2 | 4.1 | 6 | 59.9 | 8.4 | 4 |
|  | IC_50_ | −6.93 |  |  | −6.80 |  |  | −7.22 |  |  | −5.97 |  |  | −7.23 |  |  | −6.78 |  |  |
|  | n_H_ | 1.72 |  |  | 1.30 |  |  | 1.62 |  |  | 1.01 |  |  | 1.28 |  |  | 0.94 |  |  |
| ver | 3.00E-08 | 2.1 | 8.3 | 11 | 2.3 | 1.4 | 5 | 11.3 | 12.5 | 4 | 2.4 | 5.5 | 4 | 9.8 | 14.6 | 5 | 5.6 | 14.5 | 6 |
|  | 1.00E-06 | 62.7 | 8.8 | 10 | 56.6 | 5.3 | 5 | 87.9 | 7.8 | 4 | 60.5 | 6.2 | 4 | 89.0 | 6.7 | 6 | 92.0 | 5.3 | 6 |
|  | IC_50_ | −6.20 |  |  | −6.11 |  |  | −6.62 |  |  | −6.13 |  |  | −6.71 |  |  | −6.76 |  |  |
|  | n_H_ | 1.36 |  |  | 1.11 |  |  | 1.15 |  |  | 1.33 |  |  | 1.32 |  |  | 1.29 |  |  |

^a^bep, bepridil; cpz, chlorpromazine; cis, cisapride; dil, diltiazem; dof, dofetilide; mex, mexiletine; ond, ondansetron; qui, quinidine; ran, ranolazine; sot, sotalol; ter, terfenadine; ver, verapamil.

^b^min/max concentration, minimal and maximal concentration [mol/L]; IC_50_, half-maximal inhibitory concentration [log mol/L]; n_H_, Hill slope.

Table S3. hERG K^+^ current inhibition (continued)

| Drug^a^ | Min/max concentration^b^ log IC_50_, n_H_ | Site number_platform number^c^ | | | | | | | | | | | | | | | | | |
| --- | --- | --- | --- | --- | --- | --- | --- | --- | --- | --- | --- | --- | --- | --- | --- | --- | --- | --- | --- |
|  |  | s07_p5 | | | s10_p3 | | | s11_p1 | | | s12_p2 | | | s13_p2 | | | s14_p1 | | |
|  |  | Mean [%] | SD [%] | n | Mean [%] | SD [%] | n | Mean [%] | SD [%] | n | Mean [%] | SD [%] | n | Mean [%] | SD [%] | n | Mean [%] | SD [%] | n |
| bep | 1.00E-08 | 0.5 | 5.0 | 19 | 1.5 | 5.5 | 4 | 12.0 | 14.7 | 14 | 2.3 | 4.6 | 5 | 4.3 | 5.1 | 10 | 41.9 | 19.3 | 12 |
|  | 3.00E-07 | 85.5 | 14.7 | 19 | 59.6 | 8.5 | 4 | 90.8 | 4.8 | 15 | 66.8 | 11.5 | 5 | 61.5 | 8.1 | 10 | 79.8 | 7.3 | 15 |
|  | IC_50_ | −6.97 |  |  | −6.62 |  |  | −7.32 |  |  | −6.74 |  |  | −6.68 |  |  | −7.33 |  |  |
|  | n_H_ | 1.66 |  |  | 1.36 |  |  | 1.00 |  |  | 1.27 |  |  | 1.05 |  |  | 0.58 |  |  |
| cpz | 1.00E-07 | −0.4 | 4.9 | 19 |  |  |  |  |  |  |  |  |  |  |  |  |  |  |  |
|  | 3.00E-06 | 88.3 | 9.3 | 19 |  |  |  |  |  |  |  |  |  |  |  |  |  |  |  |
|  | IC_50_ | −5.95 |  |  |  |  |  |  |  |  |  |  |  |  |  |  |  |  |  |
|  | n_H_ | 1.97 |  |  |  |  |  |  |  |  |  |  |  |  |  |  |  |  |  |
| cis | 1.00E-09 | −6.4 | 14.5 | 18 | 7.2 | 6.9 | 3 | 7.0 | 23.3 | 14 | −1.0 | 4.5 | 5 | 9.5 | 3.4 | 6 | 12.0 | 5.9 | 11 |
|  | 3.00E-07 | 130.4 | 23.3 | 18 | 68.5 | 18.3 | 3 | 93.3 | 5.8 | 16 | 80.3 | 6.5 | 5 | 80.0 | 6.5 | 6 | 77.0 | 10.8 | 15 |
|  | IC_50_ | −7.83 |  |  | −6.92 |  |  | −6.96 |  |  | −6.95 |  |  | −7.01 |  |  | −7.21 |  |  |
|  | n_H_ | 2.00 |  |  | 0.65 |  |  | 2.00 |  |  | 1.32 |  |  | 0.90 |  |  | 0.63 |  |  |
| dil | 3.00E-06 | 12.8 | 11.8 | 16 | 19.3 | 2.8 | 4 | 36.1 | 9.3 | 16 | 14.4 | 4.5 | 4 | 15.4 | 1.0 | 3 | 43.6 | 22.1 | 15 |
|  | 1.00E-04 | 116.5 | 22.7 | 16 | 83.8 | 3.6 | 4 | 99.8 | 13.0 | 14 | 90.5 | 1.2 | 4 | 90.0 | 4.5 | 3 | 83.3 | 8.0 | 15 |
|  | IC_50_ | −4.73 |  |  | −4.79 |  |  | −5.23 |  |  | −4.83 |  |  | −4.81 |  |  | −5.36 |  |  |
|  | n_H_ | 1.80 |  |  | 0.89 |  |  | 0.99 |  |  | 1.12 |  |  | 1.09 |  |  | 0.60 |  |  |
| dof | 1.00E-09 | −10.5 | 14.1 | 17 | 4.8 | 5.1 | 3 | 7.9 | 17.0 | 15 | 0.5 | 3.2 | 6 | 8.3 | 6.1 | 9 | 28.3 | 17.6 | 11 |
|  | 1.00E-07 | −23.3 | 19.5 | 17 | 43.9 | 2.6 | 3 | 95.6 | 3.1 | 16 | 90.3 | 4.1 | 6 | 91.5 | 4.7 | 9 | 88.5 | 6.3 | 9 |
|  | IC_50_ | fnc |  |  | −6.89 |  |  | −7.73 |  |  | −7.52 |  |  | −7.92 |  |  | −8.02 |  |  |
|  | n_H_ | fnc |  |  | 0.99 |  |  | 1.36 |  |  | 1.86 |  |  | 0.97 |  |  | 0.55 |  |  |
| mex | 1.00E-06 | −10.5 | 10.9 | 12 | −3.5 | 6.9 | 3 | 29.4 | 10.4 | 12 | 0.2 | 2.5 | 9 | 7.7 | 7.1 | 5 | 10.8 | 7.6 | 16 |
|  | 1.00E-04 | 65.0 | 17.8 | 12 | 42.0 | 7.6 | 3 | 73.7 | 12.2 | 13 | 56.6 | 9.2 | 9 | 78.6 | 8.3 | 5 | 60.2 | 7.8 | 16 |
|  | IC_50_ | −4.14 |  |  | −3.90 |  |  | −4.80 |  |  | −4.11 |  |  | −4.46 |  |  | −4.20 |  |  |
|  | n_H_ | 1.95 |  |  | 1.32 |  |  | 0.41 |  |  | 1.12 |  |  | 1.01 |  |  | 0.63 |  |  |
| ond | 3.00E-07 | 17.0 | 3.8 | 16 | 9.7 | 1.3 | 3 | 11.6 | 21.8 | 16 | 19.3 | 6.7 | 7 | 11.4 | 4.0 | 4 | 58.7 | 28.2 | 11 |
|  | 1.00E-05 | 100.7 | 9.9 | 16 | 71.8 | 2.0 | 3 | 89.6 | 3.8 | 15 | 91.6 | 6.2 | 7 | 90.0 | 2.1 | 4 | 89.4 | 4.6 | 14 |
|  | IC_50_ | −5.87 |  |  | −5.40 |  |  | −5.84 |  |  | −5.94 |  |  | −5.81 |  |  | −6.51 |  |  |
|  | n_H_ | 1.26 |  |  | 0.98 |  |  | 1.10 |  |  | 1.03 |  |  | 1.19 |  |  | 0.47 |  |  |
| qui | 1.00E-07 | −1.0 | 10.2 | 19 | 12.2 | 4.2 | 3 | 13.1 | 13.9 | 14 | 6.2 | 2.6 | 7 | 12.5 | 2.8 | 4 | 14.8 | 6.8 | 12 |
|  | 1.00E-05 | 126.0 | 17.4 | 19 | 89.3 | 3.6 | 3 | 94.0 | 7.3 | 16 | 94.6 | 2.6 | 7 | 95.1 | 9.8 | 4 | 88.1 | 5.2 | 14 |
|  | IC_50_ | −6.15 |  |  | −5.83 |  |  | −6.10 |  |  | −5.98 |  |  | −6.07 |  |  | −6.01 |  |  |
|  | n_H_ | 2.00 |  |  | 0.96 |  |  | 1.00 |  |  | 1.17 |  |  | 0.97 |  |  | 0.82 |  |  |
| ran | 3.00E-06 | 30.0 | 4.5 | 16 | 16.1 | 2.3 | 3 | 26.8 | 19.5 | 16 | 19.5 | 1.9 | 3 | 19.4 | 5.2 | 4 | 41.6 | 20.2 | 15 |
|  | 1.00E-04 | 108.0 | 12.7 | 19 | 78.9 | 4.0 | 3 | 94.8 | 3.1 | 16 | 87.3 | 1.2 | 3 | 88.1 | 3.4 | 4 | 96.4 | 3.0 | 16 |
|  | IC_50_ | −5.23 |  |  | −4.70 |  |  | −5.16 |  |  | −4.91 |  |  | −4.90 |  |  | −5.33 |  |  |
|  | n_H_ | 1.50 |  |  | 0.86 |  |  | 1.11 |  |  | 0.96 |  |  | 0.98 |  |  | 0.91 |  |  |
| sot | 1.00E-05 | −6.5 | 17.1 | 14 | 3.3 | 4.4 | 3 | 15.7 | 13.8 | 16 | 5.2 | 3.6 | 4 | 8.6 | 5.1 | 5 | 34.3 | 6.9 | 9 |
|  | 3.00E-04 | 70.1 | 27.5 | 14 | 39.3 | 8.3 | 3 | 75.1 | 8.0 | 16 | 65.0 | 3.3 | 4 | 63.0 | 5.5 | 5 | 79.3 | 6.9 | 11 |
|  | IC_50_ | −3.74 |  |  | −3.33 |  |  | −4.10 |  |  | −3.79 |  |  | −3.76 |  |  | −4.24 |  |  |
|  | n_H_ | 1.69 |  |  | 1.08 |  |  | 0.76 |  |  | 0.94 |  |  | 0.86 |  |  | 0.56 |  |  |
| ter | 1.00E-08 | −0.6 | 10.8 | 20 | 5.2 | 1.8 | 3 | 16.9 | 23.2 | 16 | −0.2 | 4.6 | 5 | 16.1 | 1.3 | 3 | 29.5 | 15.0 | 16 |
|  | 3.00E-07 | 93.5 | 13.3 | 20 | 44.6 | 3.5 | 3 | 90.1 | 4.4 | 13 | 90.8 | 1.3 | 5 | 75.0 | 4.7 | 3 | 87.7 | 8.1 | 14 |
|  | IC_50_ | −7.12 |  |  | −6.40 |  |  | −7.06 |  |  | −7.08 |  |  | −6.96 |  |  | −7.41 |  |  |
|  | n_H_ | 1.86 |  |  | 1.08 |  |  | 1.16 |  |  | 1.83 |  |  | 0.84 |  |  | 0.81 |  |  |
| ver | 3.00E-08 | 0.0 | 5.6 | 22 | 8.0 | 6.7 | 3 | 18.8 | 26.1 | 15 | 2.7 | 7.1 | 7 | 7.5 | 7.1 | 7 | 20.4 | 31.9 | 13 |
|  | 1.00E-06 | 78.0 | 13.0 | 22 | 49.4 | 7.1 | 3 | 80.6 | 10.9 | 15 | 70.2 | 3.6 | 7 | 58.4 | 6.6 | 7 | 71.2 | 10.2 | 16 |
|  | IC_50_ | −6.30 |  |  | −5.96 |  |  | −6.72 |  |  | −6.31 |  |  | −6.16 |  |  | −6.37 |  |  |
|  | n_H_ | 1.81 |  |  | 0.91 |  |  | 0.83 |  |  | 1.19 |  |  | 0.81 |  |  | 0.96 |  |  |

^a^bep, bepridil; cpz, chlorpromazine; cis, cisapride; dil, diltiazem; dof, dofetilide; mex, mexiletine; ond, ondansetron; qui, quinidine; ran, ranolazine; sot, sotalol; ter, terfenadine; ver, verapamil.

^b^min/max concentration, minimal and maximal concentration [mol/L]; IC_50_, half-maximal inhibitory concentration [log mol/L]; n_H_, Hill slope.

^c^fnc, fit did not converge.

Table S4. hERG K^+^ current inhibition (continued)

| Drug^a^ | Min/max concentration^b^ log IC_50_, n_H_ | Site number_platform number | | | | | | | | | | | |
| --- | --- | --- | --- | --- | --- | --- | --- | --- | --- | --- | --- | --- | --- |
|  |  | s15_p1 | | | s16_p3 | | | s18_p1 | | | s19_p3 | | |
|  |  | Mean [%] | SD [%] | n | Mean [%] | SD [%] | n | Mean [%] | SD [%] | n | Mean [%] | SD [%] | n |
| bep | 1.00E-08 | 10.3 | 6.1 | 16 | 4.1 | 6.9 | 3 | 9.7 | 4.0 | 11 | 3.6 | 4.1 | 8 |
|  | 3.00E-07 | 89.8 | 3.0 | 14 | 60.2 | 1.4 | 3 | 91.3 | 2.9 | 8 | 47.6 | 3.9 | 8 |
|  | IC_50_ | −7.20 |  |  | −6.64 |  |  | −7.31 |  |  | −6.46 |  |  |
|  | n_H_ | 1.26 |  |  | 1.29 |  |  | 1.32 |  |  | 1.10 |  |  |
| cpz | 1.00E-07 | 13.0 | 5.5 | 12 |  |  |  |  |  |  |  |  |  |
|  | 3.00E-06 | 93.8 | 4.0 | 14 |  |  |  |  |  |  |  |  |  |
|  | IC_50_ | −6.24 |  |  |  |  |  |  |  |  |  |  |  |
|  | n_H_ | 1.34 |  |  |  |  |  |  |  |  |  |  |  |
| cis | 1.00E-09 | 29.6 | 25.0 | 20 | 3.4 | 4.2 | 4 | −6.8 | 4.9 | 6 | 9.8 | 2.9 | 12 |
|  | 3.00E-07 | 87.3 | 7.5 | 23 | 84.9 | 2.7 | 4 | 92.5 | 3.7 | 6 | 89.5 | 3.4 | 12 |
|  | IC_50_ | −7.80 |  |  | −7.17 |  |  | −7.11 |  |  | −7.30 |  |  |
|  | n_H_ | 0.50 |  |  | 1.02 |  |  | 1.82 |  |  | 0.90 |  |  |
| dil | 3.00E-06 | 38.9 | 15.9 | 55 | 18.9 | 2.9 | 3 | −4.0 | 1.7 | 3 | 26.5 | 2.2 | 11 |
|  | 1.00E-04 | 92.2 | 4.9 | 44 | 86.0 | 0.9 | 3 | 88.8 | 2.7 | 5 | 92.8 | 2.9 | 11 |
|  | IC_50_ | −5.24 |  |  | −4.83 |  |  | −4.55 |  |  | −5.03 |  |  |
|  | n_H_ | 0.79 |  |  | 0.91 |  |  | 1.71 |  |  | 0.98 |  |  |
| dof | 1.00E-09 | 38.4 | 15.9 | 24 | 1.7 | 3.6 | 4 | 1.9 | 8.7 | 3 | 1.9 | 2.9 | 6 |
|  | 1.00E-07 | 91.3 | 3.0 | 20 | 50.1 | 4.5 | 4 | 94.2 | 2.0 | 5 | 46.1 | 8.4 | 6 |
|  | IC_50_ | −8.54 |  |  | −7.00 |  |  | −7.70 |  |  | −6.93 |  |  |
|  | n_H_ | 0.62 |  |  | 0.84 |  |  | 1.58 |  |  | 1.01 |  |  |
| mex | 1.00E-06 | 10.3 | 23.3 | 30 | 3.9 | 1.7 | 4 | −1.2 | 1.8 | 3 | 4.2 | 2.8 | 6 |
|  | 1.00E-04 | 74.6 | 5.3 | 25 | 52.6 | 11.6 | 4 | 70.4 | 7.1 | 7 | 58.4 | 7.1 | 6 |
|  | IC_50_ | −4.62 |  |  | −4.07 |  |  | −4.32 |  |  | −4.20 |  |  |
|  | n_H_ | 0.72 |  |  | 0.76 |  |  | 1.12 |  |  | 0.84 |  |  |
| ond | 3.00E-07 | 25.2 | 12.3 | 26 | 11.9 | 3.2 | 4 | 12.7 | 20.9 | 3 | 25.8 | 2.5 | 11 |
|  | 1.00E-05 | 87.4 | 4.8 | 37 | 84.1 | 2.3 | 4 | 85.6 | 9.9 | 4 | 92.9 | 3.3 | 11 |
|  | IC_50_ | −6.00 |  |  | −5.72 |  |  | −5.65 |  |  | −5.90 |  |  |
|  | n_H_ | 0.88 |  |  | 1.01 |  |  | 1.05 |  |  | 1.01 |  |  |
| qui | 1.00E-07 | 27.1 | 19.5 | 26 | 7.5 | 2.0 | 3 | 13.3 | 4.7 | 4 | 12.1 | 5.3 | 8 |
|  | 1.00E-05 | 92.4 | 4.3 | 28 | 91.6 | 1.0 | 3 | 94.4 | 1.0 | 9 | 95.7 | 1.7 | 8 |
|  | IC_50_ | −6.26 |  |  | −5.93 |  |  | −6.08 |  |  | −6.09 |  |  |
|  | n_H_ | 0.75 |  |  | 1.09 |  |  | 1.58 |  |  | 1.08 |  |  |
| ran | 3.00E-06 | 56.1 | 9.5 | 16 | 27.0 | 3.6 | 3 | −22.2 | 7.8 | 10 | 29.0 | 5.1 | 13 |
|  | 1.00E-04 | 93.1 | 4.7 | 22 | 90.8 | 1.4 | 3 | 93.7 | 2.4 | 22 | 96.9 | 2.3 | 13 |
|  | IC_50_ | −5.66 |  |  | −5.07 |  |  | −5.04 |  |  | −5.07 |  |  |
|  | n_H_ | 0.67 |  |  | 0.94 |  |  | 1.22 |  |  | 1.03 |  |  |
| sot | 1.00E-05 | 38.5 | 15.2 | 22 | 4.4 | 2.8 | 4 | −1.4 | 6.4 | 3 | 5.0 | 4.1 | 9 |
|  | 3.00E-04 | 71.5 | 7.4 | 22 | 48.0 | 14.7 | 4 | 43.7 | 3.1 | 3 | 36.8 | 11.5 | 9 |
|  | IC_50_ | −4.31 |  |  | −3.46 |  |  | −3.44 |  |  | −3.24 |  |  |
|  | n_H_ | 0.42 |  |  | 0.92 |  |  | 0.88 |  |  | 0.76 |  |  |
| ter | 1.00E-08 | 8.0 | 11.5 | 10 | 4.4 | 1.4 | 3 | −14.4 | 6.4 | 5 | 2.6 | 4.7 | 12 |
|  | 3.00E-07 | 88.1 | 6.0 | 13 | 51.0 | 9.4 | 3 | 90.6 | 4.2 | 3 | 45.9 | 10.3 | 12 |
|  | IC_50_ | −7.18 |  |  | −6.51 |  |  | −7.10 |  |  | −6.43 |  |  |
|  | n_H_ | 1.27 |  |  | 0.97 |  |  | 1.69 |  |  | 1.02 |  |  |
| ver | 3.00E-08 | 11.5 | 6.7 | 12 | 1.9 | 1.7 | 4 | −24.3 | 11.6 | 5 | 8.3 | 6.4 | 12 |
|  | 1.00E-06 | 85.5 | 3.7 | 15 | 63.0 | 3.1 | 4 | 72.1 | 12.7 | 3 | 69.5 | 8.1 | 12 |
|  | IC_50_ | −6.69 |  |  | −6.19 |  |  | −6.23 |  |  | −6.33 |  |  |
|  | n_H_ | 1.10 |  |  | 1.28 |  |  | 1.81 |  |  | 1.04 |  |  |

^a^bep, bepridil; cpz, chlorpromazine; cis, cisapride; dil, diltiazem; dof, dofetilide; mex, mexiletine; ond, ondansetron; qui, quinidine; ran, ranolazine; sot, sotalol; ter, terfenadine; ver, verapamil.

^b^min/max concentration, minimal and maximal concentration [mol/L]; IC_50_, half-maximal inhibitory concentration [log mol/L]; n_H_, Hill slope.

^c^Please note that s20_m used concentrations different from those in column 2.

Table S5. Peak hNav1.5 Na^+^ current inhibition

| Drug^a^ | Min/max conentration^b^ log IC_50_, n_H_ | Site number_platform number^c^ | | | | | | | | | | | | | | |
| --- | --- | --- | --- | --- | --- | --- | --- | --- | --- | --- | --- | --- | --- | --- | --- | --- |
|  |  | s01_p1 | | | s02_p2 | | | s04_p3 | | | s06_p4 | | | s07_p6 | | |
|  |  | Mean [%] | SD [%] | n | Mean [%] | SD [%] | n | Mean [%] | SD [%] | n | Mean [%] | SD [%] | n | Mean [%] | SD [%] | n |
| bep | 1.00E-08 | 27.2 | 18.2 | 10 | 0.5 | 2.1 | 6 | 7.9 | 7.6 | 3 | −6.3 | 13.2 | 6 | −3.3 | 4.4 | 14 |
|  | 3.00E-07 | 49.4 | 22.0 | 10 | 5.1 | 6.4 | 6 | 13.7 | 7.9 | 3 | 0.9 | 12.2 | 6 | −0.6 | 13.3 | 14 |
|  | IC_50_ | no inh. |  |  | no inh. |  |  | no inh. |  |  | no inh. |  |  | no inh. |  |  |
|  | n_H_ |  |  |  |  |  |  |  |  |  |  |  |  |  |  |  |
| cpz | 1.00E-07 |  |  |  |  |  |  |  |  |  | −7.9 | 15.6 | 6.0 | −4.1 | 5.5 | 16 |
|  | 3.00E-06 |  |  |  |  |  |  |  |  |  | 15.5 | 12.8 | 6.0 | 56.2 | 17.3 | 16 |
|  | IC_50_ |  |  |  |  |  |  |  |  |  | no inh. |  |  | −5.57 |  |  |
|  | n_H_ |  |  |  |  |  |  |  |  |  |  |  |  | 1.78 |  |  |
| cis | 1.00E-09 | 55.1 | 22.2 | 11 | 0.1 | 1.3 | 5 | 1.4 | 2.1 | 3 | −1.5 | 3.5 | 6 | 3.2 | 6.3 | 12 |
|  | 3.00E-07 | 41.7 | 16.9 | 10 | 4.3 | 4.6 | 5 | 9.8 | 5.6 | 3 | 6.3 | 5.0 | 6 | 12.5 | 14.6 | 12 |
|  | IC_50_ | no inh. |  |  | no inh. |  |  | no inh. |  |  | no inh. |  |  | no inh. |  |  |
|  | n_H_ |  |  |  |  |  |  |  |  |  |  |  |  |  |  |  |
| dil | 3.00E-06 | 57.9 | 12.3 | 11 | 2.3 | 3.1 | 6 | 17.0 | 0.8 | 2 | 8.6 | 6.5 | 6 | 15.0 | 7.3 | 11 |
|  | 1.00E-04 | 99.8 | 7.5 | 11 | 69.5 | 12.6 | 6 | 54.7 | 5.5 | 2 | 91.4 | 2.6 | 6 | 89.9 | 7.1 | 11 |
|  | IC_50_ | −5.64 |  |  | −4.31 |  |  | −4.04 |  |  | −4.70 |  |  | −4.68 |  |  |
|  | n_H_ | 0.90 |  |  | 1.18 |  |  | 0.54 |  |  | 1.26 |  |  | 1.10 |  |  |
| dof | 1.00E-09 | 41.2 | 14.2 | 13 | 2.2 | 3.6 | 5 | 10.2 | 6.8 | 3 | −3.6 | 2.1 | 6 | 1.8 | 5.8 | 13 |
|  | 1.00E-07 | 46.4 | 12.3 | 13 | 5.7 | 11.6 | 5 | 21.3 | 2.2 | 3 | 2.0 | 8.8 | 6 | −2.3 | 10.6 | 13 |
|  | IC_50_ | no inh. |  |  | no inh. |  |  | no inh. |  |  | no inh. |  |  | no inh. |  |  |
|  | n_H_ |  |  |  |  |  |  |  |  |  |  |  |  |  |  |  |
| mex | 1.00E-06 | 57.9 | 17.2 | 13 | −1.5 | 4.3 | 7 | 8.8 | 6.9 | 3 | 2.4 | 5.0 | 6 | 7.1 | 5.3 | 11 |
|  | 1.00E-04 | 89.8 | 14.5 | 12 | 54.9 | 16.0 | 7 | 47.7 | 4.5 | 3 | 62.4 | 10.2 | 6 | 79.8 | 10.4 | 11 |
|  | IC_50_ | −6.45 |  |  | −4.10 |  |  | −3.90 |  |  | −4.16 |  |  | −4.62 |  |  |
|  | n_H_ | 0.31 |  |  | 0.99 |  |  | 0.51 |  |  | 1.28 |  |  | 0.86 |  |  |
| ond | 3.00E-07 | 31.6 | 8.3 | 5 | 1.4 | 3.2 | 6 | 4.4 | 12.2 | 3 | 0.4 | 13.7 | 6 | 1.7 | 10.3 | 16 |
|  | 1.00E-05 | 56.4 | 9.8 | 9 | 9.4 | 9.2 | 6 | 19.5 | 7.2 | 3 | 19.0 | 14.3 | 6 | 17.1 | 21.2 | 16 |
|  | IC_50_ | no inh. |  |  | no inh. |  |  | no inh. |  |  | no inh. |  |  | no inh. |  |  |
|  | n_H_ |  |  |  |  |  |  |  |  |  |  |  |  |  |  |  |
| qui | 1.00E-07 | 51.7 | 16.3 | 11 | −1.3 | 2.8 | 5 | 4.4 | 8.5 | 3 | −4.0 | 8.3 | 6 | −1.4 | 9.9 | 14 |
|  | 1.00E-05 | 72.9 | 17.9 | 10 | 10.3 | 3.1 | 5 | 17.6 | 5.3 | 3 | 33.2 | 4.5 | 6 | 8.9 | 26.0 | 14 |
|  | IC_50_ | −6.40 |  |  | −4.04 |  |  | −4.35 |  |  | −4.68 |  |  | −3.95 |  |  |
|  | n_H_ (=constant) | 1 |  |  | 1 |  |  | 1 |  |  | 1 |  |  | 1 |  |  |
| ran | 3.00E-06 | 32.1 | 11.7 | 9 | 2.0 | 1.9 | 5 | 4.5 | 9.3 | 3 | −0.1 | 16.5 | 6 | 3.1 | 7.4 | 12 |
|  | 1.00E-04 | 86.5 | 17.7 | 11 | 38.7 | 5.3 | 5 | 29.1 | 6.7 | 3 | 53.9 | 13.7 | 5 | 59.2 | 16.9 | 12 |
|  | IC_50_ | −4.90 |  |  | −3.79 |  |  | −3.52 |  |  | −4.08 |  |  | −4.14 |  |  |
|  | n_H_ | 0.77 |  |  | 0.97 |  |  | 0.82 |  |  | 1.08 |  |  | 1.13 |  |  |
| sot | 1.00E-05 | 21.5 | 16.6 | 13 | −1.1 | 5.8 | 7 | 2.4 | 5.5 | 3 | −2.8 | 5.7 | 6 | −4.1 | 10.4 | 10 |
|  | 3.00E-04 | 33.8 | 30.6 | 11 | 1.6 | 9.4 | 7 | −0.6 | 11.5 | 3 | 3.4 | 9.4 | 6 | 0.2 | 11.7 | 10 |
|  | IC_50_ | no inh. |  |  | no inh. |  |  | no inh. |  |  | no inh. |  |  | no inh. |  |  |
|  | n_H_ |  |  |  |  |  |  |  |  |  |  |  |  |  |  |  |
| ter | 1.00E-08 | 31.6 | 21.9 | 9 | −1.3 | 2.7 | 7 | 9.4 | 3.4 | 3 | −4.8 | 18.8 | 6 | −2.1 | 4.7 | 14 |
|  | 3.00E-07 | 40.1 | 17.0 | 13 | 0.0 | 5.6 | 7 | 29.6 | 4.4 | 3 | 3.5 | 8.8 | 6 | −12.8 | 13.2 | 14 |
|  | IC_50_ | no inh. |  |  | no inh. |  |  | no inh. |  |  | no inh. |  |  | no inh. |  |  |
|  | n_H_ |  |  |  |  |  |  |  |  |  |  |  |  |  |  |  |
| ver | 3.00E-08 | 32.7 | 21.5 | 10 | 1.2 | 2.7 | 8 | −3.4 | 4.8 | 3 | −4.4 | 14.1 | 6 | −4.1 | 4.3 | 14 |
|  | 1.00E-06 | 38.3 | 15.3 | 12 | 4.1 | 4.0 | 8 | 0.9 | 3.0 | 3 | 6.1 | 11.3 | 6 | −10.8 | 15.6 | 14 |
|  | IC_50_ | no inh. |  |  | no inh. |  |  | no inh. |  |  | no inh. |  |  | no inh. |  |  |
|  | n_H_ |  |  |  |  |  |  |  |  |  |  |  |  |  |  |  |

^a^bep, bepridil; cpz, chlorpromazine; cis, cisapride; dil, diltiazem; dof, dofetilide; mex, mexiletine; ond, ondansetron; qui, quinidine; ran, ranolazine; sot, sotalol; ter, terfenadine; ver, verapamil.

^b^min/max concentration, minimal and maximal concentration [mol/L]; IC_50_, half-maximal inhibitory concentration [log mol/L]; n_H_, Hill slope.

^c^no inh., no obvious concentration-dependent peak hNav1.5 inhibition and/or no curve fitting performed.

Table S6. Peak hNav1.5 Na^+^ current inhibition (continued)

| Drug^a^ | Min/max concentration^b^ log IC_50_, n_H_ | Site number_platform number^c^ | | | | | | | | | | | | | | | | | |
| --- | --- | --- | --- | --- | --- | --- | --- | --- | --- | --- | --- | --- | --- | --- | --- | --- | --- | --- | --- |
|  |  | s10_p3 | | | s11_p1 | | | s12_p2 | | | s13_p2 | | | s14_p1 | | | s15_p1 | | |
|  |  | Mean [%] | SD [%] | n | Mean [%] | SD [%] | n | Mean [%] | SD [%] | n | Mean [%] | SD [%] | n | Mean [%] | SD [%] | n | Mean [%] | SD [%] | n |
| bep | 1.00E-08 | 3.5 | 7.0 | 4 | 4.3 | 16.2 | 16 | 0.0 | 0.0 | 3 | 3.2 | 5.4 | 3 | 6.0 | 10.3 | 40 | 13.5 | 12.7 | 26 |
|  | 3.00E-07 | 22.0 | 10.8 | 4 | 10.4 | 13.8 | 16 | 0.0 | 0.1 | 3 | 6.7 | 1.1 | 3 | 17.8 | 17.9 | 40 | 51.4 | 19.4 | 25 |
|  | IC_50_ | −5.57 |  |  | no inh. |  |  | no inh. |  |  | no inh. |  |  | −5.61 |  |  | −6.49 |  |  |
|  | n_H_ | 0.57 |  |  |  |  |  |  |  |  |  |  |  | 0.78 |  |  | 0.68 |  |  |
| cpz | 1.00E-07 |  |  |  |  |  |  |  |  |  |  |  |  |  |  |  | 28.9 | 30.8 | 26 |
|  | 3.00E-06 |  |  |  |  |  |  |  |  |  |  |  |  |  |  |  | 99.4 | 8.3 | 25 |
|  | IC_50_ |  |  |  |  |  |  |  |  |  |  |  |  |  |  |  | −6.32 |  |  |
|  | n_H_ |  |  |  |  |  |  |  |  |  |  |  |  |  |  |  | 0.90 |  |  |
| cis | 1.00E-09 | −1.1 | 9.5 | 3 | 8.6 | 10.5 | 16 | −0.2 | 1.3 | 4 | −1.8 | 5.2 | 8 | 1.4 | 11.3 | 38 | 10.3 | 5.0 | 14 |
|  | 3.00E-07 | 11.3 | 5.8 | 3 | 10.0 | 9.4 | 16 | 6.8 | 2.0 | 4 | 6.3 | 5.8 | 8 | −0.4 | 38.0 | 42 | 21.4 | 5.6 | 14 |
|  | IC_50_ | no inh. |  |  | no inh. |  |  | no inh. |  |  | no inh. |  |  | no inh. |  |  | no inh. |  |  |
|  | n_H_ |  |  |  |  |  |  |  |  |  |  |  |  |  |  |  |  |  |  |
| dil | 3.00E-06 | 1.4 | 2.5 | 3 | 17.8 | 11.1 | 16 | 3.4 | 6.3 | 13 | 4.4 | 1.4 | 5 | 19.8 | 14.5 | 39 | 29.1 | 10.7 | 38 |
|  | 1.00E-04 | 64.3 | 8.6 | 3 | 85.9 | 5.9 | 16 | 73.8 | 8.1 | 13 | 75.5 | 7.5 | 5 | 95.2 | 5.6 | 38 | 98.7 | 7.3 | 35 |
|  | IC_50_ | −4.23 |  |  | −4.65 |  |  | −4.37 |  |  | −4.37 |  |  | −4.79 |  |  | −5.05 |  |  |
|  | n_H_ | 1.20 |  |  | 0.92 |  |  | 1.17 |  |  | 1.28 |  |  | 1.09 |  |  | 1.05 |  |  |
| dof | 1.00E-09 | 0.0 | 0.0 | 3 | 13.1 | 16.4 | 15 | −3.3 | 7.9 | 4 | 2.0 | 8.6 | 4 | 5.5 | 13.2 | 43 | 20.4 | 14.9 | 24 |
|  | 1.00E-07 | 16.5 | 4.9 | 3 | 2.8 | 26.4 | 15 | 1.9 | 5.8 | 4 | 2.5 | 6.5 | 4 | −1.2 | 30.4 | 46 | 22.1 | 22.6 | 23 |
|  | IC_50_ | no inh. |  |  | no inh. |  |  | no inh. |  |  | no inh. |  |  | no inh. |  |  | no inh. |  |  |
|  | n_H_ |  |  |  |  |  |  |  |  |  |  |  |  |  |  |  |  |  |  |
| mex | 1.00E-06 | 6.6 | 7.3 | 3 | 10.7 | 13.7 | 15 | 4.9 | 4.1 | 11 | 2.3 | 4.6 | 14 | 6.3 | 15.1 | 44 | 30.6 | 14.3 | 20 |
|  | 1.00E-04 | 53.9 | 7.9 | 3 | 50.8 | 13.8 | 16 | 58.7 | 9.5 | 11 | 50.5 | 13.0 | 14 | 65.7 | 31.9 | 51 | 88.2 | 9.0 | 21 |
|  | IC_50_ | −4.06 |  |  | −3.93 |  |  | −4.18 |  |  | −4.02 |  |  | −4.32 |  |  | −5.18 |  |  |
|  | n_H_ | 0.84 |  |  | 0.56 |  |  | 0.72 |  |  | 0.89 |  |  | 0.84 |  |  | 0.56 |  |  |
| ond | 3.00E-07 | 0.1 | 0.1 | 3 | 6.1 | 11.3 | 16 | 3.0 | 4.1 | 3 | −3.4 | 8.2 | 3 | 3.9 | 10.7 | 33 | 11.8 | 11.3 | 27 |
|  | 1.00E-05 | 20.6 | 8.2 | 3 | 15.2 | 10.2 | 16 | 12.2 | 1.4 | 3 | 7.0 | 4.3 | 3 | 21.3 | 20.2 | 40 | 43.9 | 17.2 | 29 |
|  | IC_50_ | no inh. |  |  | no inh. |  |  | no inh. |  |  | no inh. |  |  | no inh. |  |  | no inh. |  |  |
|  | n_H_ |  |  |  |  |  |  |  |  |  |  |  |  |  |  |  |  |  |  |
| qui | 1.00E-07 | 0.9 | 1.6 | 3 | 12.6 | 11.6 | 16 | 0.1 | 5.4 | 4 | −2.9 | 2.0 | 4 | −0.5 | 22.6 | 40 | 20.3 | 18.7 | 26 |
|  | 1.00E-05 | 25.0 | 4.2 | 3 | 30.0 | 14.9 | 16 | 11.5 | 1.5 | 4 | 8.8 | 6.2 | 4 | 34.5 | 16.7 | 40 | 52.7 | 21.9 | 20 |
|  | IC_50_ | −4.58 |  |  | −4.68 |  |  | −4.11 |  |  | −3.96 |  |  | −4.73 |  |  | −5.39 |  |  |
|  | n_H_ (=constant) | 1 |  |  | 1 |  |  | 1 |  |  | 1 |  |  | 1 |  |  | 1 |  |  |
| ran | 3.00E-06 | 7.0 | 4.8 | 3 | 3.9 | 18.5 | 16 | 1.6 | 4.4 | 6 | 5.1 | 5.3 | 7 | 10.6 | 16.3 | 41 | 19.9 | 8.3 | 13 |
|  | 1.00E-04 | 51.7 | 11.6 | 3 | 54.7 | 14.8 | 16 | 48.2 | 7.0 | 6 | 51.2 | 9.4 | 7 | 63.2 | 19.2 | 46 | 85.7 | 10.5 | 15 |
|  | IC_50_ | −4.05 |  |  | −4.14 |  |  | −3.97 |  |  | −4.02 |  |  | −4.23 |  |  | −4.81 |  |  |
|  | n_H_ | 0.74 |  |  | 0.93 |  |  | 0.98 |  |  | 0.85 |  |  | 0.94 |  |  | 0.85 |  |  |
| sot | 1.00E-05 | 1.7 | 2.0 | 3 | 6.3 | 15.9 | 16 | −0.1 | 1.4 | 4 | 5.0 | 4.3 | 3 | 6.3 | 7.5 | 38 | 15.5 | 10.1 | 15 |
|  | 3.00E-04 | 8.3 | 6.4 | 3 | 17.5 | 18.7 | 15 | 0.4 | 4.8 | 4 | 6.5 | 3.5 | 3 | 3.8 | 14.2 | 43 | 12.9 | 6.5 | 15 |
|  | IC_50_ | no inh. |  |  | no inh. |  |  | no inh. |  |  | no inh. |  |  | no inh. |  |  | no inh. |  |  |
|  | n_H_ |  |  |  |  |  |  |  |  |  |  |  |  |  |  |  |  |  |  |
| ter | 1.00E-08 | −2.6 | 4.4 | 3 | 4.4 | 16.1 | 15 | 0.0 | 3.4 | 7 | 5.1 | 2.8 | 3 | 0.6 | 20.3 | 41 | 14.1 | 25.3 | 27 |
|  | 3.00E-07 | 7.0 | 16.0 | 3 | 3.8 | 12.9 | 16 | 7.5 | 5.0 | 7 | 8.4 | 4.2 | 3 | 13.9 | 25.1 | 41 | 31.7 | 17.2 | 23 |
|  | IC_50_ | no inh. |  |  | no inh. |  |  | no inh. |  |  | no inh. |  |  | no inh. |  |  | no inh. |  |  |
|  | n_H_ |  |  |  |  |  |  |  |  |  |  |  |  |  |  |  |  |  |  |
| ver | 3.00E-08 | 4.5 | 3.7 | 4 | 7.4 | 18.9 | 14 | 2.9 | 1.4 | 3 | −4.9 | 7.9 | 4 | 5.3 | 13.7 | 45 | 17.5 | 14.7 | 27 |
|  | 1.00E-06 | 20.2 | 9.7 | 4 | 7.5 | 9.3 | 16 | 11.4 | 4.2 | 3 | 1.5 | 7.7 | 4 | 12.4 | 15.7 | 43 | 25.6 | 17.1 | 28 |
|  | IC_50_ | no inh. |  |  | no inh. |  |  | no inh. |  |  | no inh. |  |  | no inh. |  |  | no inh. |  |  |
|  | n_H_ |  |  |  |  |  |  |  |  |  |  |  |  |  |  |  |  |  |  |

^a^bep, bepridil; cpz, chlorpromazine; cis, cisapride; dil, diltiazem; dof, dofetilide; mex, mexiletine; ond, ondansetron; qui, quinidine; ran, ranolazine; sot, sotalol; ter, terfenadine; ver, verapamil.

^b^min/max concentration, minimal and maximal concentration [mol/L]; IC_50_, half-maximal inhibitory concentration [log mol/L]; n_H_, Hill slope.

^c^no obvious concentration-dependent peak hNav1.5 inhibition and/or no curve fitting performed.

Table S7. Peak hNav1.5 Na^+^ current inhibition (continued)

| Drug^a^ | Min/max concentration^b^ log IC_50_, n_H_ | Site number_platform number^c^ | | | | | | | | | | | |
| --- | --- | --- | --- | --- | --- | --- | --- | --- | --- | --- | --- | --- | --- |
|  |  | s16_p3 | | | s17_p1 | | | s18_p1 | | | s19_p3 | | |
|  |  | Mean [%] | SD [%] | n | Mean [%] | SD [%] | n | Mean [%] | SD [%] | n | Mean [%] | SD [%] | n |
| bep | 1.00E-08 | 8.1 | 13.1 | 5 | 2.7 | 34.5 | 12 | 54.7 | 8.1 | 3 | 2.2 | 2.1 | 6 |
|  | 3.00E-07 | 26.6 | 33.5 | 5 | 30.0 | 17.5 | 10 | 68.5 | 12.2 | 3 | 17.3 | 6.0 | 6 |
|  | IC_50_ | −5.35 |  |  | −6.17 |  |  | no inh. |  |  | −5.16 |  |  |
|  | n_H_ | 0.36 |  |  | 1.09 |  |  |  |  |  | 0.50 |  |  |
| cpz | 1.00E-07 |  |  |  | 7.0 | 19.2 | 10 |  |  |  |  |  |  |
|  | 3.00E-06 |  |  |  | 90.1 | 5.5 | 7 |  |  |  |  |  |  |
|  | IC_50_ |  |  |  | −6.12 |  |  |  |  |  |  |  |  |
|  | n_H_ |  |  |  | 1.44 |  |  |  |  |  |  |  |  |
| cis | 1.00E-09 | 3.1 | 2.3 | 6 | n/a |  |  | −22.5 | 32.3 | 3 | 6.7 | 2.5 | 5 |
|  | 3.00E-07 | 13.4 | 10.1 | 6 | 1.3 | 6.2 | 9 | 1.0 | 22.7 | 4 | 20.0 | 12.1 | 5 |
|  | IC_50_ | no inh. |  |  | no inh. |  |  | no inh. |  |  | no inh. |  |  |
|  | n_H_ |  |  |  |  |  |  |  |  |  |  |  |  |
| dil | 3.00E-06 | 8.3 | 2.4 | 6 | 18.5 | 10.4 | 11 | 73.5 | 3.8 | 3 | 25.9 | 11.0 | 5 |
|  | 1.00E-04 | 78.4 | 5.8 | 6 | 89.9 | 17.2 | 9 | 97.5 | 8.6 | 6 | 88.0 | 8.2 | 5 |
|  | IC_50_ | −4.47 |  |  | −5.01 |  |  | −6.03 |  |  | −4.89 |  |  |
|  | n_H_ | 1.08 |  |  | 1.34 |  |  | 0.46 |  |  | 0.91 |  |  |
| dof | 1.00E-09 | 19.6 | 29.1 | 7 | 5.6 | 10.6 | 8 | 8.4 | 5.6 | 4 | 7.2 | 3.7 | 6 |
|  | 1.00E-07 | 19.8 | 14.2 | 7 | −20.9 | 14.4 | 8 | −0.9 | 7.9 | 7 | 17.0 | 8.6 | 6 |
|  | IC_50_ | no inh. |  |  | no inh. |  |  | no inh. |  |  | no inh. |  |  |
|  | n_H_ |  |  |  |  |  |  |  |  |  |  |  |  |
| mex | 1.00E-06 | 5.6 | 2.3 | 7 | 10.3 | 7.0 | 9 | 6.1 | 17.9 | 4 | 12.4 | 5.0 | 4 |
|  | 1.00E-04 | 43.0 | 7.8 | 7 | 75.6 | 15.3 | 5 | 83.9 | 11.2 | 6 | 55.6 | 10.6 | 4 |
|  | IC_50_ | −3.81 |  |  | −4.64 |  |  | −4.60 |  |  | −4.21 |  |  |
|  | n_H_ | 0.69 |  |  | 0.83 |  |  | 0.98 |  |  | 0.51 |  |  |
| ond | 3.00E-07 | 2.8 | 2.9 | 7 | −17.0 | 26.5 | 11 | 27.5 | 15.8 | 2 | 3.5 | 4.6 | 4 |
|  | 1.00E-05 | 14.4 | 8.4 | 7 | 33.4 | 11.3 | 12 | 29.7 | 16.2 | 5 | 24.0 | 3.1 | 4 |
|  | IC_50_ | no inh. |  |  | no inh. |  |  | no inh. |  |  | no inh. |  |  |
|  | n_H_ |  |  |  |  |  |  |  |  |  |  |  |  |
| qui | 1.00E-07 | 0.0 | 6.1 | 6 | 11.9 | 8.1 | 6 | −1.7 | 5.9 | 8 | 7.9 | 3.2 | 6 |
|  | 1.00E-05 | 18.5 | 10.9 | 6 | 54.0 | 18.3 | 6 | 35.5 | 9.4 | 9 | 27.1 | 6.7 | 6 |
|  | IC_50_ | −4.39 |  |  | −5.13 |  |  | −4.73 |  |  | −4.64 |  |  |
|  | n_H_ (=constant) | 1 |  |  | 1 |  |  | 1 |  |  | 1 |  |  |
| ran | 3.00E-06 | 8.1 | 3.5 | 6 | 14.2 | 10.0 | 10 | 13.3 | 49.7 | 3 | 10.8 | 2.6 | 7 |
|  | 1.00E-04 | 48.5 | 10.8 | 6 | 86.7 | 3.0 | 4 | 84.6 | 8.3 | 9 | 55.5 | 13.8 | 7 |
|  | IC_50_ | −3.95 |  |  | −4.78 |  |  | −4.45 |  |  | −4.12 |  |  |
|  | n_H_ | 0.70 |  |  | 1.33 |  |  | 1.62 |  |  | 0.76 |  |  |
| sot | 1.00E-05 | 4.8 | 7.7 | 8 | −9.8 | 16.7 | 7 | 55.8 | 26.4 | 3 | 5.6 | 1.4 | 4 |
|  | 3.00E-04 | 21.9 | 33.1 | 8 | −6.3 | 13.3 | 9 | 38.4 | 17.4 | 3 | 19.0 | 3.8 | 4 |
|  | IC_50_ | no inh. |  |  | no inh. |  |  | no inh. |  |  | no inh. |  |  |
|  | n_H_ |  |  |  |  |  |  |  |  |  |  |  |  |
| ter | 1.00E-08 | 4.1 | 1.9 | 7 | −11.2 | 10.1 | 8 | −4.0 | 16.9 | 3 | 5.0 | 3.4 | 5 |
|  | 3.00E-07 | 14.1 | 3.9 | 7 | −4.0 | 15.4 | 9 | 62.4 | 12.0 | 3 | 18.1 | 6.9 | 5 |
|  | IC_50_ | no inh. |  |  | no inh. |  |  | no inh. |  |  | no inh. |  |  |
|  | n_H_ |  |  |  |  |  |  |  |  |  |  |  |  |
| ver | 3.00E-08 | 2.9 | 2.2 | 6 | 13.6 | 21.5 | 11 | −23.1 | 26.2 | 9 | 4.0 | 3.2 | 5 |
|  | 1.00E-06 | 5.5 | 11.2 | 6 | 7.7 | 10.2 | 9 | −5.0 | 12.2 | 4 | 21.9 | 4.6 | 5 |
|  | IC_50_ | no inh. |  |  | no inh. |  |  | no inh. |  |  | no inh. |  |  |
|  | n_H_ |  |  |  |  |  |  |  |  |  |  |  |  |

^a^bep, bepridil; cpz, chlorpromazine; cis, cisapride; dil, diltiazem; dof, dofetilide; mex, mexiletine; ond, ondansetron; qui, quinidine; ran, ranolazine; sot, sotalol; ter, terfenadine; ver, verapamil.

^b^min/max concentration, minimal and maximal concentration [mol/L]; IC_50_, half-maximal inhibitory concentration [log mol/L]; n_H_, Hill slope.

^c^Please note that s20_m used concentrations different from those in column 2. no inh., no obvious concentration-dependent peak hNav1.5 inhibition and/or no curve fitting performed.

Table S8. Late hNav1.5 Na^+^ current inhibition

| Drug^a^ | Min/max concentration^b^ log IC_50_, n_H_ | Site number_platform number^c^ | | | | | | | | | | | |
| --- | --- | --- | --- | --- | --- | --- | --- | --- | --- | --- | --- | --- | --- |
|  |  | s06_p4 |  |  | s07_p6 |  |  | s13_p2 |  |  | s14_p1 |  |  |
|  |  | Mean [%] | SD [%] | n | Mean [%] | SD [%] | n | Mean [%] | SD [%] | n | Mean [%] | SD [%] | n |
| bep | 1.00E-08 | −0.5 | 7.3 | 6 | 5.5 | 8.7 | 14 | 24.3 | 7.0 | 2 | 54.2 | 5.1 | 9 |
|  | 3.00E-07 | 43.7 | 10.3 | 6 | 12.3 | 22.9 | 14 | 29.9 | 16.2 | 5 | 10.2 | 22.6 | 12 |
|  | IC_50_ | −6.44 |  |  | no inh. |  |  | no inh. |  |  | no inh. |  |  |
|  | n_H_ | 1.86 |  |  |  |  |  |  |  |  |  |  |  |
| cpz | 1.00E-07 | 1.6 | 9.0 | 6 | 14.4 | 10.6 | 12 |  |  |  |  |  |  |
|  | 3.00E-06 | 94.4 | 2.0 | 6 | 69.2 | 7.7 | 12 |  |  |  |  |  |  |
|  | IC_50_ | −6.14 |  |  | −5.78 |  |  |  |  |  |  |  |  |
|  | n_H_ | 1.74 |  |  | 1.11 |  |  |  |  |  |  |  |  |
| cis | 1.00E-09 | −0.7 | 7.0 | 6 | 4.3 | 13.0 | 12 | −16.7 | 51.5 | 6 | −6.9 | 14.9 | 9 |
|  | 3.00E-07 | 31.8 | 5.9 | 6 | −17.0 | 13.6 | 12 | 7.2 | 6.4 | 7 | −6.3 | 31.3 | 13 |
|  | IC_50_ | no inh. |  |  | no inh. |  |  | no inh. |  |  | no inh. |  |  |
|  | n_H_ |  |  |  |  |  |  |  |  |  |  |  |  |
| dil | 3.00E-06 | 48.7 | 4.6 | 6 | −5.2 | 17.7 | 12 | 2.5 | 3.2 | 4 | 36.7 | 10.7 | 11 |
|  | 1.00E-04 | 95.4 | 4.0 | 6 | 78.8 | 12.6 | 12 | 56.6 | 11.3 | 5 | 91.7 | 8.0 | 11 |
|  | IC_50_ | −5.50 |  |  | −4.38 |  |  | −4.06 |  |  | −5.26 |  |  |
|  | n_H_ | 1.11 |  |  | 1.55 |  |  | 2.00 |  |  | 1.00 |  |  |
| dof | 1.00E-09 | 7.0 | 15.7 | 6 | 10.1 | 15.1 | 12 | −6.9 | 26.7 | 2 | −44.8 | 48.5 | 13 |
|  | 1.00E-07 | 6.0 | 16.8 | 6 | −19.7 | 15.9 | 12 | 9.7 | 10.0 | 4 | −41.0 | 37.4 | 15 |
|  | IC_50_ | no inh. |  |  | no inh. |  |  | no inh. |  |  | no inh. |  |  |
|  | n_H_ |  |  |  |  |  |  |  |  |  |  |  |  |
| mex | 1.00E-06 | 18.9 | 6.7 | 6 | 5.5 | 13.4 | 15 | 26.4 | 8.7 | 5 | −15.4 | 36.6 | 14 |
|  | 1.00E-04 | 96.2 | 3.7 | 6 | 89.6 | 8.1 | 15 | 76.8 | 13.7 | 4 | 98.8 | 10.3 | 20 |
|  | IC_50_ | −5.33 |  |  | −4.79 |  |  | −5.16 |  |  | −4.96 |  |  |
|  | n_H_ | 0.97 |  |  | 1.16 |  |  | 0.53 |  |  | 4.08 |  |  |
| ond | 3.00E-07 | 0.5 | 11.6 | 6 | −14.8 | 24.0 | 14 | 9.2 | 13.9 | 4 | 20.2 | 14.3 | 3 |
|  | 1.00E-05 | 61.7 | 8.9 | 6 | 47.6 | 15.9 | 14 | 20.9 | 4.8 | 4 | 40.9 | 26.2 | 12 |
|  | IC_50_ | −5.16 |  |  | −4.99 |  |  | no inh. |  |  | no inh. |  |  |
|  | n_H_ | 1.28 |  |  | 1.57 |  |  |  |  |  |  |  |  |
| qui | 1.00E-07 | 4.2 | 13.4 | 6 | 8.2 | 9.8 | 12 | −1.9 | 7.3 | 8 | −19.2 | 26.6 | 10 |
|  | 1.00E-05 | 78.1 | 7.6 | 6 | 46.2 | 7.8 | 12 | 15.9 | 21.1 | 7 | 68.8 | 14.2 | 11 |
|  | IC_50_ | −5.63 |  |  | −4.90 |  |  | −3.86 |  |  | −5.15 |  |  |
|  | n_H_ | 0.91 |  |  | 0.74 |  |  | 0.63 |  |  | 2.00 |  |  |
| ran | 3.00E-06 | 33.8 | 7.2 | 6 | 2.6 | 11.0 | 15 | 0.8 | 13.9 | 5 | 23.0 | 18.6 | 14 |
|  | 1.00E-04 | 93.8 | 2.3 | 5 | 78.8 | 8.0 | 15 | 68.4 | 12.0 | 3 | 93.6 | 11.0 | 16 |
|  | IC_50_ | −5.21 |  |  | −4.45 |  |  | −4.58 |  |  | −5.03 |  |  |
|  | n_H_ | 1.00 |  |  | 1.33 |  |  | 0.99 |  |  | 1.00 |  |  |
| sot | 1.00E-05 | −0.6 | 11.4 | 6 | −5.4 | 8.4 | 16 | −7.2 | 10.5 | 3 | −28.8 | 33.2 | 8 |
|  | 3.00E-04 | −0.9 | 5.3 | 6 | 1.7 | 24.8 | 16 | −2.0 | 22.4 | 4 | −21.5 | 19.1 | 12 |
|  | IC_50_ | no inh. |  |  | no inh. |  |  | no inh. |  |  | no inh. |  |  |
|  | n_H_ |  |  |  |  |  |  |  |  |  |  |  |  |
| ter | 1.00E-08 | 5.3 | 14.1 | 5 | 22.9 | 19.2 | 12 | 3.3 | 11.5 | 4 | 14.8 | 13.2 | 11 |
|  | 3.00E-07 | 78.3 | 6.7 | 6 | 36.2 | 13.9 | 12 | 35.7 | 9.2 | 3 | 58.5 | 6.6 | 12 |
|  | IC_50_ | −6.97 |  |  | no inh. |  |  | −6.12 |  |  | −6.57 |  |  |
|  | n_H_ | 1.14 |  |  |  |  |  | 0.65 |  |  | 0.84 |  |  |
| ver | 3.00E-08 | −1.3 | 12.5 | 6 | 7.5 | 13.3 | 15 | 13.9 | 15.4 | 5 | 19.3 | 13.2 | 13 |
|  | 1.00E-06 | 49.4 | 10.8 | 6 | −7.8 | 20.1 | 15 | 26.2 | 6.9 | 5 | 31.3 | 11.6 | 13 |
|  | IC_50_ | −6.01 |  |  | no inh. |  |  | no inh. |  |  | no inh. |  |  |
|  | n_H_ | 1.23 |  |  |  |  |  |  |  |  |  |  |  |

^a^bep, bepridil; cpz, chlorpromazine; cis, cisapride; dil, diltiazem; dof, dofetilide; mex, mexiletine; ond, ondansetron; qui, quinidine; ran, ranolazine; sot, sotalol; ter, terfenadine; ver, verapamil.

^b^min/max concentration, minimal and maximal concentration [mol/L]; IC_50_, half-maximal inhibitory concentration [log mol/L]; n_H_, Hill slope.

^c^Please note that s20_m used concentrations different from those in column 2. no inh., no obvious concentration-dependent late hNav1.5 inhibition and/or no curve fitting performed.

Table S9. Peak hCav1.2 Ca^2+^ current inhibition

| Drug^a^ | Min/max concentration^b^ log IC_50_, n_H_ | Site number_platform number^c^ | | | | | | | | | | | | | | | | | |
| --- | --- | --- | --- | --- | --- | --- | --- | --- | --- | --- | --- | --- | --- | --- | --- | --- | --- | --- | --- |
|  |  | s01_p1 | | | s02_p2^d^ | | | s04_p3 | | | s06_p4 | | | s11_p1 | | | s14_p1 | | |
|  |  | Mean [%] | SD [%] | n | Mean [%] | SD [%] | n | Mean [%] | SD [%] | n | Mean [%] | SD [%] | n | Mean [%] | SD [%] | n | Mean [%] | SD [%] | n |
| bep | 1.00E-08 | 5.6 | 11.0 | 8 | 4.4 | 7.2 | 4 | 12.6 | 1.3 | 3 | 5.9 | 21.9 | 6 | 13.2 | 27.1 | 15 | −0.1 | 23.9 | 31 |
|  | 3.00E-07 | 16.7 | 13.8 | 8 | 34.4 | 12.4 | 4 | 17.5 | 5.8 | 3 | 13.9 | 25.4 | 5 | 21.4 | 14.6 | 8 | 8.2 | 14.6 | 30 |
|  | IC_50_ | no inh. |  |  | −6.08 |  |  | no inh. |  |  | no inh. |  |  | no inh. |  |  | no inh. |  |  |
|  | n_H_ |  |  |  | 0.64 |  |  |  |  |  |  |  |  |  |  |  |  |  |  |
| cpz | 1.00E-07 |  |  |  |  |  |  |  |  |  |  |  |  |  |  |  |  |  |  |
|  | 3.00E-06 |  |  |  |  |  |  |  |  |  |  |  |  |  |  |  |  |  |  |
|  | IC_50_ |  |  |  |  |  |  |  |  |  |  |  |  |  |  |  |  |  |  |
|  | n_H_ |  |  |  |  |  |  |  |  |  |  |  |  |  |  |  |  |  |  |
| cis | 1.00E-09 | 7.4 | 14.4 | 13 | −5.7 | 8.2 | 3 |  |  |  | −8.1 | 14.0 | 4 | 7.8 | 29.0 | 14 | −10.3 | 14.3 | 21 |
|  | 3.00E-07 | 8.1 | 6.1 | 10 | 14.5 | 6.6 | 3 |  |  |  | −6.0 | 22.6 | 5 | 10.4 | 21.3 | 14 | −19.9 | 25.6 | 29 |
|  | IC_50_ | no inh. |  |  | no inh. |  |  |  |  |  | no inh. |  |  | no inh. |  |  | no inh. |  |  |
|  | n_H_ |  |  |  |  |  |  |  |  |  |  |  |  |  |  |  |  |  |  |
| dil | 3.00E-06 | 72.7 | 11.7 | 10 | 17.3 | 15.1 | 5 |  |  |  | 13.4 | 18.7 | 6 | 38.7 | 27.3 | 14 | 32.1 | 18.4 | 24 |
|  | 1.00E-04 | 98.6 | 7.8 | 10 | 82.9 | 7.2 | 5 |  |  |  | 85.6 | 11.7 | 6 | 65.9 | 22.1 | 12 | 98.9 | 7.4 | 28 |
|  | IC_50_ | −6.29 |  |  | −4.70 |  |  |  |  |  | −4.50 |  |  | −4.65 |  |  | −5.14 |  |  |
|  | n_H_ | 0.52 |  |  | 0.90 |  |  |  |  |  | 1.17 |  |  | 0.30 |  |  | 0.95 |  |  |
| dof | 1.00E-09 | 5.2 | 11.2 | 14 | 10.2 | 9.8 | 3 |  |  |  | −5.6 | 18.6 | 6 | 5.0 | 18.6 | 13 | −13.5 | 21.0 | 27 |
|  | 1.00E-07 | 8.1 | 4.7 | 10 | 19.1 | 15.9 | 3 |  |  |  | −3.1 | 15.4 | 4 | 12.3 | 25.2 | 16 | −38.0 | 45.0 | 25 |
|  | IC_50_ | no inh. |  |  | no inh. |  |  |  |  |  | no inh. |  |  | no inh. |  |  | no inh. |  |  |
|  | n_H_ |  |  |  |  |  |  |  |  |  |  |  |  |  |  |  |  |  |  |
| mex | 1.00E-06 | 10.1 | 3.5 | 14 | −11.1 | 8.9 | 3 |  |  |  | 0.5 | 10.5 | 6 | 12.9 | 22.1 | 14 | −17.7 | 16.3 | 26 |
|  | 1.00E-04 | 34.0 | 8.2 | 16 | 1.8 | 11.5 | 3 |  |  |  | 39.1 | 25.7 | 6 | 22.9 | 22.1 | 14 | 16.2 | 24.2 | 21 |
|  | IC_50_ | −3.43 |  |  | no inh. |  |  |  |  |  | −3.75 |  |  | no inh. |  |  | −3.63 |  |  |
|  | n_H_ | 0.60 |  |  |  |  |  |  |  |  | 0.87 |  |  |  |  |  | 2.00 |  |  |
| ond | 3.00E-07 | 3.2 | 10.3 | 11 | −6.3 | 20.3 | 4 | 2.6 | 4.5 | 2 | 18.9 | 7.5 | 4 | 19.7 | 20.5 | 15 | 4.0 | 13.4 | 31 |
|  | 1.00E-05 | 9.7 | 4.7 | 6 | 4.3 | 27.0 | 4 | 16.1 | 1.7 | 2 | 18.6 | 17.9 | 6 | 14.7 | 23.7 | 12 | 11.2 | 7.1 | 28 |
|  | IC_50_ | no inh. |  |  | no inh. |  |  | −3.81 |  |  | no inh. |  |  | no inh. |  |  | −4.54 |  |  |
|  | n_H_ |  |  |  |  |  |  | 0.60 |  |  |  |  |  |  |  |  | 2.00 |  |  |
| qui | 1.00E-07 | 10.2 | 7.4 | 13 | −5.3 | 1.3 | 3 |  |  |  | −0.5 | 19.6 | 6 | 10.2 | 18.2 | 12 | −21.4 | 28.6 | 25 |
|  | 1.00E-05 | 22.4 | 11.8 | 12 | 3.6 | 8.1 | 3 |  |  |  | 10.0 | 11.9 | 6 | 10.4 | 18.3 | 13 | 4.3 | 14.2 | 25 |
|  | IC_50_ | no inh. |  |  | no inh. |  |  |  |  |  | no inh. |  |  | no inh. |  |  | no inh. |  |  |
|  | n_H_ |  |  |  |  |  |  |  |  |  |  |  |  |  |  |  |  |  |  |
| ran | 3.00E-06 | −2.7 | 14.7 | 11 | −1.8 | 4.8 | 4 |  |  |  | 12.9 | 21.7 | 6 | 14.1 | 16.0 | 15 | −1.7 | 18.2 | 29 |
|  | 1.00E-04 | 9.0 | 13.7 | 12 | 22.9 | 16.6 | 4 |  |  |  | 23.0 | 25.2 | 4 | 33.0 | 28.0 | 14 | 21.7 | 9.8 | 30 |
|  | IC_50_ | no inh. |  |  | −3.30 |  |  |  |  |  | −3.73 |  |  | −2.87 |  |  | −3.61 |  |  |
|  | n_H_ |  |  |  | 0.74 |  |  |  |  |  | 2.00 |  |  | 0.32 |  |  | 1.42 |  |  |
| sot | 1.00E-05 | 2.9 | 13.1 | 10 | 1.6 | 10.3 | 5 |  |  |  | −5.7 | 20.2 | 6 | n/a |  |  | −21.2 | 24.8 | 23 |
|  | 3.00E-04 | 14.3 | 9.2 | 7 | 2.8 | 13.1 | 5 |  |  |  | 1.5 | 19.7 | 6 | 15.8 | 25.2 | 13 | −25.8 | 46.3 | 30 |
|  | IC_50_ | no inh. |  |  | no inh. |  |  |  |  |  | no inh. |  |  | no inh. |  |  | no inh. |  |  |
|  | n_H_ |  |  |  |  |  |  |  |  |  |  |  |  |  |  |  |  |  |  |
| ter | 1.00E-08 | −7.1 | 13.4 | 11 | −2.4 | 14.3 | 6 | 10.1 | 5.2 | 3 | 2.7 | 18.0 | 6 | 14.2 | 21.1 | 12 | 5.8 | 12.4 | 30 |
|  | 3.00E-07 | 35.6 | 11.2 | 10 | 74.7 | 12.7 | 8 | 32.9 | 5.6 | 3 | 10.2 | 12.3 | 6 | 16.8 | 28.8 | 15 | 7.4 | 13.5 | 31 |
|  | IC_50_ | −6.35 |  |  | −6.83 |  |  | −5.74 |  |  | no inh. |  |  | no inh. |  |  | no inh. |  |  |
|  | n_H_ | 1.62 |  |  | 1.40 |  |  | 0.41 |  |  |  |  |  |  |  |  |  |  |  |
| ver | 3.00E-08 | 3.2 | 8.2 | 9 | 6.2 | 20.6 | 5 | 7.9 | 2.6 | 3 | 8.1 | 11.7 | 6 | 11.7 | 20.1 | 14 | 8.7 | 11.0 | 31 |
|  | 1.00E-06 | 42.4 | 19.4 | 12 | 34.4 | 35.4 | 5 | 22.1 | 7.0 | 3 | 24.2 | 23.0 | 6 | 39.3 | 16.5 | 16 | 35.3 | 13.9 | 31 |
|  | IC_50_ | −5.88 |  |  | fnc |  |  | −4.65 |  |  | −4.86 |  |  | −5.66 |  |  | −5.63 |  |  |
|  | n_H_ | 1.16 |  |  |  |  |  | 0.41 |  |  | 0.48 |  |  | 0.52 |  |  | 0.73 |  |  |

^a^bep, bepridil; cpz, chlorpromazine; cis, cisapride; dil, diltiazem; dof, dofetilide; mex, mexiletine; ond, ondansetron; qui, quinidine; ran, ranolazine; sot, sotalol; ter, terfenadine; ver, verapamil.

^b^min/max conc., minimal and maximal concentration [mol/L]; IC_50_, half-maximal inhibitory concentration [log mol/L]; n_H_, Hill slope.

^c^Please note that s20_m used concentrations different from those in column 2. fnc, fit did not converge; no inh., no obvious concentration-dependent late hNav1.5 inhibition and/or no curve fitting performed.

^d^Please note that data from s02_p2 were corrected for rundown.

Table S10. Peak hCav1.2 Ca^2+^ current inhibition (continued)

| Drug^a^ | Min/max concentration^b^ log IC_50_, n_H_ | Site number_platform number^c^ | | | | | |
| --- | --- | --- | --- | --- | --- | --- | --- |
|  |  | s16_p3 | | | s18_p1 | | |
|  |  | Mean [%] | SD [%] | n | Mean [%] | SD [%] | n |
| bep | 1.00E-08 | 8.6 | 10.2 | 3 | 28.2 | 18.5 | 5 |
|  | 3.00E-07 | 45.8 | 2.8 | 3 | 31.3 | 3.4 | 3 |
|  | IC_50_ | −6.52 |  |  | fnc |  |  |
|  | n_H_ | 0.51 |  |  |  |  |  |
| cpz | 1.00E-07 |  |  |  |  |  |  |
|  | 3.00E-06 |  |  |  |  |  |  |
|  | IC_50_ |  |  |  |  |  |  |
|  | n_H_ |  |  |  |  |  |  |
| cis | 1.00E-09 | 20.1 | 11.3 | 4 | 2.8 | 22.7 | 6 |
|  | 3.00E-07 | 49.1 | 10.9 | 4 | −16.5 | 15.0 | 2 |
|  | IC_50_ | −6.35 |  |  | no inh. |  |  |
|  | n_H_ | 0.24 |  |  |  |  |  |
| dil | 3.00E-06 | 7.3 | 14.6 | 3 | 69.2 | 18.8 | 6 |
|  | 1.00E-04 | 0.3 | 19.6 | 3 | 102.9 | 15.1 | 7 |
|  | IC_50_ | no inh. |  |  | −5.96 |  |  |
|  | n_H_ |  |  |  | 0.71 |  |  |
| dof | 1.00E-09 | 15.1 | 9.7 | 3 | −38.9 | 42.8 | 3 |
|  | 1.00E-07 | 54.6 | 4.8 | 3 | 2.6 | 46.7 | 3 |
|  | IC_50_ | 7.32 |  |  | no inh. |  |  |
|  | n_H_ | 0.38 |  |  |  |  |  |
| mex | 1.00E-06 | −0.3 | 18.8 | 3 | 2.3 | 28.7 | 9 |
|  | 1.00E-04 | 31.7 | 28.8 | 3 | 35.7 | 8.5 | 6 |
|  | IC_50_ | −3.60 |  |  | −3.79 |  |  |
|  | n_H_ | 0.80 |  |  | 1.07 |  |  |
| ond | 3.00E-07 | 20.6 | 2.6 | 4 | 16.9 | 19.0 | 3 |
|  | 1.00E-05 | 54.1 | 13.7 | 4 | 4.9 | 22.7 | 3 |
|  | IC_50_ | −5.18 |  |  | no inh. |  |  |
|  | n_H_ | 0.42 |  |  |  |  |  |
| qui | 1.00E-07 | 14.0 | 5.1 | 3 | 13.7 | 80.0 | 3 |
|  | 1.00E-05 | 55.9 | 7.6 | 3 | 60.0 | 56.0 | 4 |
|  | IC_50_ | −5.24 |  |  | −5.08 |  |  |
|  | n_H_ | 0.46 |  |  | 2.00 |  |  |
| ran | 3.00E-06 | 16.5 | 3.0 | 3 | −14.2 | 20.0 | 10 |
|  | 1.00E-04 | 31.8 | 3.6 | 3 | 36.1 | 36.9 | 8 |
|  | IC_50_ | −2.51 |  |  | −3.83 |  |  |
|  | n_H_ | 0.24 |  |  | 2.00 |  |  |
| sot | 1.00E-05 | 1.5 | 32.9 | 4 | 14.9 | 25.5 | 3 |
|  | 3.00E-04 | 43.7 | 29.4 | 4 | 14.7 | 7.9 | 3 |
|  | IC_50_ | −3.41 |  |  | no inh. |  |  |
|  | n_H_ | 0.63 |  |  |  |  |  |
| ter | 1.00E-08 | 8.1 | 14.5 | 4 | −7.0 | 34.9 | 8 |
|  | 3.00E-07 | 52.1 | 16.2 | 4 | 56.4 | 5.8 | 3 |
|  | IC_50_ | −6.56 |  |  | −6.54 |  |  |
|  | n_H_ | 0.65 |  |  | 2.00 |  |  |
| ver | 3.00E-08 | 15.7 | 10.6 | 3 | −24.2 | 11.7 | 4 |
|  | 1.00E-06 | 49.5 | 7.5 | 3 | 100.1 | 36.6 | 4 |
|  | IC_50_ | −6.22 |  |  | −6.47 |  |  |
|  | n_H_ | 1.40 |  |  | 1.40 |  |  |

^a^bep, bepridil; cpz, chlorpromazine; cis, cisapride; dil, diltiazem; dof, dofetilide; mex, mexiletine; ond, ondansetron; qui, quinidine; ran, ranolazine; sot, sotalol; ter, terfenadine; ver, verapamil.

^b^min/max concentration, minimal and maximal concentration [mol/L]; IC_50_, half-maximal inhibitory concentration [log mol/L]; n_H_, Hill slope.

^c^Please note that s20_m used concentrations different from those in column 2. fnc, fit did not converge; no inh., no obvious concentration-dependent peak hCav1.2 inhibition and/or no curve fitting performed.

^d^Please note that data from s02_p2 were corrected for rundown.

Table S11. Survey results comparing experimental conditions from 10 responding sites

| Experimental condition | 10 sites (4 platforms), 10 machines |
| --- | --- |
| Solutions used as per protocol: no, yes, mixed | No = 5, yes = 4, mixed = 1 |
| CiPA voltage clamp protocols | Yes = 9 (1 at 0.2 Hz) |
| Solution preparation: vortexed (V), heated (H), sonicated (S), none (N) | V = 7; V,H,S = 1; V,S = 1; N = 1 |
| Drug storage: glass (G), plastic (P), Teflon (T) | G = 9, P = 1 |
| Compound plate glass (G), plastic (P), Teflon (T) | G = 6, P = 1, T = 3 |
| Additions: single (S) or multiple (M) (with count) | S = 5, M = 1, M(×2 = 2), M(×3 = 1) |
| Single (S) or ascending (A) dosing | S = 5, A = 5 |
| Exposure time (min) | 3 min = 3, 4 min = 1, ≥4 min = 1, 5 min = 2, ≥5 min = 1, 3 variable (4.2–6.7, 5.7–8, and 1.4–2.9 min) |
| Rundown correction: no, yes | No = 9, Yes = 1, Yes = 1 (peak INa only) |
| Seal resistance | ≥200 mOhm, 4; >300 mOhm, 1; >500 mOhm, 1; >gigohm, 3; 1 = gigohm for hNav1.5 only |
| Leak: monitored (M), stable (S), subtracted (sub) | M = 5; S = 2; M,S = 2; 1M < 10%; 1M (with failed hNav1.5) |

Supplementary Figure Legends

Figure S1.

Concentration-response curves for hKv11.1/hERG current inhibition. Concentration-response curves were fitted to the means values per test compound and per test site with a standard four-parameter logistic equation (constraints: min = 0; max = 100; nH < 2). Platforms used are color-coded: p1, blue; p2, green; p3, orange; p4, black; and p5, black. Automated assays (s01–s19) were conducted at room temperature (open symbols, dashed lines) and at 35°C (filled symbols, solid lines). Symbols are identical to Figure 1; IC50 values are listed in Supplementary Tables S3–S5.

Figure S2.

Concentration-response curves for peak hCav1.2 Ca2+ current inhibition. Concentration-response curves were fitted to the mean values per test compound and test site with a standard four-parameter logistic equation (constraints: min = 0; max = 100; nH < 2). Platforms used are color-coded: p1, blue; p2, green; p3, orange; p4, black; and p5, black. Automated assays (s01–s19) were conduct at room temperature (open symbols, dashed lines) and at 35°C (filled symbols, solid lines). Symbols: see Figure 3; IC50 values: Supplementary Tables S10–S11.

Figure S3.

Peak hCav1.2 Ca^2+^ current IC_50_ concentrations per compound. Graphical representation of peak hCav1.2 current inhibition by the 12 test compounds: spread of log-IC_50_ concentrations per compound (compare Supplementary Tables S9–S10) with superimposed box-and-whiskers plot (median, 25/75 percentile, min/max values); the number of data points from automated platforms is indicated at the bottom of each column. Platforms used are color-coded: p1, blue; p2, green; p3, orange; p4, black; and p5, black. Automated assays (s01–s18) were conducted at room temperature (open symbols) and at 35°C (filled 9 symbols). Automated assays (s01–s19) were conducted at room temperature (open symbols) and at 35°C (filled symbols).

Figure S4.

Variability of peak hNav1.5 Na^+^ current inhibition at NOEL/LOEL concentrations per site. Test compound concentrations were chosen in such a manner that the lowest concentration for each compound was known/expected to cause no or only a minor inhibition of the peak hNav1.5 Na**+** current. Therefore, at these NOEL/LOEL concentrations, current inhibition values are expected to cluster around zero percent. Inhibition mean values (and SD) at the lowest test compound concentration were averaged per site over all 12 test compounds. Platforms used are color-coded: p1, blue; p2, green; p3, orange; p4, black; and p5, black. Automated assays (s01–s19) were conducted at room temperature (open symbols) and at 35°C (filled symbols).

Figure S5.

Concentration-response curves for peak hNav1.5 Na^+^ current inhibition Concentration-response curves were fitted to the means values per test compound and per test site with a standard four-parameter logistic equation (constraints: min = 0; max = 100; n**H** < 2). Platforms used are color-coded: p1, blue; p2, green; p3, orange; p4, black; and p5, black. Automated assays (s01–s19) were conducted at room temperature (open symbols, dashed lines) and at 35°C (filled symbols, solid lines). Symbols are identical to Figure S3; IC**50** values are listed in Supplementary Tables S6–S8.

|  |
| --- |

Figure S6.

Late hNav1.5 Na^+^ current IC_50_ concentrations per compound. Graphical representation of late hNav1.5 Na^+^ current inhibition by the 12 test compounds: spread of individual log-IC_50_ concentrations per compound and per site (compare Supplementary Table S9) with superimposed box-and-whiskers plot (median, 25/75 percentile, min/max values); the number of data points from automated platforms is indicated at the bottom of each column. Platforms used are color-coded: p1, blue; p2, green; p3, orange; p4, black; and p5, black. Automated assays (s01–s19) were conducted at room temperature (open symbols) and at 35°C (filled symbols).

Figure S7.

Concentration-response curves for late hNav1.5 Na^+^ current inhibition. Concentration-response curves were fitted to the means values per test compound and per test site with a standard four-parameter logistic equation (constraints: min = 0; max = 100; n**H** < 2). Platforms used are color-coded: p1, blue; p2, green; p3, orange; p4, black; and p5, black. Automated assays (s01–s19) were conducted at room temperature (open symbols, dashed lines) and at 35°C (filled symbols, solid lines). Symbols are identical to Supplementary Figure S6; IC**50** values are listed in Supplementary Table S9.

Figure S8.

Variability of Block of Late hNav1.5. Plotted are IC_50_ values for block by each drug evaluated on four different platforms (P1, P2, P4, and P6, left to right). Lowest column values represent no data reported. Second lowest values plotted represent no inhibition reported.

Figure S9.

Variability of Block of hCav1.2. Plotted are IC_50_ values for block by each drug evaluated on four different platforms across 8 sites (according to sequence s01p1, s11p1, s14p1, s18p1, s02p2, s04p3, s16p3, s06p4). Lowest column values represent no data reported. Second lowest values plotted represent no inhibition reported.
