# Supplementary figures and images for: Cross-site and cross-platform variability of automated patch clamp assessments of drug effects on human cardiac currents in recombinant cells
Source: Sci Rep. 2020 Mar 27;10:5627. doi: 10.1038/s41598-020-62344-w (PMC7101356; doi:10.1038/s41598-020-62344-w)

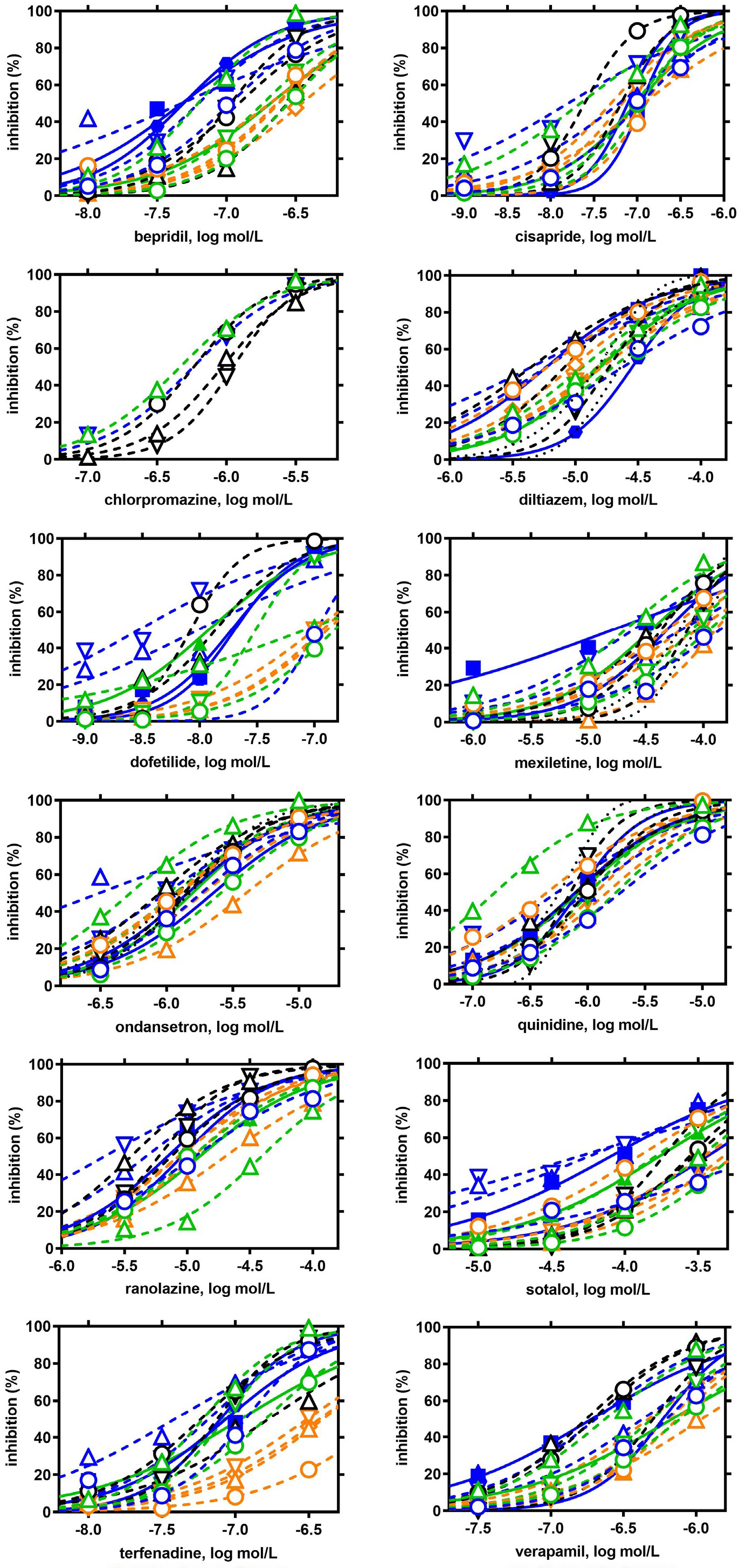

Supplement: Supplementary file 2 — Supporting Information2. [file 41598_2020_62344_MOESM2_ESM.tiff]

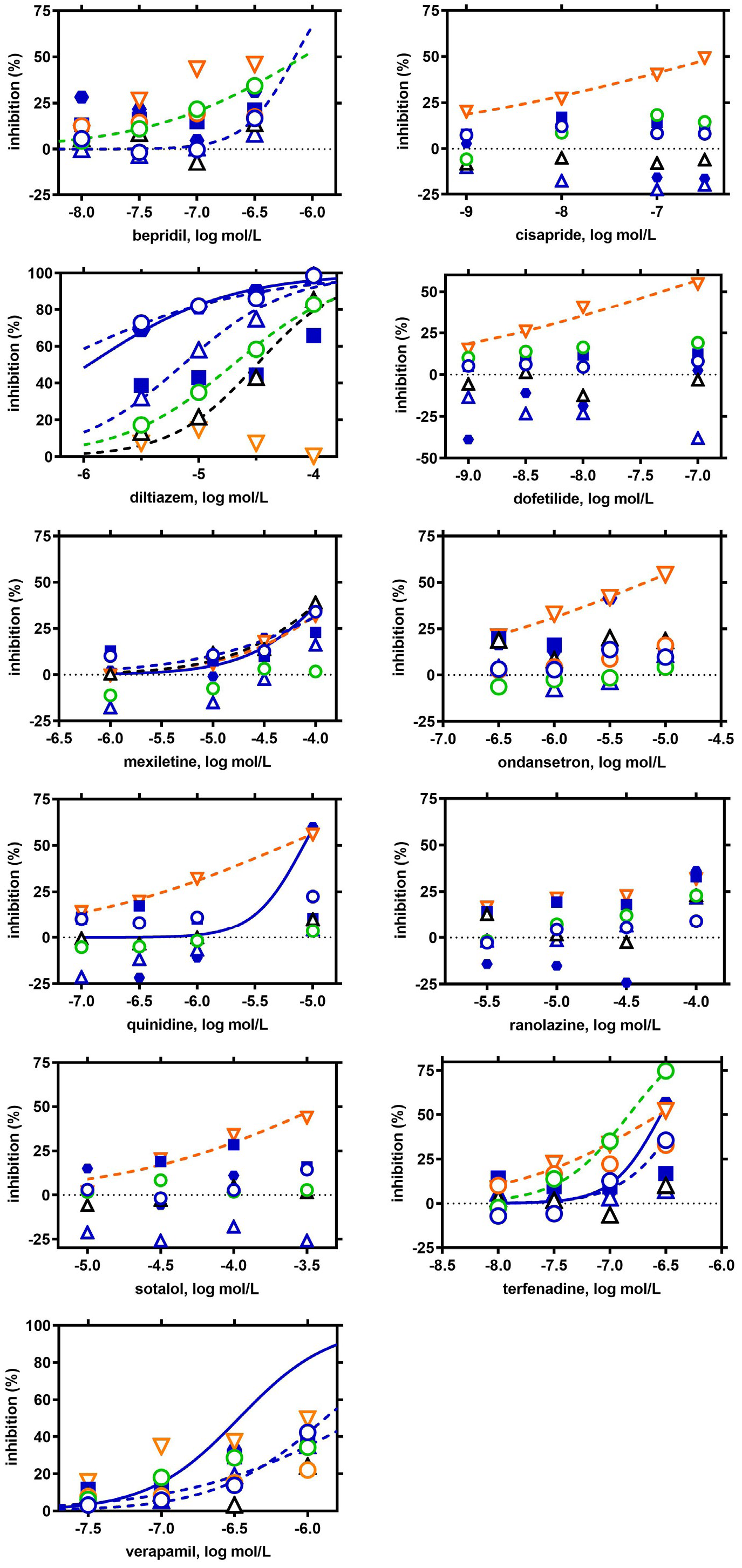

Supplement: Supplementary file 3 — Supporting Information3. [file 41598_2020_62344_MOESM3_ESM.tiff]

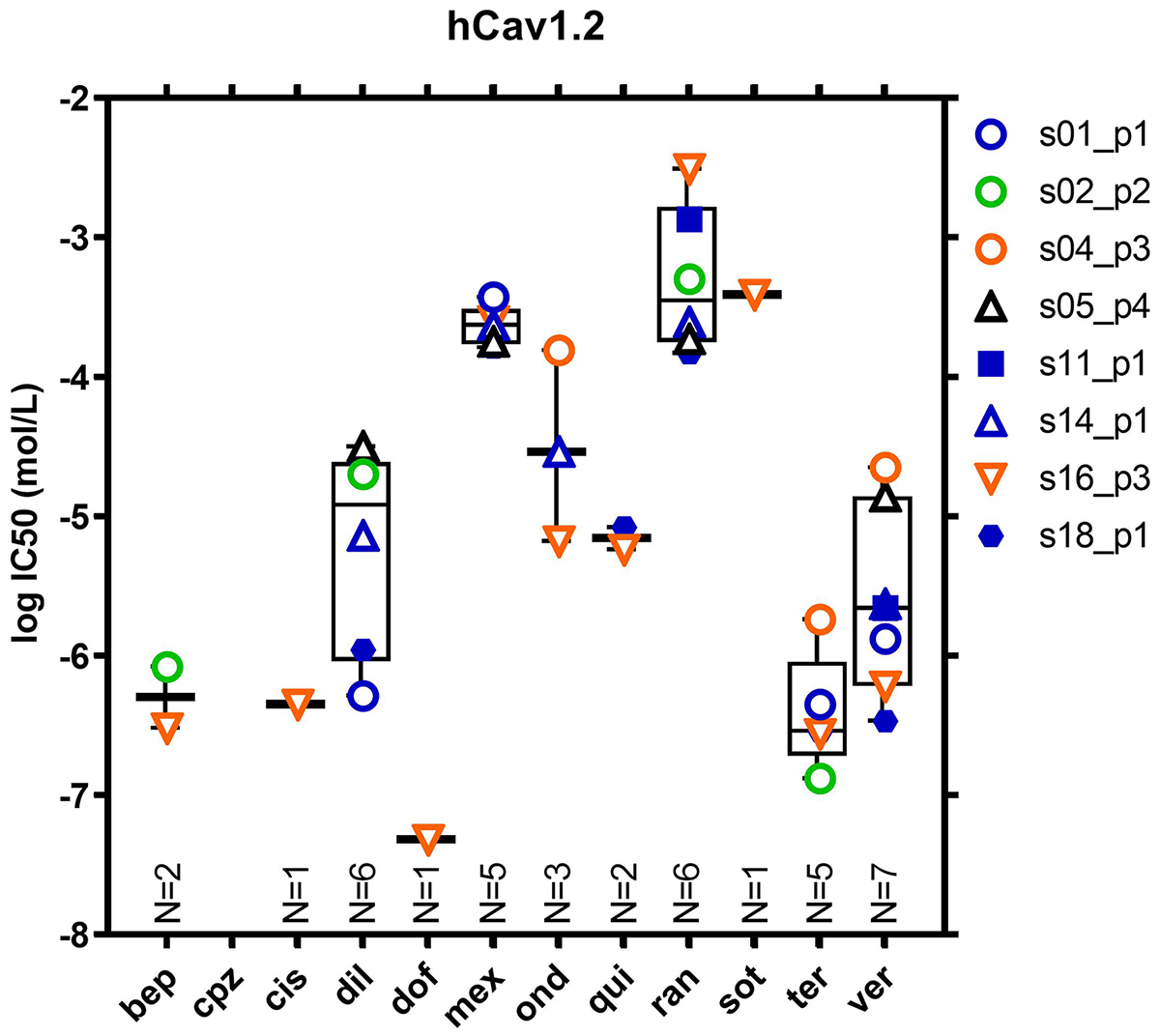

Supplement: Supplementary file 4 — Supporting Information4. [file 41598_2020_62344_MOESM4_ESM.tiff]

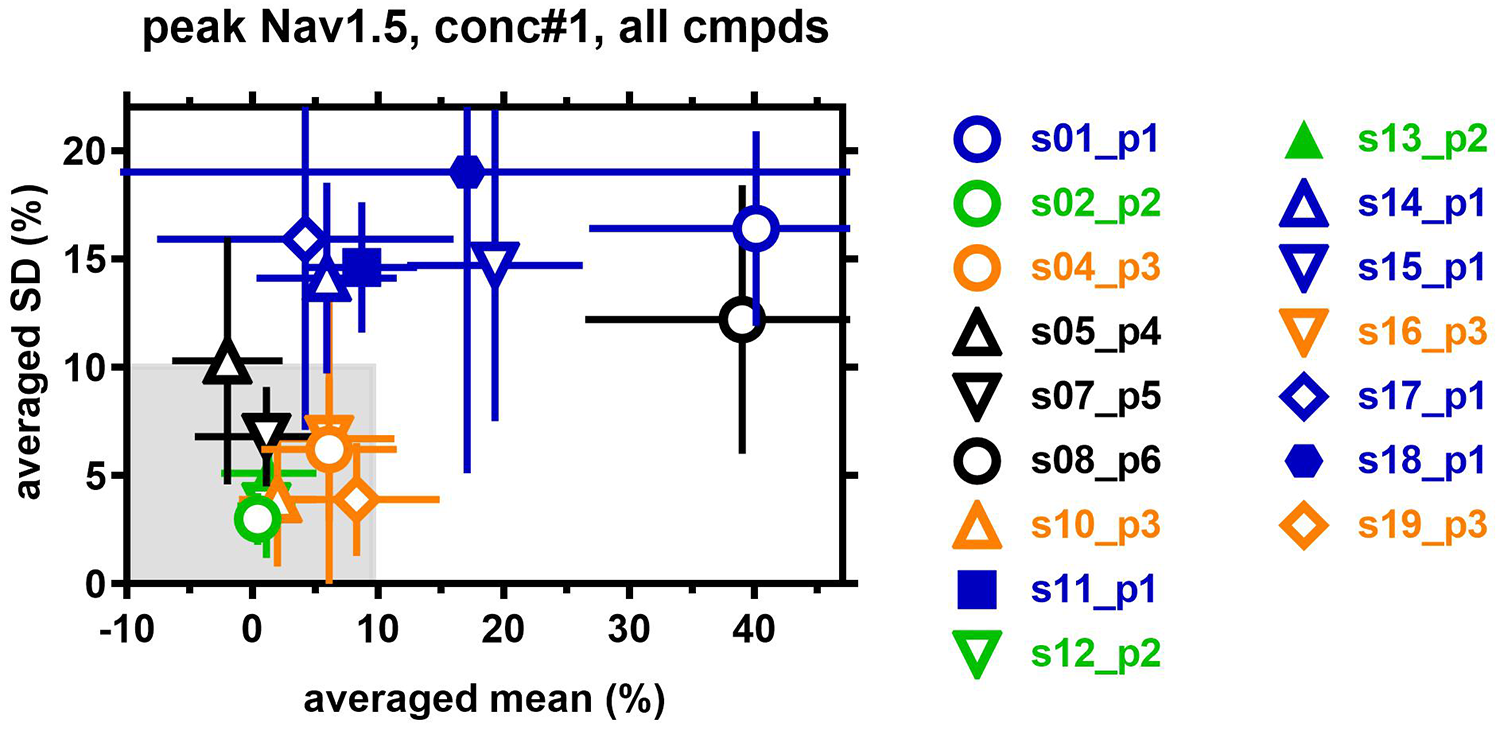

Supplement: Supplementary file 5 — Supporting Information5. [file 41598_2020_62344_MOESM5_ESM.tiff]

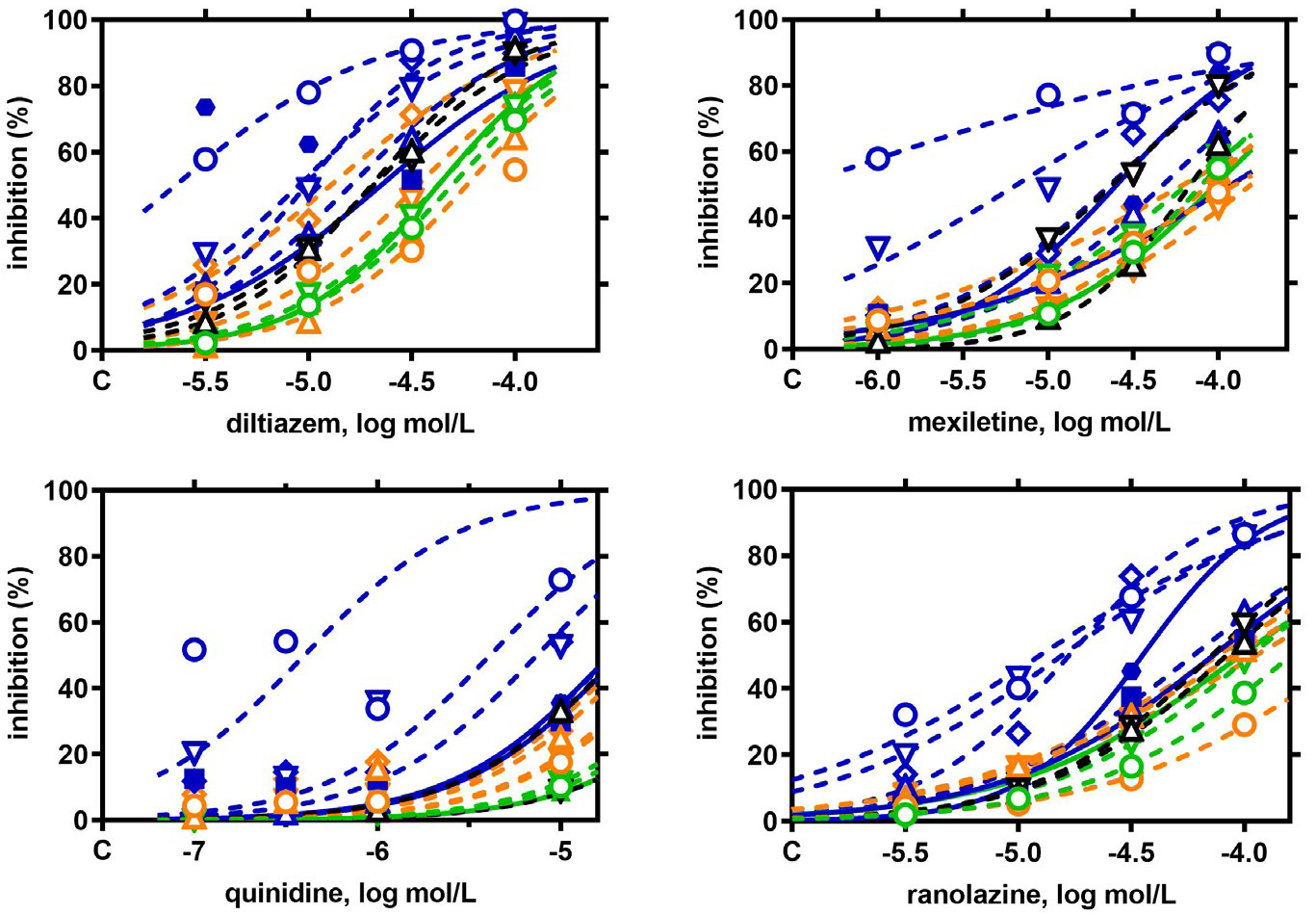

Supplement: Supplementary file 6 — Supporting Information6. [file 41598_2020_62344_MOESM6_ESM.tiff]

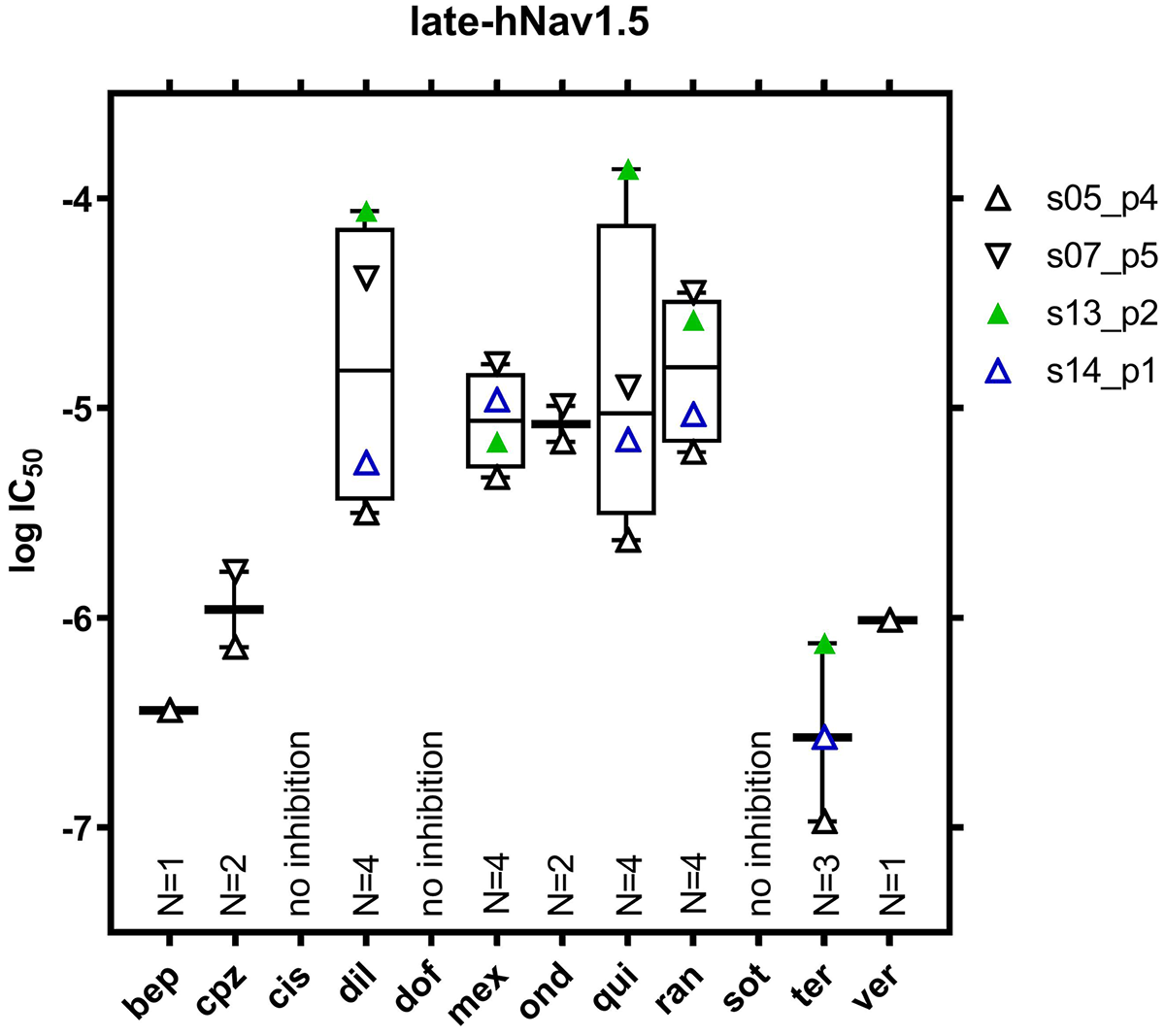

Supplement: Supplementary file 7 — Supporting Information7. [file 41598_2020_62344_MOESM7_ESM.tiff]

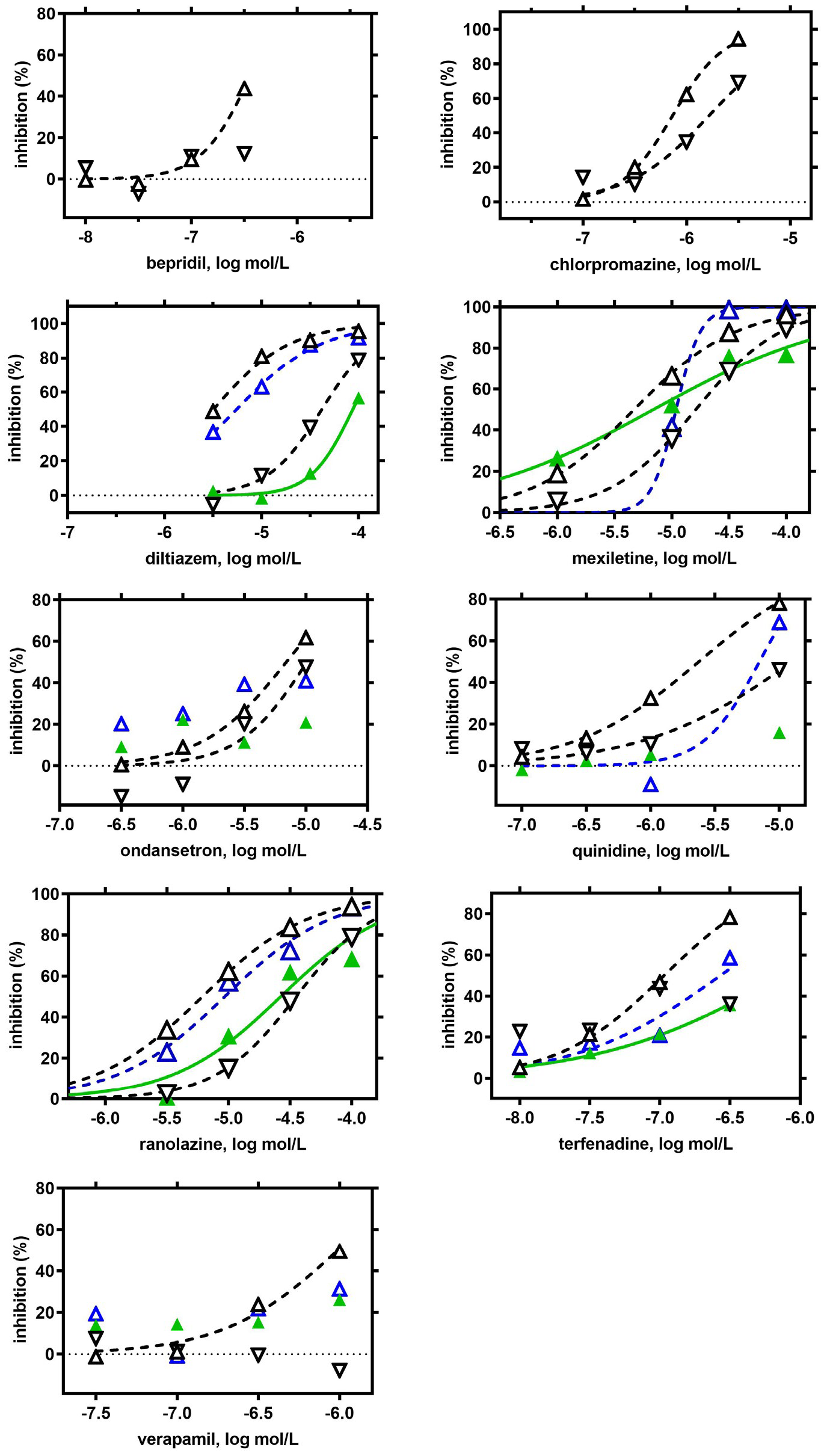

Supplement: Supplementary file 8 — Supporting Information8. [file 41598_2020_62344_MOESM8_ESM.tiff]

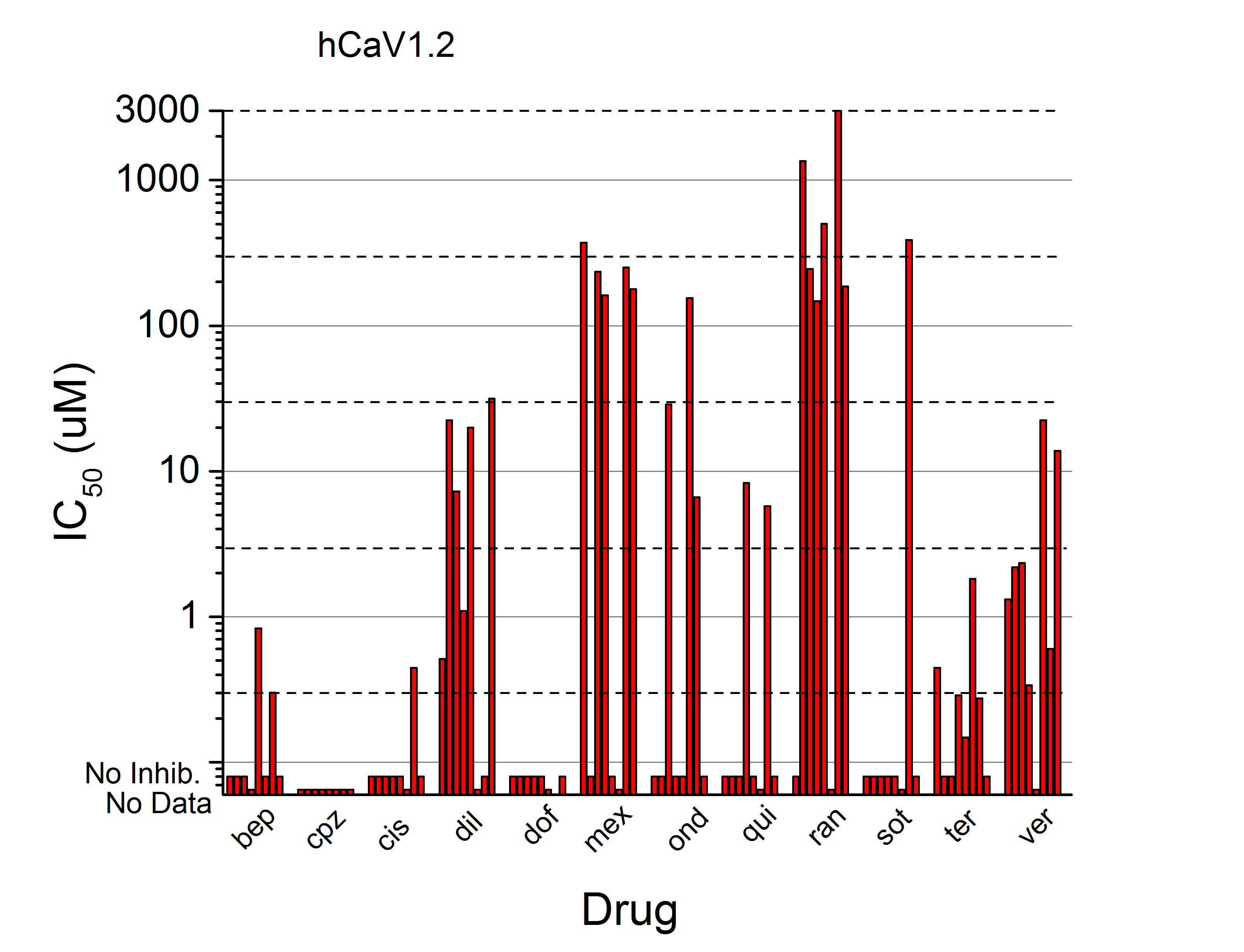

Supplement: Supplementary file 9 — Supporting Information9. [file 41598_2020_62344_MOESM9_ESM.jpg]

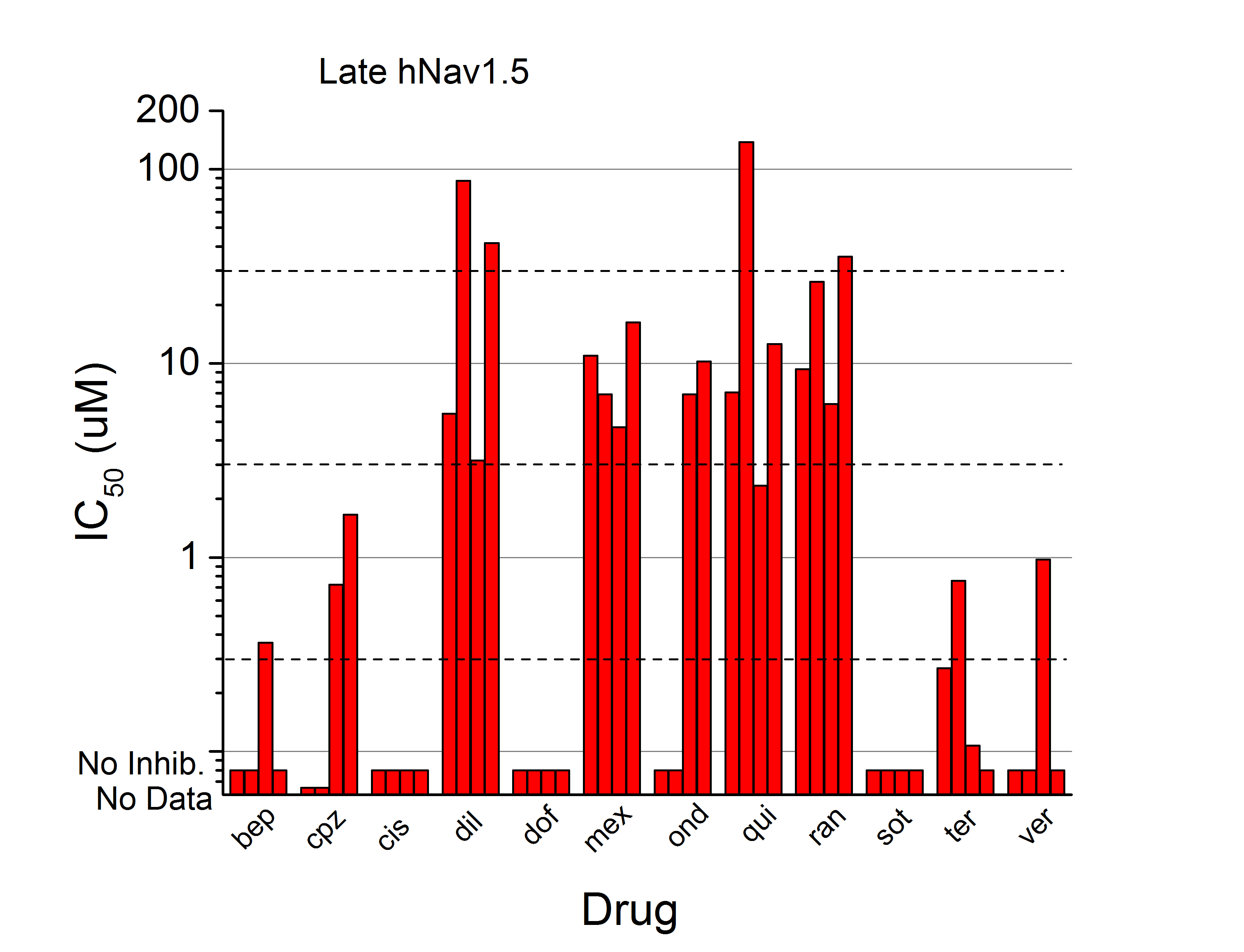

Supplement: Supplementary file 10 — Supporting Information10. [file 41598_2020_62344_MOESM10_ESM.jpg]
